# Deep Brain Stimulation Induces Antidepressant Effects by Restoring High‐Fidelity Communication in the BNST‐NAc Circuit

**DOI:** 10.1002/advs.202521943

**Published:** 2026-03-09

**Authors:** Xin Lv, Yu Cao, Yuhan Wang, Yunhao Wu, Yingying Zhang, Kuanghao Ye, Xian Qiu, Qingfang Sun, Liuguan Bian, Halimureti Paerhati, Valerie Voon, Shikun Zhan, Bomin Sun

**Affiliations:** ^1^ Department of Neurosurgery Ruijin Hospital Shanghai Jiao Tong University School of Medicine Shanghai China; ^2^ Center of Functional Neurosurgery Ruijin Hospital Shanghai Jiao Tong University School of Medicine Shanghai China; ^3^ Shandong Mental Health Center Shandong China; ^4^ Department of Neurosurgery Beijing Tiantan Hospital Capital Medical University Beijing China; ^5^ Neural and Intelligence Engineering Centre Institute of Science and Technology for Brain‐Inspired Intelligence Fudan University Shanghai China; ^6^ Department of Nursing Ruijin Hospital Shanghai Jiao Tong University Shanghai China; ^7^ Shanghai Jiao Tong University School of Nursing Shanghai China; ^8^ Department of Psychiatry Addenbrookes Hospital University of Cambridge Cambridge UK

**Keywords:** deep brain stimulation, excitation inhibition balance, GABAergic projection, local field potential, striatum, treatment‐resistant depression

## Abstract

Deep brain stimulation (DBS) for treatment‐resistant depression (TRD) is challenged by significant individual variability in efficacy and unclear neural circuit mechanisms. To address this, a cross‐species, multi‐level electrophysiological study was conducted to elucidate the core underlying pathophysiology and reveal the precise therapeutic mechanisms of DBS. Based on the clinical trial (NCT04530942), this study focuses on the bed nucleus of the stria terminalis‐nucleus accumbens (BNST‐NAc) circuit, and it is hypothesized that the fundamental pathology of the depressive state lies in the persistent hyperactivity of BNST neurons, which disrupts the high‐fidelity signal communication capacity of this circuit. In a mouse model, a key communication pattern, inhibitory period isolated spikes (IPIS), was first identified within the excitation/inhibition (E/I) cycle. This pattern involves slow‐wave oscillations creating a high signal‐to‐noise ratio window for the firing of single or few action potentials, thereby enabling efficient inter‐regional communication. Subsequently, it was found that chronic stress‐induced pathological hyperactivity of BNST neurons in stress‐susceptible animals specifically disrupts the inhibitory periods of network activity, thereby dismantling IPIS‐mediated cross‐regional neural synchrony and leading to circuit dysfunction. The therapeutic mechanism of DBS was verified to involve precisely suppressing the pathological hyperactivity of the BNST, thereby restoring the network's inhibitory periods and re‐establishing the efficient signal transmission pathway mediated by IPIS. In a closed‐loop DBS paradigm, only continuous stimulation and stimulation precisely locked to the inhibitory periods produced antidepressant effects and most effectively restored cross‐regional communication. Furthermore, in a cohort of human TRD patients, local field potential (LFP) data were recorded during BNST‐NAc DBS treatment, and LFP biomarkers corresponding to the restoration of circuit function were identified. To more directly validate changes in E/I cycles in the human brain, an innovative cross‐species algorithm was developed to decode functional excitatory and inhibitory periods from macroscopic human LFP signals. It was confirmed that the therapeutic response to DBS is associated with an increased proportion of inhibitory periods and the functional recovery of the BNST‐NAc circuit, providing direct quantitative evidence for the theory that DBS restores E/I balance in the human brain. Finally, a double‐blind, crossover randomized controlled trial (RCT) involving 18 participants confirmed that active DBS clinically alleviated depressive symptoms (an average of 9.4 reduction). Open‐label data were used for model development, while RCT data served as an independent validation set. Model ablation study within a deep learning framework confirmed that E/I cycle features provide significantly higher informative value than spectral models, establishing these dynamics as the primary electrophysiological determinants of the clinical state. This study integrates mechanistic research with clinical validation, providing evidence for precision and personalized closed‐loop DBS therapy.

## Graphical abstract

This cross‐species study reveals that pathological hyperactivity of BNST neurons in depressive states disrupts inhibitory period and isolated spikes in the BNST‐NAc circuit. DBS achieves its antidepressant effects by precisely restoring network inhibitory periods and high‐fidelity signal transmission. Validated by a clinical RCT, this research establishes E/I balance as a core mechanism and objective biomarker for precision neuromodulation in TRD.

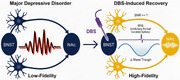

## 1 Introduction

Approximately 30% of patients with depression do not respond adequately to standard treatment protocols and are classified as having treatment‐resistant depression (TRD). TRD is defined as the failure to achieve sufficient clinical improvement after trials of at least two antidepressant medications of adequate dose and duration within a single depressive episode. This patient population persistently faces functional impairment, reduced quality of life, and increased risk of suicide. Therefore, the development of new therapeutic strategies is urgently needed [1]. Deep brain stimulation (DBS) offers a potential treatment option for patients with TRD. By modulating dysfunctional neural circuits, DBS has demonstrated significant and sustained antidepressant effects in some TRD patients [2, 3]. Key neuroanatomical targets for DBS in the TRD include the subcallosal cingulate gyrus (SCG), the nucleus accumbens (NAc), the ventral capsule/ventral striatum (VC/VS, including the anterior limb of the internal capsule [ALIC] and the bed nucleus of the stria terminalis [BNST]), the medial forebrain bundle (MFB), the lateral habenula (LHb), and the inferior thalamic peduncle (ITP) [4]. Frequencies are generally applied between 100 and 185 Hz (130 Hz is the most common); voltages range between 1.5 and 10.5 V; and pulse widths range between 60 and 450 µs.

However, the therapeutic efficacy of DBS shows considerable inter‐individual variability, which suggests our understanding of the neuropathological heterogeneity of depression and the neural mechanisms of DBS action is insufficient [4, 5]. This variability is likely driven by a complex interplay of factors, including optimal target selection, stimulation parameter settings, and the high heterogeneity of disease pathology across patients. This realization has prompted a shift in DBS research strategies from traditional anatomical target localization to circuit‐based modulation [6]. Effective treatment may not depend on a single uniform anatomical target but rather on precise interventions targeting pathophysiological activity within functionally abnormal circuits in specific patient subgroups. Consequently, a core challenge in current research is the identification of objective biomarkers that can predict and track the therapeutic efficacy of DBS [4]. Emerging evidence suggests a critical need to shift towards functional circuit restoration, identifying and modulating the pathological physiological states within these complex anatomical targets.

In this context, the neural circuit between the Bed Nucleus of the Stria Terminalis (BNST) and the Nucleus Accumbens (NAc) is an important subject for exploring more effective DBS targets and deepening the understanding of circuit mechanisms in depression [7]. As a core component of the extended amygdala, the BNST serves as a critical hub connecting the limbic system, the cognitive control network, and the stress response system. It primarily processes sustained, unpredictable threats and generates anxiety‐like states [8]. Notably, the GABAergic inhibitory projection from the BNST to the NAc forms a direct pathway connecting the brain's stress‐aversive and reward‐motivation systems [9, 10]. This anatomical foundation provides a neural circuit‐level explanation for the core symptoms of depression, such as anhedonia and anxiety [11]. A key hypothesis derived from this foundation posits that the essential pathophysiology of depression may involve dysfunction of the BNST‐NAc circuit, wherein excessive negative emotional signals from the BNST exert pathological inhibition on the reward and motivational functions of the NAc. Therefore, the BNST‐NAc circuit is not only a relevant model for studying the pathophysiology of depression but also provides a potential intervention target with a clear mechanistic basis for neuromodulation therapies like DBS. Early clinical studies have already validated the potential antidepressant effect of the BNST as a DBS target [12].

To understand how DBS modulates the BNST‐NAc circuit, we must first comprehend its underlying mechanisms at the neuronal level. The fundamental action of DBS is to alter the transmembrane potential of neural elements through the application of an extracellular electric field. This polarization modulates the kinetics of voltage‐gated ion channels, including sodium, potassium, and calcium currents. Which result in a complex pattern of activity that typically involves the generation of axonal action potentials coupled with the local suppression of somatic firing [13, 14, 15, 16, 17, 18]. Mechanistically, this manifests at the cellular level as soma‐axon decoupling: somatic firing is suppressed via depolarization blockade, while axonal projections are robustly driven at the stimulation frequency. Locally at the stimulation site, high‐frequency stimulation primarily inhibits the firing activity of neuronal cell bodies [19, 20]. This inhibitory effect may be achieved through a depolarization block, where neurons are kept in a state of sustained depolarization and cannot repolarize, or through the activation of local inhibitory interneurons [21, 22]. At the same time, because the excitation threshold of myelinated axons is lower than that of neuronal cell bodies, DBS can more effectively and directly activate their axons while inhibiting local somas, allowing signals to continue propagating downstream [15, 23]. Therefore, DBS reconfigures the dynamics of the entire network by imposing an informational interference at a critical node of a pathological network. This process produces clinical effects at the behavioral level that are analogous to a functional lesion of the pathological function of that node [17].

Anatomical studies show that the BNST‐NAc pathway mainly originates from somatostatin‐positive (SOM+) neurons in the anterodorsal BNST (adBNST) and projects to the Nucleus Accumbens Shell (sNAc), where it directly synapses onto parvalbumin‐positive (PV+) inhibitory interneurons [9]. These PV+ interneurons are critical pacemakers of gamma oscillations within the NAc, and their activity is vital for local information processing. In stressed animal models exhibiting depressive‐like behaviors, both the projection neurons in the adBNST and the PV+ interneurons in the sNAc display pathological hyperexcitability [24]. This communication failure in the BNST(SOM^+^) → NAc(PV^+^) pathway leads to insufficient inhibition of NAc(PV^+^) interneurons, which in turn drives observable abnormalities in NAc gamma oscillations. When applied to the BNST, DBS is theorized to exert a dual effect on the circuit: it inhibits the cell bodies of adBNST projection neurons while simultaneously activating their axons projecting to the NAc. As axonal activation is a more potent and widespread effect, the net functional output of the circuit is predominantly determined by this axonal activation.

Building on a noise‐cancellation and rhythm‐restoration hypothesis for DBS modulation [15]. We posit that in the depressive state, disorganized hyperactivity of BNST neurons acts as pathological ‘noise’ that disrupts the circuit's physiologic Inhibitory Periods. DBS addresses this by exerting a dual biophysical effect (McIntyre et al., 2004): it suppresses the pathological somatic firing of BNST neurons and eliminates the noise source while simultaneously driving their GABAergic axons projecting to the NAc. This DBS‐driven axonal output provides a potent, synchronized inhibitory signal that actively enforces silence in the NAc during critical time windows. Thus, DBS restores the high signal‐to‐noise ratio necessary for IPIS‐mediated communication by overriding pathological noise with a restored inhibitory tone. The DBS‐driven inhibitory signal is expected to effectively suppress the pathological activity of NAc (PV+) interneurons, thereby normalizing their function. Ultimately, by correcting this dysfunction at the microcircuit level, DBS may fundamentally alleviate the associated depressive symptoms [25].

The excitatory/inhibitory balance (E/I balance) is one of the theoretical frameworks that bridges the above theory from the neuronal level to macroscopic depressive symptoms [26, 27]. Appropriate neuronal excitability requires regulation by GABAergic inhibitory interneurons. This architecture ensures that neurons can integrate input signals, maintain an optimal membrane potential, and generate adaptive responses [28, 29, 30, 31]. The excitatory/inhibitory balance can be measured in multiple ways, including the direct ratio of neurotransmitters, the excitatory and inhibitory cycles of neuronal firing (UP‐states and DOWN‐states), and indirectly through parameters such as the power spectral density slope in field potentials and neural oscillation activity [32, 33, 34]. Substantial evidence indicates that the core pathophysiology of major depressive disorder (MDD) lies in a persistent E/I imbalance within emotional circuits, specifically manifesting as a failure of inhibitory control and an overall shift towards excitation [29, 30, 35, 36, 37]. DBS may reverse this pathological mechanism. Related theories include the overriding of pathological rhythms, preferential recruitment of inhibitory interneurons, and induction of therapeutic neuroplasticity. However, none of these theories can be integrated with the direct effects of DBS on subcortical neurons. A systematic investigation at the level of the direct electrical effects of DBS is required to explore the direct mechanisms of its antidepressant action [38, 39, 40].

To resolve the dysfunction and repair mechanisms of this circuit on a finer temporal scale, this study is based on a hypothesized circuit‐level medium for communication, which is defined asinhibitory period isolated spikes (IPIS). This theory suggests that during the trough of a slow‐wave oscillation, background neural noise is reduced. This creates a favorable window with a high signal‐to‐noise ratio (SNR) for the firing of single or a few action potentials [41]. The isolation mechanism ensures the high‐fidelity maintenance of inter‐regional information. The central hypothesis of this study is that in a depressive state, stress‐induced persistent hyperactivity of BNST neurons disrupts the inhibitory periods of network activity. This disruption impairs IPIS‐mediated communication from the BNST to the NAc, ultimately leading to circuit dysfunction. Effective DBS treatment is proposed to produce its antidepressant effects by suppressing the pathological over‐firing of the BNST and restoring the network's inhibitory periods, thereby re‐establishing IPIS‐mediated BNST→NAc signal transmission. These microscopic neural firing patterns are expected to have corresponding electrophysiological signatures in macroscopic LFP recordings.

Although translating discoveries from basic neuroscience to clinical psychiatric disorders presents challenges, the fundamental electrophysiological mechanisms of mammalian neural circuit operation and the neuronal synaptic architecture of the BNST‐NAc circuit exhibit high cross‐species conservation. Therefore, compared to complex behavioral phenotypes, features based on conserved electrophysiological mechanisms can provide a more reliable foundation for cross‐species translational research. The design of this study follows this principle. It will first use single‐unit recording techniques in a mouse model to analyze the impairment and restoration process of the IPIS communication mechanism. Subsequently, the microscopic findings from the animal model will serve as a reference standard to guide the identification of homologous biomarkers in LFP data from human TRD patients. In summary, this study aims to elucidate the dysfunction of the BNST‐NAc circuit in depression and reveal the potential mechanisms by which DBS restores its function through a cross‐species, multi‐level electrophysiological investigation. The research plan will first characterize the electrophysiological properties of IPIS and its role in cross‐regional signal transmission in healthy mice. Second, within a learned helplessness model of depression, it will test whether chronic stress disrupts IPIS‐mediated communication by inducing hyperactivity in BNST neurons and will use a closed‐loop DBS paradigm to verify if its antidepressant effect correlates with the restoration of this communication mechanism. Third, it will apply the mechanistic insights gained from the animal model to a cohort of human TRD patients to identify homologous LFP biomarkers associated with clinical state and DBS efficacy. This study intends to leverage the evolutionary conservation of nuclear neuronal composition to develop and validate a cross‐species machine learning model capable of inferring network inhibitory and excitatory periods from human LFPs and to test whether DBS specifically restores functional connectivity within these inhibitory periods. Finally, through a 2‐year clinical follow‐up and a double‐blind, crossover randomized controlled trial, this study will systematically validate the robustness of these biomarkers and their role in distinguishing depressive states, thereby providing a biological basis for achieving precision DBS therapy.

## 2 Results

### 2.1 Characteristics of Inhibitory Period Isolated Spikes in BNST‐NAc Circuit Neurons and Their Role in Mediating Cross‐Regional Signal Transmission

**FIGURE 1:**
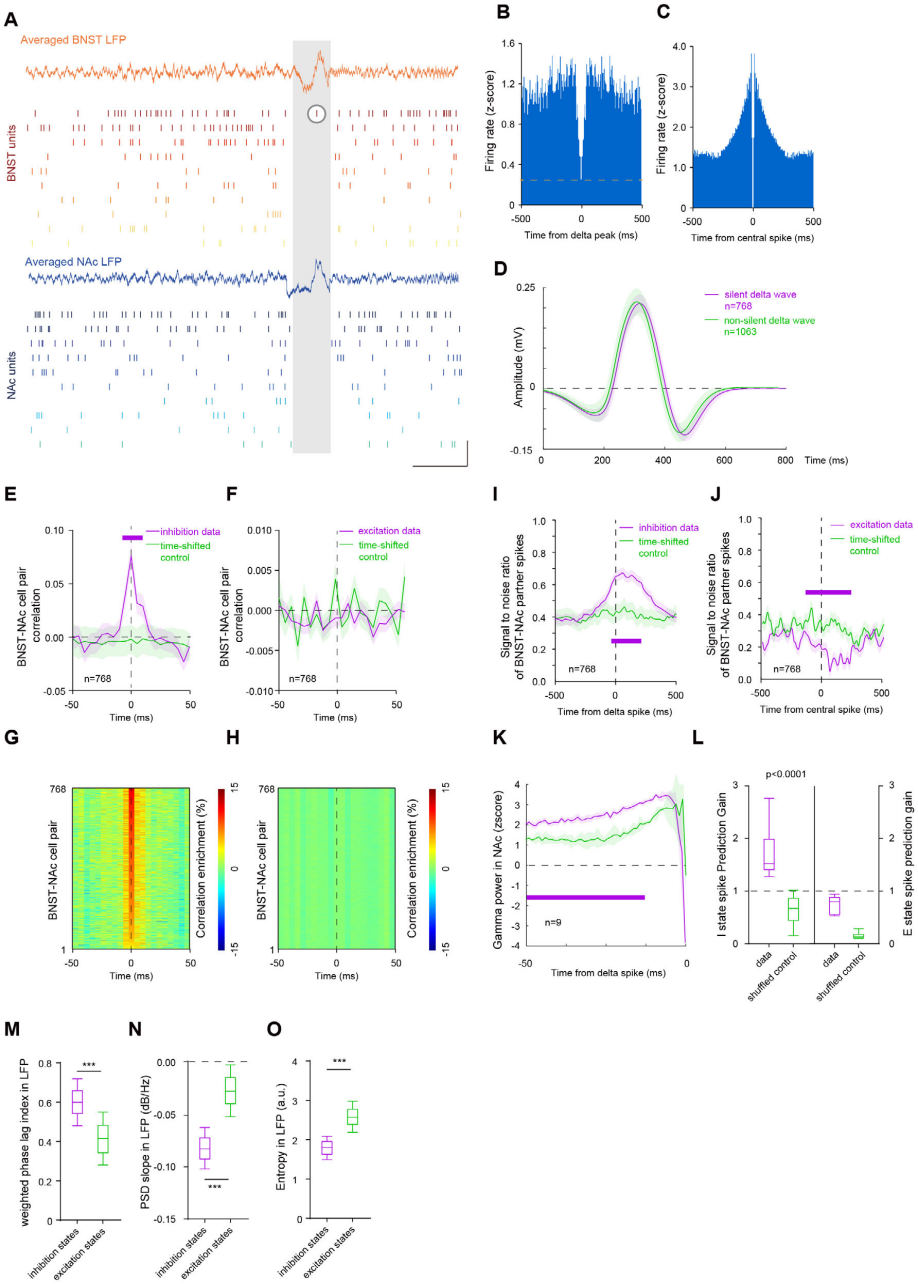
Characteristics of inhibitory period isolated spikes from partner cells in the BNST‐NAc circuit and their role in mediating cross‐regional signal transmission. (A) Schematic of IPIS in the BNST‐NAc circuit. Curves indicate the multi‐channel averaged LFPs simultaneously recorded from the BNST and NAc. Vertical ticks indicate the raster plot of simultaneously recorded spikes. The gray box highlights an inhibitory period coinciding with a cross‐regionally synchronized delta wave. The circles mark inhibitory period isolated spikes in the BNST‐NAc circuit. Calibration bars: horizontal = 1000 ms, vertical = 1 mV. (B) Mean peri‐event time histogram of the firing rate of BNST units centered on inhibition states. The bottom yellow dashed line indicates non‐zero firing occurring at the delta peak. (C) Mean peri‐event time histogram of the firing rate of BNST units centered on excitation states. (D) Comparison of delta waveforms during inhibitory periods vs. noninhibitory periods in the BNST‐NAc circuit. (E) Spike cross‐correlation between BNST and NAc mediated by peri‐IPIS (curves and shaded areas, mean ± SEM; horizontal purple line indicates Monte Carlo test, *p* < 0.05; *n* = 768 events in 9 mice). (F) Spike cross‐correlation between BNST and NAc mediated by center spikes of the peri‐excitatory period (curves and shaded areas, mean ± SEM; *n* = 768 events in 9 mice). (G) Enrichment in positive correlations for the BNST‐NAc spikes shown in (E). (H) Enrichment in positive correlations for the BNST‐NAc spikes in (F). (I) Signal‐to‐noise ratio (SNR) in the BNST and NAc mediated by peri‐IPIS, compared to a time‐shifted control (curves and shaded areas, mean ± SEM; horizontal purple line indicates Monte Carlo test, *p* < 0.05; *n* = 768 events in 9 mice). (J) Signal‐to‐noise ratio (SNR) in the BNST and NAc mediated by center spikes of the peri‐excitatory period, compared to a time‐shifted control (curves and shaded areas, mean ± SEM; horizontal purple line indicates Monte Carlo test, *p* < 0.05; *n* = 768 events in 9 mice). (K) Comparison of NAc gamma power around peri‐IPIS vs. peri‐excitatory period center spikes in the BNST‐NAc circuit (curves and shaded areas, mean ± SEM; horizontal purple line indicates Monte Carlo test, *p* < 0.05; *n* = 9 mice). (L) Performance of a GLM trained to predict IPIS(left, unpaired t‐test, *n* = 9, t = 5.917, df = 16) and excitatory period center spikes (right, unpaired t‐test, *n* = 9, t = 9.787, df = 16) based on preceding NAc gamma activity (−50 ms)). A value >1 indicates a positive contribution of NAc gamma activity to the prediction; a value <1 indicates a negative contribution. (M) Cross‐regional weighted phase‐lag index (wPLI) of LFPs during inhibitory and excitatory periods in the BNST‐NAc circuit (paired t‐test, *p* < 0.0001, t = 66.06, df = 16, n = 768 events in 9 mice). (N) PSD slope of the BNST LFP during inhibitory and excitatory periods (*p* < 0.0001, t = 75.85, df = 16, paired t‐test, n = 768 events in 9 mice). (O) Sample entropy of the BNST LFP during inhibitory and excitatory periods (*p* < 0.0001, t = 99.66, df = 16, paired t‐test, *n* = 768 events in 9 mice).

We recorded BNST‐NAc activity in nine mice during the resting state and identified spike activity occurring in conjunction with BNST delta waves (Figure 1A and Figure S1A). Isolated spikes during the inhibition phase are thought to mediate key high signal‐to‐noise ratio periods, contrasting with spikes occurring during the excitation phase (Figure 1B,C). Among all recorded neurons, only about one‐third of recorded neurons exhibited inhibitory period isolated spikes, suggesting that these spikes may be confined to a specific neuronal subset within the BNST‐NAc circuit. Consistent with previous reports, these delta‐timed spikes did not preferentially occur during specific delta waves with unique properties; there was no significant difference in the waveform of delta waves in which spikes were detected vs. those in which they were not (Figure 1D).

Therefore, we extracted inhibitory period isolated spikes and the center spikes of the excitatory periods to analyze their capacity for mediating cross‐regional signal synchronization (Figure 1E–H). The BNST‐NAc spike correlation was significantly positive within a 50 ms window surrounding the inhibitory period, isolated spikes, and was stronger than that of the center spikes of the excitatory period, which served as a control. Furthermore, owing to the relatively silent background of the inhibitory period, these isolated spikes induced information exchange with a significantly higher signal‐to‐noise ratio (Figure 1I,J, and Figure S1B,C).

We next sought to assess whether inhibitory period isolated spikes were characterized by specific spectral features in the BNST‐NAc circuit's LFP. We compared the LFP component power surrounding isolated spikes and found that, compared to center spikes of the excitatory period, gamma power in the NAc was elevated prior to the inhibitory period of isolated spikes (Figure 1K). BNST neurons exhibiting significant spike‐to‐spike temporal correlations with specific NAc units were defined as partner cells. Subsequently, a Generalized Linear Model (GLM) analysis was employed to independently verify if NAc gamma activity could predict the occurrence of isolated spikes in these neurons, serving as a validation of cross‐regional information coupling (Figure 1L). The behavioral relevance of this finding implies that inhibitory period isolated spikes may be accompanied by changes in LFP‐related features. Accordingly, by examining the LFP's connectivity, complexity, and aperiodic components, we found evidence that inhibitory period isolated spikes mediate cross‐regional signal transmission through a high signal‐to‐noise ratio time window (Figure 1M–O).

### 2.2 Stress‐Induced Depression‐Like Phenotype Disrupts Inhibitory Period Isolated Spikes via BNST Neuron Hyperactivity

**FIGURE 2:**
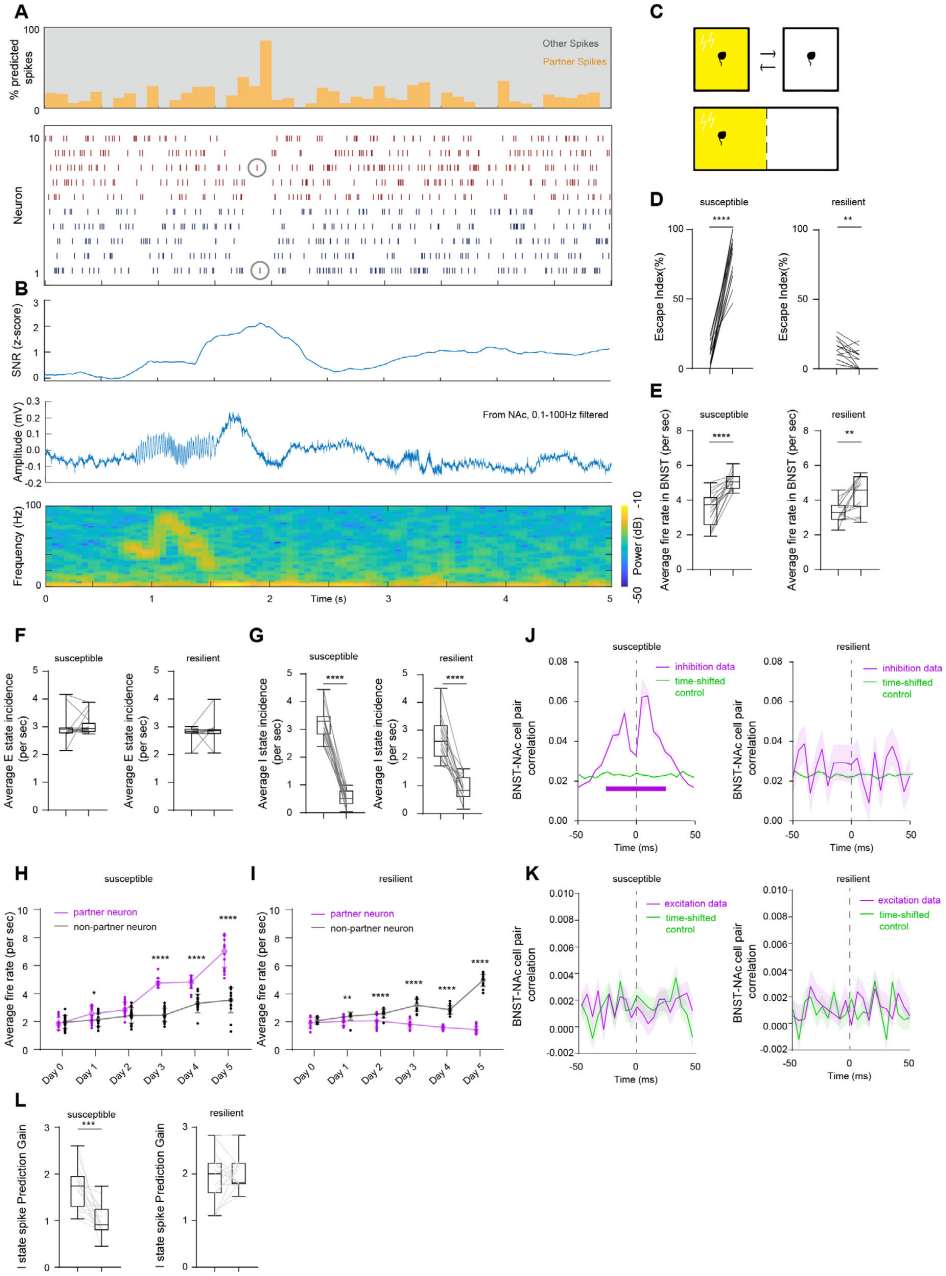
Stress‐induced depression‐like phenotype disrupts inhibitory period isolated spikes via BNST neuron over‐firing. (A) Simultaneous recording of BNST‐NAc activity around an isolated spike during a cross‐regional inhibitory period. (Top) Proportion of NAc spikes predicted by the firing of BNST units (partner spikes). (Bottom) Raster plot of spikes from 10 simultaneously recorded units from BNST (red) and NAc (blue). (B) Features of the NAc LFP recorded simultaneously with (A). (Top) Signal‐to‐noise ratio (SNR) of the NAc LFP. (Middle) Raw waveform of the NAc LFP after 0.1–100 Hz bandpass filtering. (Bottom) Spectrogram of the NAc LFP after 0.1–100 Hz bandpass filtering. (C) Schematic of the learned helplessness paradigm used to induce despair‐like behavior in mice via exogenous stress. (D) Stress‐susceptible mice (left, paired t‐test, premodeling vs. post‐modeling, *n* = 17, *p* < 0.0001, t = 16.44, df = 16) and stress‐resilient mice (right, paired t‐test, pre‐modeling vs. postmodeling, *n* = 15, *p* = 0.0017, t = 3.872, df = 14) show escape failures after 5 consecutive days of footshocks, indicating depression‐like behavior. (E) Mean firing rate of BNST neurons in stress‐susceptible mice (left, paired t‐test, premodeling vs. postmodeling, *n* = 17, *p* < 0.0001, t = 6.418, df = 16) and stress‐resilient mice (right, paired t‐test, premodeling vs. postmodeling, *n* = 15, *p* = 0.0045, t = 3.373, df = 14). (F) Excitatory period firing rate of the BNST‐NAc circuit in stress‐susceptible mice (left, paired t‐test, pre‐modeling vs. post‐modeling, *n* = 17, *p* = 0.4978, t = 0.6937, df = 16) and stress‐resilient mice (right, paired t‐test, premodeling vs. postmodeling, n = 15, 0.5958, t = 0.5428, df = 14). (G) Inhibitory period firing rate of the BNST‐NAc circuit in stress‐susceptible mice (left, paired t‐test, premodeling vs. postmodeling, *n* = 17, *p* < 0.0001, t = 18.54, df = 16) and stress‐resilient mice (right, paired t‐test, premodeling vs. postmodeling, *n* = 15, *p* < 0.0001, t = 8.368, df = 14). (H) Firing rate changes of BNST partner neurons (purple) and nonpartner neurons (black) in stress‐susceptible mice during the modeling process. Data are presented as mean ± SEM. Statistical significance was assessed using a linear mixed‐effects model (LMM) which revealed a significant Group × Day interaction (F_5192_ = 42.93, *p* < 0.0001), followed by simple effects analysis for group comparisons at each time point (Day 0: *p* = 0.5649; Day 1: *p* = 0.0363; Day 2: *p* = 0.0903; Day 3: *p* < 0.0001; Day 4: *p* < 0.0001; Day 5: *p* < 0.0001). (I) Firing rate changes of BNST partner neurons (purple) and non‐partner neurons (black) in stress‐resilient mice during the modeling process. Data are presented as mean ± SEM. Statistical analysis using a linear mixed‐effects model (LMM) revealed a highly significant Group × Time interaction (F_5600_ = 42.93, *p* < 0.0001). Posthoc simple effects analysis showed no difference at baseline (Day 0: *p* = 0.4884), but revealed significant divergence starting from Day 1 (Day 1: t_98_ = −3.01, *p* = 0.0033; Day 2: t_98_ = −7.36, *p* < 0.0001; Day 3: t_98_ = −15.88, *p* < 0.0001; Day 4: t_98_ = −20.21, *p* < 0.001; Day 5: t_98_ = −37.33, *p* < 0.0001). (J) Spike cross‐correlation between BNST and NAc mediated by peri‐IPIS in stress‐susceptible mice (left, curves and shaded areas, mean ± SEM; horizontal purple line indicates Monte Carlo test, *p* < 0.05; *n* = 791 events in 17 mice) and stress‐resilient mice (right, curves and shaded areas, mean ± SEM; *n* = 791 events in 17 mice). (K) Spike cross‐correlation between BNST and NAc mediated by peri‐excitatory period center spikes in stress‐susceptible mice (left, curves and shaded areas, mean ± SEM; *n* = 1261 in 15 mice) and stress‐resilient mice (right, curves and shaded areas, mean ± SEM; *n* = 1261 in 15 mice). (L) Performance of a GLM trained to predict IPIS based on preceding NAc gamma activity (−50 ms) in stress‐susceptible mice (left, paired t‐test, *n* = 17, *p* = 0.0003, t = 4.660, df = 16) and stress‐resilient mice (right, paired t‐test, *n* = 15, *p* = 0.6268, t = 0.4972, df = 14).

Consistent with previous reports, a key function of inhibitory period isolated spikes is to isolate specific cortical computations for precise cross‐regional signal transmission, creating high signal‐to‐noise ratio time windows that are shielded from interference. To test whether inhibitory periods preferentially silence cortical activity unrelated to ongoing hippocampus‐cortical dialogues, we classified individual BNST spikes: individual BNST spikes were classified based on their temporal relationship with NAc firing: spikes occurring within a significant correlation window relative to NAc partner cell activity were defined as partner spikes, while those falling outside these windows were termed other spikes. This classification was performed strictly using neuronal firing timestamps, ensuring that subsequent analyses of NAc LFP features remained statistically independent. The signal‐to‐noise ratio surrounding partner spikes peaked during delta waves, a result of the selective silencing of non‐partner activity during these periods (Figure 2A,B).

We then aimed to explore how an exogenous stress‐induced depression‐like phenotype affects this neuronal activity pattern. For this, we subjected an additional 32 mice to a learned helplessness paradigm (Figure 2C). After five consecutive days of footshock training, 17 mice exhibited escape‐avoidance failure, a depression‐like behavior, and were defined as the stress‐susceptible group (Figure 2D). The remaining 15 mice, which did not display depression‐like behavior after the footshocks, were defined as the stress‐resilient group (Figure 2D). Subsequent analyses focused on the comparison between these two groups.

Consistent with previous studies, exogenous stress increased the overall firing frequency of the BNST in both susceptible and resilient groups, but this increase was significant only in the susceptible group. In the resilient group, the firing rate changes in some individuals were inconsistent with the overall trend (Figure 2E). This led us to investigate whether the changes in firing rate were concentrated in specific temporal and spatial states. By calculating the firing rates during excitatory and inhibitory periods, we observed that exogenous stress had no effect on the excitatory period firing rate in either group but significantly disrupted the inhibitory period firing patterns (Figure 2F,G). Furthermore, by categorizing neurons into partner neurons and non‐partner neurons, we found that in the susceptible group, exogenous stress induced a significant increase in the firing of both neuron types, with the effect being more pronounced in partner neurons (Figure 2H). In the resilient group, stress did not affect partner neurons; only the changes in nonpartner neurons were consistent with the susceptible group (Figure 2I). This suggests that the disruption of the inhibitory period in the susceptible group is primarily attributable to the over‐firing of partner neurons, whereas in the resilient group, it is mainly due to the over‐firing of non‐partner cells, with a subset of neurons still functioning normally.

Based on these findings, we excluded the neurons exhibiting stress‐induced over‐firing and recalculated the excitatory‐inhibitory cycles based on normally active neurons to investigate whether inhibitory period isolated spikes retained their original information transmission function. The results showed that in the resilient group, isolated spikes during the inhibitory period were still associated with high levels of BNST‐NAc neuronal synchronization within specific time windows, whereas this was disrupted in the susceptible group (Figure 2J and Figure S2A,B). In contrast, no significant changes were observed in the center spike activity of the excitatory period for either group (Figure 2K and Figure S2C,D). Concurrently, the predictive power of NAc gamma for inhibitory period isolated spikes was significantly reduced to negligible levels, but only in the susceptible group (Figure 2L). In the LFP spectral features, we observed a reduction in BNST theta power and NAc gamma power exclusively in the depressive state, consistent with previous reports (Figure S3A–D). Furthermore, analysis of the LFP's connectivity, complexity, and aperiodic components also verified the disruption of cross‐regional signal transmission function associated with stress‐induced depression‐like behavior (Figure S4A–F).

### 2.3 DBS Restores Inhibitory Period‐Mediated Antidepressant‐Like Behavioral Effects by Suppressing Hyperactivity

Studies in both human and animal models have found that DBS targeting the BNST has potential antidepressant effects, but the underlying mechanisms at the neuronal level remain unclear. One of the direct mechanisms of DBS is the high‐frequency inhibition effect, which suppresses the excitatory electrical conduction dependent on neuronal axons. Integrating this with our findings, we hypothesized that a core mechanism of the antidepressant action of BNST‐DBS is the suppression of stress‐induced persistent over‐firing and the associated dysfunction of cross‐regional interaction in the BNST‐NAc circuit. This restores the inhibitory periods within the circuit and their associated isolated spikes, thereby producing an antidepressant effect.

**FIGURE 3:**
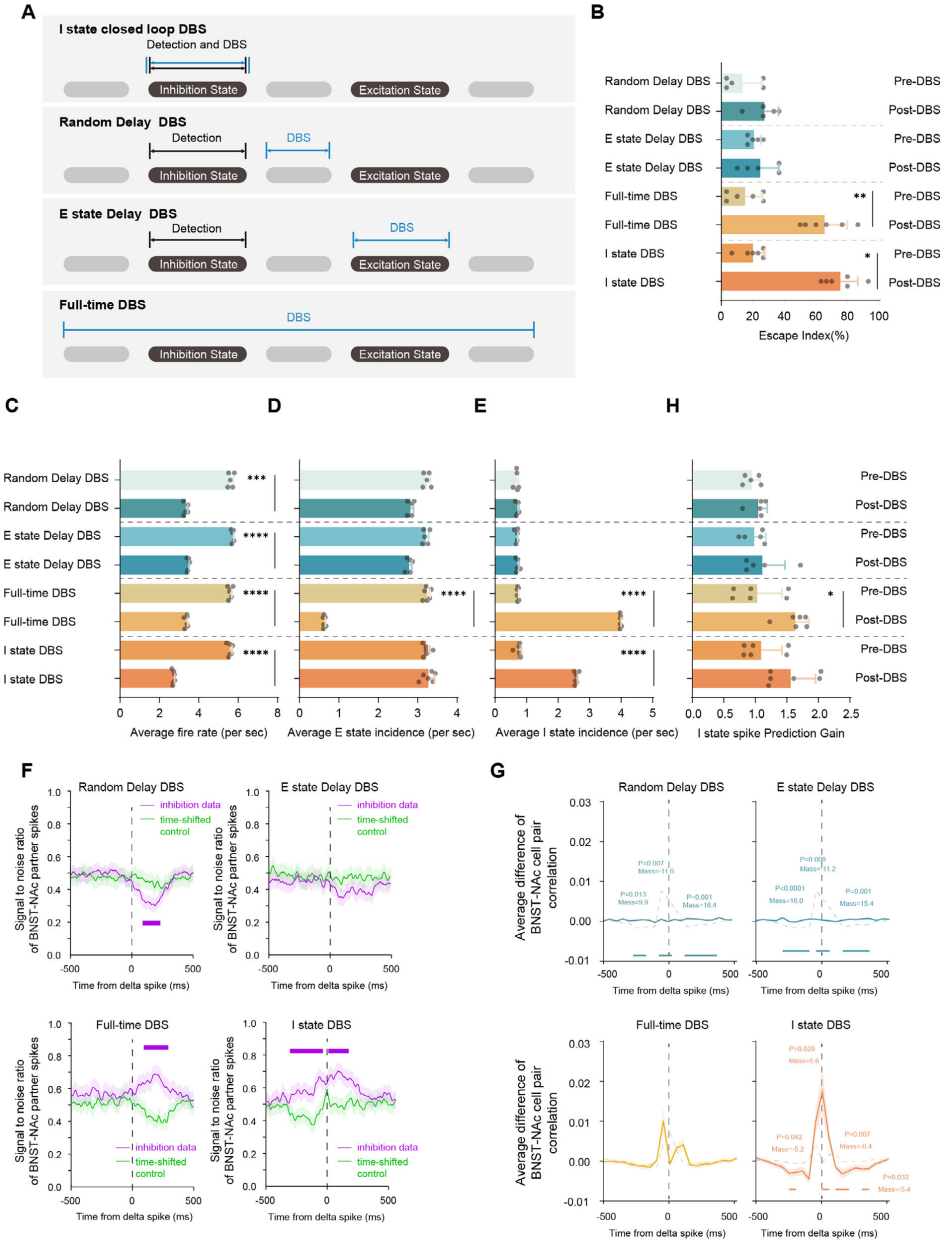
Closed‐loop DBS targeting the inhibitory period reverses stress‐induced depressive‐like behaviors by restoring inhibitory period isolated spikes in partner cells. (A) Schematic of the four DBS paradigms used in this study. (1) Inhibitory‐period‐locked closed‐loop DBS: Upon real‐time detection of an inhibitory period, a 100 ms stimulation is delivered immediately. (2) Random‐delay closed‐loop DBS: Upon detection of an inhibitory period, a 100 ms stimulation is delivered after a random 500–1000 ms delay. (3) Excitatory‐period‐locked closed‐loop DBS: Upon detection of an inhibitory period, a 100 ms stimulation is delivered at the start of the next detected excitatory period. (4) Full‐time DBS: Open‐loop continuous stimulation without spike monitoring, serving as a control. (B) Effect of all types of DBS on depressive‐like behavior (mixed‐effect analysis with Bonferroni's test for multiple comparison, *p* < 0.0001, random‐delay, pre‐DBS vs. post‐DBS, *n* = 5, p > 0.9999; excitatory‐period‐locked delay DBS, pre‐DBS vs. post‐DBS, *n* = 5, *p* > 0.9999; full‐time DBS, pre‐DBS vs. post‐DBS, *n* = 6, *p* = 0.0078; inhibitory‐period‐locked DBS, pre‐DBS vs. post‐DBS, *n* = 6, *p* = 0.0126). (C) Effect of closed‐loop DBS on BNST averaged neuron firing rate (mixed‐effect analysis with Bonferroni's test for multiple comparison, *p* < 0.0001, random‐delay, pre‐DBS vs. post‐DBS, *n* = 5, *p* = 0.0001; excitatory‐period‐locked delay DBS, pre‐DBS vs. post‐DBS, *n* = 5, *p* < 0.0001; full‐time DBS, pre‐DBS vs. post‐DBS, *n* = 6, *p* < 0.0001; inhibitory‐period‐locked DBS, pre‐DBS vs. post‐DBS, *n* = 6, *p* < 0.0001). (D) Effect of closed‐loop DBS on excitatory period‐averaged neuron firing rate (mixed‐effect analysis with Bonferroni's test for multiple comparison, *p* < 0.0001, random‐delay, pre‐DBS vs. post‐DBS, *n* = 5, *p* = 0.1344; excitatory‐period‐locked delay DBS, pre‐DBS vs. post‐DBS, *n* = 5, *p* = 0.0759; full‐time DBS, pre‐DBS vs. post‐DBS, *n* = 6, *p* < 0.0001; inhibitory‐period‐locked DBS, pre‐DBS vs. post‐DBS, *n* = 6, *p* > 0.9999). (E) Effect of closed‐loop DBS on inhibitory period averaged neuron firing rate (mixed‐effect analysis with Bonferroni's test for multiple comparison, *p* < 0.0001, random‐delay, pre‐DBS vs. post‐DBS, *n* = 5, *p* > 0.9999; excitatory‐period‐locked delay DBS, pre‐DBS vs. post‐DBS, *n* = 5, *p* > 0.9999; full‐time DBS, pre‐DBS vs. post‐DBS, *n* = 6, *p* < 0.0001; inhibitory‐period‐locked DBS, pre‐DBS vs. post‐DBS, *n* = 6, *p *< 0.0001). (F) Effect of closed‐loop DBS on the SNR around inhibitory period isolated spikes (curves and shaded areas, mean ± SEM; horizontal purple line indicates Monte Carlo test, *p* < 0.05; random‐delay DBS, up‐left, *n* = 210 events in 5 mice; excitatory‐period‐locked delay DBS, up‐right, *n* = 217 units in 5 mice; full‐time DBS, bottom‐left, *n* = 329 units in 6 mice; inhibitory‐period‐locked closed‐loop DBS, bottom‐right, *n* = 284 units in 6 mice). (G) Difference in cross‐correlation between cross‐regional firing in the BNST‐NAc circuit before and after each of the four DBS paradigms. Data are presented as group means (bold colored lines) with shaded areas indicating the Standard Error of the Mean (SEM). In each subplot, the target group is compared against the combined mean of the remaining three groups (represented by thin solid gray lines). All subplots are scaled to a uniform Y‐axis to facilitate direct comparison of effect sizes. A non‐parametric cluster‐based permutation test (1000 Monte Carlo iterations) was employed to identify significant temporal clusters while controlling multiple comparison problems. Significant clusters (α = 0.05) are marked with horizontal bars at the base of each plot and annotated with corresponding p‐values and cluster mass. (H) Effect of random‐delay closed‐loop DBS on the performance of a GLM trained to predict IPIS based on preceding NAc gamma activity (−50 ms) (mixed‐effect analysis with Bonferroni's test for multiple comparison, *p* = 0.0011, random‐delay, pre‐DBS vs. post‐DBS, *n* = 5, *p* > 0.9999; excitatory‐period‐locked delay DBS, pre‐DBS vs. post‐DBS, *n* = 5, *p* > 0.9999; full‐time DBS, pre‐DBS vs. post‐DBS, *n* = 6, *p* = 0.0345; inhibitory‐period‐locked DBS, pre‐DBS vs. post‐DBS, *n* = 6, *p* = 0.2719).

Based on this hypothesis, we designed three closed‐loop DBS paradigms based on neuronal firing frequency: inhibitory state‐locked closed‐loop DBS (I state DBS), random delay DBS (from inhibitory state), and excitatory state‐locked DBS (delayed from inhibitory state). I state DBS was defined as the synchronized initiation of a fixed‐duration DBS pulse immediately upon algorithmic detection of a designated neuron cluster entering a low‐firing period. Random delay DBS initiated the pulse after a random delay of 500–1000 ms following the detection of a low‐firing period. E state DBS did not initiate a pulse upon detection of a low‐firing period but instead waited to deliver the pulse upon the next detection of a high‐firing period. We tested our hypothesis using these three closed‐loop paradigms alongside traditional open‐loop DBS (Full‐time DBS) (Figure 3A). The results showed that only Full‐time DBS and I state DBS produced an antidepressant effect (Figure 3B). Regarding the overall firing level of the BNST, all four DBS paradigms significantly reduced the firing frequency (Figure 3C). Concerning the excitatory‐inhibitory cycles of the BNST‐NAc circuit, only Full‐time DBS significantly reduced the excitatory period firing rate, whereas both Full‐time DBS and I state DBS significantly restored the inhibitory period firing patterns (Figure 3D,E).

Regarding the function of inhibitory period‐mediated isolated spikes, Random delay DBS and E state DBS decreased the inhibitory period signal‐to‐noise ratio, while Full‐time DBS and I state DBS significantly increased it (Figure 3F). Finally, we calculated the difference in inhibitory period BNST‐NAc cell pair correlation in mice with depression‐like behavior before and after DBS to assess the impact of the four paradigms on cross‐regional signal transmission. The results indicated that I state DBS most significantly enhanced cross‐regional signal transmission in the BNST‐NAc circuit of mice with depression‐like behavior, followed by Full‐time DBS. Random delay DBS and E state DBS had no effect on the strength of cross‐regional signal transmission (Figure 3G and Figure S5A–D). Related information extracted from LFP features also supported this conclusion (Figure 3H and Figure S6A–S8D).

### 2.4 Spectral and Oscillatory Features of the Human LFP Recording Cohort with BNST‐NAc DBS for Treatment‐Resistant Depression

Leveraging a BNST‐NAc LFP clinical cohort with at least 2 years of data, we aimed to track relevant electrophysiological indicators in patients with depression. The data were collected during the open‐label phase of a study on BNST‐NAc DBS for TRD, where patients received DBS therapy and had LFP data from the BNST‐NAc circuit recorded periodically. Each recording session included two states: one LFP recording performed immediately after turning the DBS off, defined as the DBS‐on state, and another recording performed after a 120‐h washout period with the DBS off to eliminate potential stimulation‐related effects, defined as the DBS‐off state. The primary objective of this setting is to clear the short‐term, reversible electrophysiological effects associated with the DBS and stimulation itself; this duration is insufficient to eliminate deeper, persistent biological alterations, such as neuroplasticity induced by long‐term treatment or host immune responses.

This study included 18 TRD patients from whom LFP was recorded via implanted DBS electrodes (Figure S9A,B). To maintain consistency in treatment, all patients continued their medication regimens under the guidance of at least two psychiatrists throughout the study. Two branches of the bilateral multi‐contact electrodes simultaneously targeted the BNST and NAc (Figure S10A,B). Data were acquired using an Implantable Pulse Generator (IPG), which wirelessly transmitted LFP signals via Bluetooth to a computer. The primary stimulation contact was confirmed to be within the BNST, and LFP signals were recorded simultaneously from both the NAc and BNST.

**FIGURE 4:**
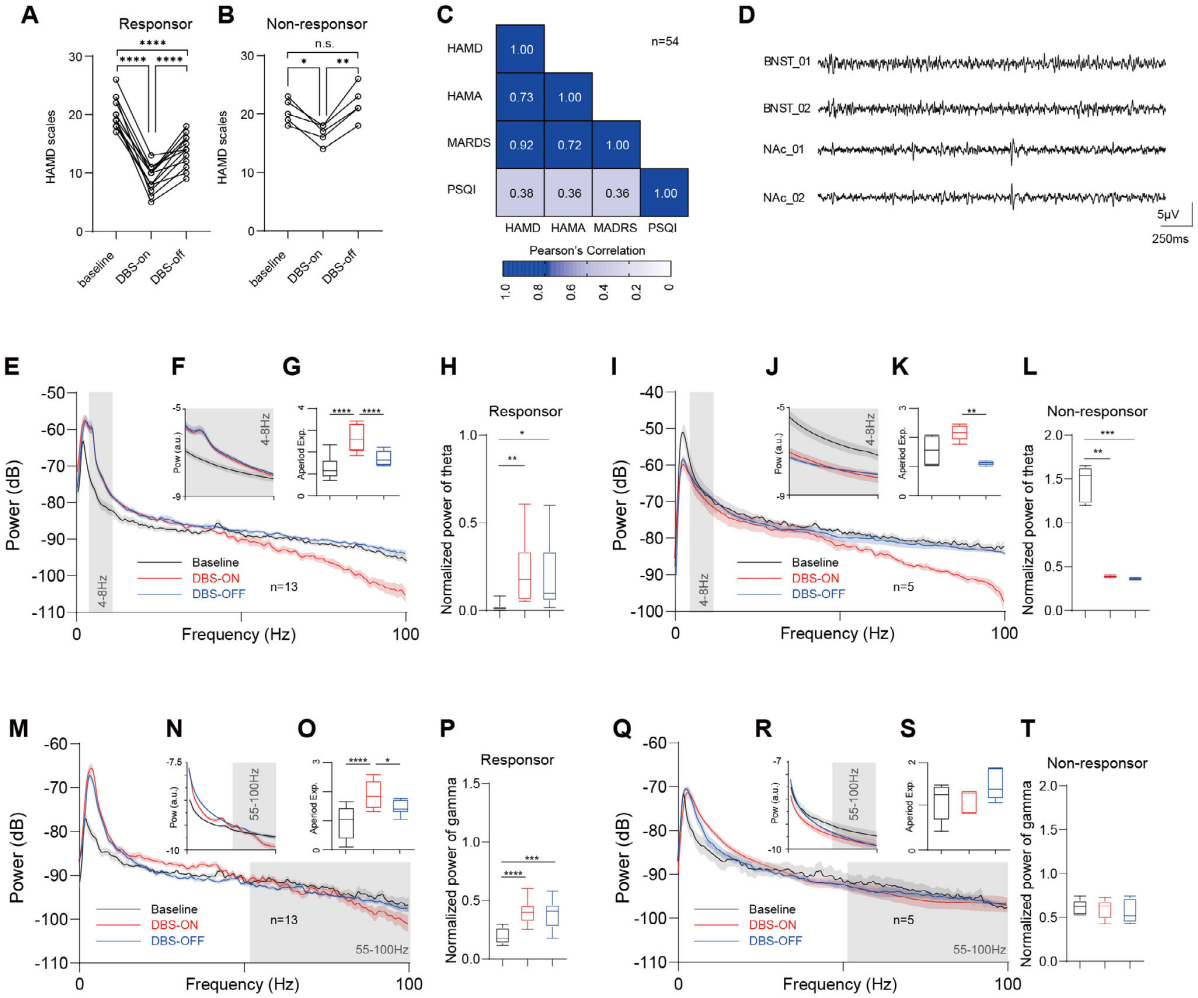
Spectral and oscillatory features of the human LFP recording cohort with BNST‐NAc DBS for TRD. (A) Clinical scales at baseline and after the 6‐month treatment of DBS, including on and off states (repeated measures one‐way ANOVA, F = 116.6, *p* < 0.0001, with Bonferroni's multiple comparison test, for baseline vs. DBS‐on, baseline vs. DBS‐off, and DBS‐on vs. DBS‐off, *p* < 0.0001). Patients with remission rates greater than 50% on the HAMD‐17 were classified in the treatment response (TR) group. For the HAMD‐17 scale, the average improvement ratio was 56.80%. (B) Clinical scales at baseline and after the 6‐month treatment of DBS, including on and off states (repeated measures one‐way ANOVA, F = 13.41, *p* = 0.0177, with Bonferroni's multiple comparison test, for baseline vs. DBS‐on *p* = 0.0195, baseline vs. DBS‐off, *p* = 0.6282, DBS‐on vs. DBS‐off, *p* = 0.0046). Patients with remission rates less than 50% on the HAMD‐17 were classified in the treatment non‐response (TN) group. For the HAMD‐17, the average improvement ratio was 18.34% (repeated measures ANOVA with Bonferroni correction: *p* = 0.0195). (C) Heat map of a matrix of Pearson's correlation coefficients between scale measures of HAMD, HAMA, MADRS, and PSQI. There is a strong consistency among the depressive symptom scales. (D) Representative traces of raw LFPs synchronously recorded in NAc and BNST (band‐pass filtered at 1–100 Hz). (E) BNST LFP in DBS responders. Average PSD at baseline (black curve, *n* = 13), in the DBS‐on state after 6 months of treatment (red curve, *n* = 13), and in the DBS‐off state (blue curve, *n* = 13). A bump in the curve near the theta band is visible, and the PSD slope is markedly different in the DBS‐on state. (F) FOOOF fit of the bump near the theta band in the LFP of DBS responders, showing clear oscillatory activity in the DBS‐on (red curve) and DBS‐off (blue curve) states after 6 months of treatment. (G) Comparison of aperiodic exponents in the LFP of DBS responders in the baseline, DBS‐on, and DBS‐off states (repeated measures ANOVA with Bonferroni correction, *n* = 13, F = 41.60, *p* < 0.0001, for baseline vs. DBS‐on, *p* < 0.0001, for baseline vs. DBS‐off, *p* = 0.0077, for DBS‐on vs. DBS‐off, *p* < 0.0001). (H) Quantitative analysis of BNST theta band power in DBS responders (repeated measures ANOVA with Bonferroni correction, *n* = 13, F = 12.19, *p* = 0.0007, for baseline vs. DBS‐on, *p* = 0.0038, for baseline vs. DBS‐off, *p* = 0.0126, for DBS‐on vs. DBS‐off, *p* > 0.9999). (I) BNST LFP in DBS nonresponders. Average PSD at baseline (black curve, *n* = 5), in the DBS‐on state after 6 months of treatment (red curve), and in the DBS‐off state (blue curve). (J) FOOOF fit of the bump near the theta band in the BNST LFP of DBS non‐responders. (K) Comparison of aperiodic exponents in the BNST LFP of DBS non‐responders in the baseline, DBS‐on, and DBS‐off states (repeated measures ANOVA with Bonferroni correction, *n* = 5, F = 16.19, *p* = 0.0049, for baseline vs. DBS‐on, *p* = 0.4082, for baseline vs. DBS‐off, *p* = 0.0948, for DBS‐on vs. DBS‐off, *p* = 0.0033). (L) Quantitative analysis of BNST theta band power in DBS non‐responders (repeated measures ANOVA with Bonferroni correction, *n* = 5, F = 134.8, *p* = 0.0003, for baseline vs. DBS‐on, *p* = 0.0010, for baseline vs. DBS‐off, *p* = 0.0009, for DBS‐on vs. DBS‐off, *p* = 0.0657). (M) NAc LFP in DBS responders. Average PSD at baseline (black curve, *n* = 13), in the DBS‐on state after 6 months of treatment (red curve, *n* = 13), and in the DBS‐off state (blue curve, *n* = 13). (N) FOOOF fit of the bump near the theta band in the NAc LFP of DBS responders. (O) Comparison of aperiodic exponents in the NAc LFP of DBS responders (repeated measures ANOVA with Bonferroni correction, *n* = 13, F = 22.25, *p* < 0.0001, for baseline vs. DBS‐on, *p* < 0.0001, for baseline vs. DBS‐off, *p* = 0.0377, for DBS‐on vs. DBS‐off, *p* = 0.0294). (P) Quantitative analysis of NAc gamma band power in DBS responders (repeated measures ANOVA with Bonferroni correction, *n* = 13, F = 27.16, *p* < 0.0001, for baseline vs. DBS‐on, *p* < 0.0001, for baseline vs. DBS‐off, *p* = 0.0007, for DBS‐on vs. DBS‐off, *p* > 0.9999). (Q) NAc LFP in DBS nonresponders. Average PSD at baseline (black curve, *n* = 5), in the DBS‐on state after 6 months of treatment (red curve, *n* = 5), and in the DBS‐off state (blue curve, *n* = 5). (R) FOOOF fit of the bump near the gamma band in the NAc LFP of DBS non‐responders. (S) Comparison of aperiodic exponents in the NAc LFP of DBS non‐responders (repeated measures ANOVA with Bonferroni correction, *n* = 5, F = 7.677, *p* = 0.0201, for baseline vs. DBS‐on, *p* > 0.9999, for baseline vs. DBS‐off, *p* = 0.1293, for DBS‐on vs. DBS‐off, *p* = 0.0561). (T) Quantitative analysis of NAc gamma band power in DBS nonresponders (repeated measures ANOVA with Bonferroni correction, *n* = 5, F = 0.1702, *p* = 0.7746, for baseline vs. DBS‐on, *p* > 0.9999, for baseline vs. DBS‐off, *p* > 0.9999, for DBS‐on vs. DBS‐off, *p* > 0.9999).

To comprehensively evaluate the effect of DBS treatment on depression‐related symptoms, we used four assessment scales (HAM‐D, HAM‐A, MADRS, and PSQI) as a baseline measurement 4 weeks after IPG implantation and assessed patients again after 6 months of continuous DBS treatment (Figure S11A,B). During the follow‐up period, we found that not all patients achieved satisfactory therapeutic outcomes. Therefore, we divided the patients into two groups based on whether their HAM‐D improvement rate exceeded 50% and further investigated the differences between them. In the treatment‐responsive TRD group, the primary outcome measure, HAM‐D, showed a significant and clinically meaningful improvement after 6 months of treatment. These improvements showed a slight rebound after turning the DBS off, but did not return to the baseline state. In contrast, in the non‐responsive TRD group, there was some improvement in HAM‐D, but it was limited to the placebo level, and scores returned to baseline after turning the DBS off (Figure 4A,B). Regarding cross‐scale assessment consistency, the results across scales for all patients showed high correlation (correlation coefficients >0.7), with only the sleep‐related PSQI showing a weaker correlation with depression and anxiety symptoms (Figure 4C).

LFP was recorded simultaneously using electrodes placed in the NAc and BNST (Figure 4D). We first attempted to identify potential characteristic signals from the Power Spectral Density (PSD). In the responsive group, two phenomena were observed in the BNST's PSD curve: the PSD curve slope (quantified as the aperiodic exponent fitted by the FOOOF algorithm) in the DBS‐on state was significantly steeper than in the Baseline and DBS‐off states (Figure 4E,F). Simultaneously, the PSD curves in the DBS‐on and DBS‐off states exhibited significantly elevated power in the 3–12 Hz theta band and a bump in the curve suggesting oscillatory activity, as identified by the FOOOF algorithm (Figure 4G,H). In the nonresponsive group, only the significantly steeper PSD curve slope in the DBS‐on state was observed (Figure 4I–L). Therefore, we concluded that the change in the PSD curve slope, reflecting enhanced inhibitory activity in the neuronal cluster, is likely a direct local effect of DBS on the nucleus, whereas the theta activity may be related to the antidepressant effect of DBS (Figure S12A).

The NAc acts as a downstream nucleus to the stimulated BNST. In the responsive group, the PSD curve slope in the DBS‐on state also became steeper, though the magnitude of the change was less pronounced than in the BNST (Figure 4M,N). Oscillation analysis using the FOOOF algorithm indicated the emergence of clear gamma oscillations and enhanced band power in both the DBS‐on and DBS‐off states post‐treatment (Figure 4O,P, Figure S12B and S13D). Neither of these phenomena was observed in the non‐responsive group (Figure 4Q–T).

It is known that DBS exerts a high‐frequency inhibition effect on the neuronal firing at the direct stimulation site, regardless of whether a therapeutic effect is produced. Effective DBS, however, mediated the generation of local theta oscillations in the BNST, the power of which was significantly correlated with symptom improvement; these theta oscillations partially arise from the excitatory‐inhibitory balance of the neuronal population. In the downstream NAc, effective DBS induced gamma oscillations, which are primarily mediated by inhibitory interneurons. Notably, an inhibitory effect on NAc neurons was observed only in the responsive group. This could be because DBS restored the inhibitory GABAergic output from the BNST to the NAc in responders, whereas this inhibitory output remained dysfunctional in non‐responders. Therefore, integrating these findings with the results from our animal experiments, we hypothesize that one of the core mechanisms by which DBS exerts its therapeutic effect at the neuronal level is by suppressing abnormal neuronal firing, restoring excitatory‐inhibitory cycles and related potential activities, and thereby re‐establishing the internal regulatory mechanisms of the circuit.

### 2.5 Features of Circuit Regulation Mechanisms in the Human LFP Recording Cohort with BNST‐NAc DBS for TRD

To test the above hypothesis, we planned a reverse validation approach: first, we explored the effect of DBS on circuit regulation mechanisms; second, we investigated whether the restoration of these mechanisms depends on excitatory‐inhibitory cycles and LFP activity; and finally, we inferred the role of abnormal neuronal firing in this process.

**FIGURE 5:**
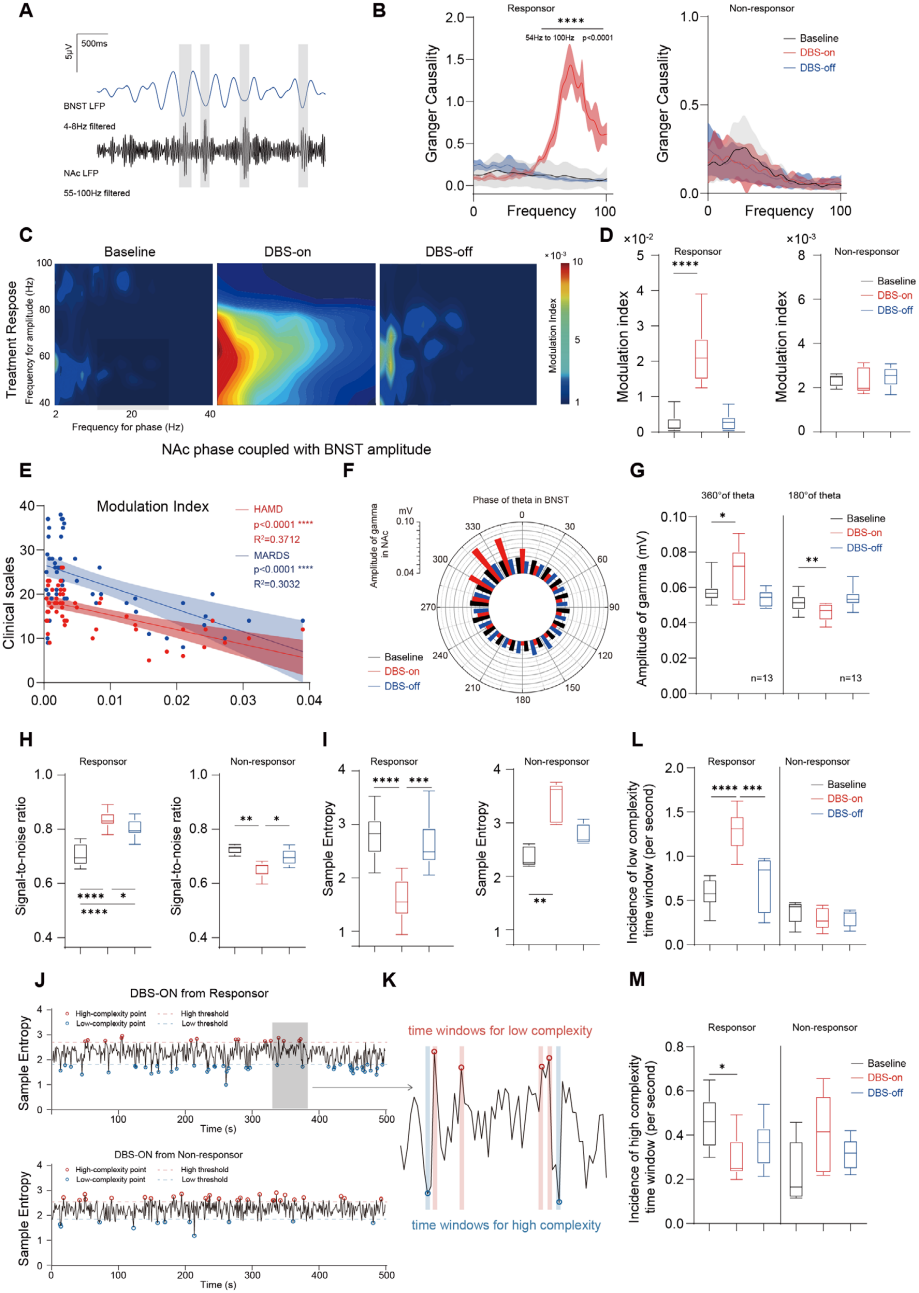
Functional connectivity features of the human LFP recording cohort with BNST‐NAc DBS for TRD. (A) Schematic representation of theta and high‐gamma phase‐amplitude coupling in the BNST‐NAc circuit. Gray shadows indicate coupled oscillations. (B) Pair‐wise conditional multivariate Granger causality (MVGC) from BNST to NAc local field potential signals (Monte Carlo test, the shadowed area indicates standard error, TR group, left, *p* < 0.0001 from 54 to 100 Hz;TN group, right) (C) Representative PAC heatmap observed at three states (left: baseline, middle: DBS‐on, right: DBS‐off). In the TR group, PAC was only enhanced for the DBS‐on state. (D) PAC modulation index at baseline, DBS‐on, and DBS‐off status in the (repeated measures one‐way ANOVA with Bonferroni correction, TR group, left, *n* = 13, *p* < 0.0001, F = 71.83; *p* < 0.0001 for baseline vs. DBS‐on; *p* = 0.9529 for baseline vs. DBS‐off; TN group, right, *n* = 5, *p* = 0.6868, F = 0.1943; *p* > 0.9999 for baseline vs. DBS‐on; *p* = 0.9761 for baseline vs. DBS‐off; *p* > 0.9999 for DBS‐on vs. DBS‐off). (E) Regression analysis showing the significant relationship between PAC‐MI and depressive symptoms, including the HAM‐D scales (red, R^2^ = 0.3736, *p* < 0.0001, 95% CI [−0.7554, −0.4107]) and MADRS scales (blue, R^2^ = 0.3032, *p* < 0.0001, 95% CI [−0.7133, −0.3318]). The shadowed area indicates a 95% confidence interval. (F) Group‐averaged circular NAc high‐gamma amplitude distribution by the theta phase of BNST. High‐gamma oscillation had a higher amplitude at ∼360° of the theta phase. (G) High‐gamma amplitude comparison at 360° and 180° (±10° of phase width). DBS‐on showed a significantly higher amplitude at 360° and a significantly lower amplitude at 180°. (Repeated measure one‐way ANOVA with Bonferroni correction, TR group, *n* = 13, *p* = 0.0031, F = 10.28 for comparison of 360°; *p* = 0.0228 for baseline vs. DBS‐on; *p* = 0.1515 for baseline vs. DBS‐off. For comparison of 180°, *n* = 13, *p* = 0.0005, F = 12.23, *p* = 0.0027 for baseline vs. DBS‐on; *p* = 0.2472 for baseline vs. DBS‐off). (H) Signal‐to‐noise ratio at baseline, DBS‐on, and DBS‐off status in the TR group(left, repeated measures ANOVA with Bonferroni correction, *n* = 13, F = 54.05, *p* < 0.0001, for baseline vs. DBS‐on, *p* < 0.0001, for baseline vs. DBS‐off, *p* < 0.0001, for DBS‐on vs. DBS‐off, *p* = 0.0206) and TN group (right, repeated measures ANOVA with Bonferroni correction, *n* = 5, F = 21.25, *p* = 0.0013, for baseline vs. DBS‐on, *p* = 0.0036, for baseline vs. DBS‐off, *p* = 0.2478, for DBS‐on vs. DBS‐off, *p* = 0.0633). Signal‐to‐noise ratio was calculated as the ratio of filtered BNST‐LFP at 1–100 Hz and 1–160 Hz. Considering that the stimulus frequency was 160 Hz, the latter signal may involve stimulus‐induced electrophysiological perturbations. The perturbation is significantly stronger in the TN group. (I) Sample entropy at baseline, DBS‐on, and DBS‐off status in the TR group(left, repeated measures ANOVA with Bonferroni correction, *n* = 13, F = 27.89, *p* < 0.0001, for baseline vs. DBS‐on, *p* < 0.0001, for baseline vs. DBS‐off, *p* = 0.9809, for DBS‐on vs. DBS‐off, *p* = 0.0003) and TN group (right, repeated measures ANOVA with Bonferroni correction, *n* = 5, F = 18.55, *p* = 0.0034, for baseline vs. DBS‐on, *p* = 0.0041, for baseline vs. DBS‐off, *p* = 0.1849, for DBS‐on vs. DBS‐off, *p* = 0.1281). The TR group had significantly lower complexity after DBS treatment. The TN group had significantly higher complexity in DBS‐on status. (J) Representative sample entropy sequences calculated in 1‐second time windows. The criteria for defining extreme values are mean ± 2 SD. High‐complexity windows are marked by red points, and low‐complexity windows are marked by blue points. The TR group has more low‐complexity points. (K) Schematic diagram of the assumption of effective DBS‐induced connectivity‐enhancing time windows. The red shadowed area indicates high complexity accompanied by disturbance. The blue shadowed area indicates low complexity accompanied by connectivity. (L) Incidence of low‐entropy time windows in TR group (left, *n* = 13, *p* < 0.0001, F = 33.24; *p* < 0.0001 for baseline vs. DBS‐on; *p* = 0.4256 for baseline vs. DBS‐off; *p* = 0.0002 for DBS‐on vs. DBS‐off) and TN group (right, *n* = 5, *p* = 0.5210, F = 0.6327; *p* = 0.4319 for baseline vs. DBS‐on; *p* = 0.7561 for baseline vs. DBS‐off; *p* = 0.9923 for DBS‐on vs. DBS‐off) at baseline, DBS‐on, and DBS‐off status. (M) Incidence of high‐entropy time windows in TR group (left, *n* = 13, *p* = 0.0087, F = 6.038; *p* = 0.0238 for baseline vs. DBS‐on; *p* = 0.0875 for baseline vs. DBS‐off; *p* = 0.4165 for DBS‐on vs. DBS‐off) and the TN group (right, *n* = 5, *p* = 0.1665, F = 2.385; *p* = 0.2724 for baseline vs. DBS‐on; *p* = 0.5378 for baseline vs. DBS‐off; *p* = 0.4600 for DBS‐on vs. DBS‐off) at baseline, DBS‐on, and DBS‐off status.

First, based on LFP recorded simultaneously after 6 months of treatment, frequency domain Granger causality analysis was used to detect connectivity changes induced by DBS. The results showed that DBS originating from the BNST could predict the information flow to the NAc from 54 Hz to over 100 Hz, a phenomenon observed only in the DBS‐on state of the responsive group (Figure 5A–C). This implies that therapeutic DBS can effectively induce an enhancement of information flow from the BNST to the NAc, which is in preliminary agreement with the conclusions inferred from the PSD analysis. Further exploration of the specific pattern of this enhanced information flow was needed.

The phase‐amplitude coupling (PAC) results for the two cross‐regional oscillatory activities enhanced in the spectral analysis showed that the coupling between the BNST theta phase and NAc gamma amplitude was enhanced in the DBS‐on state. The PAC modulation index (PAC‐MI) was also significantly elevated, a phenomenon observed only in the responsive group (Figure 5D and Figure S14A). We examined the correlation between clinical scales and the PAC‐MI in different states using Pearson's correlation coefficient. Linear regression analysis revealed that the PAC‐MI was significantly correlated with clinical scales (Figure 5E). Furthermore, the phase‐amplitude distribution showed that the amplitude of gamma activity peaked at approximately 330° of the low‐frequency activity's phase, while being significantly lower at approximately 180° of the theta phases (Figure 5F,G).

These results indicate that therapeutic DBS effectively restored the BNST‐NAc circuit regulation mechanism mediated by periodic LFP activity. Next, it was necessary to explore whether the restoration of this mechanism depends on excitatory‐inhibitory cycles. Signal‐to‐noise ratio (SNR) and sample entropy are considered important indicators of the complexity of local information in neuronal activity. Therefore, we first explored the overall changes in these metrics. In the responsive group, the SNR of the BNST field potential increased, and sample entropy decreased during the DBS‐on phase. This reflects a reduction in the complexity and disorder of signals within the nucleus, suggesting that stimulation increased the activity coherence of the neuronal cluster. In contrast, the responses of SNR and sample entropy in the non‐responsive group were completely opposite (Figure 5H–K).

We hypothesized that periods of lower local field potential complexity might be associated with stronger inter‐regional connectivity. Therefore, we divided the recorded LFP signals into continuous 1‐s time windows and calculated the sample entropy for all time windows across the entire signal (Figure 5L). Time windows with sample entropy exceeding two standard deviations from the mean were defined as high‐ and low‐complexity points (Figure 5M). The results showed that during DBS treatment, the responsive group had an increase in low‐complexity time windows and a decrease in high‐complexity time windows. In contrast, the non‐responsive group showed no change in low‐complexity windows but an increase in high‐complexity windows. This suggests that the DBS‐induced enhancement of information flow may depend on specific time windows of local neuronal information complexity.

**FIGURE 6:**
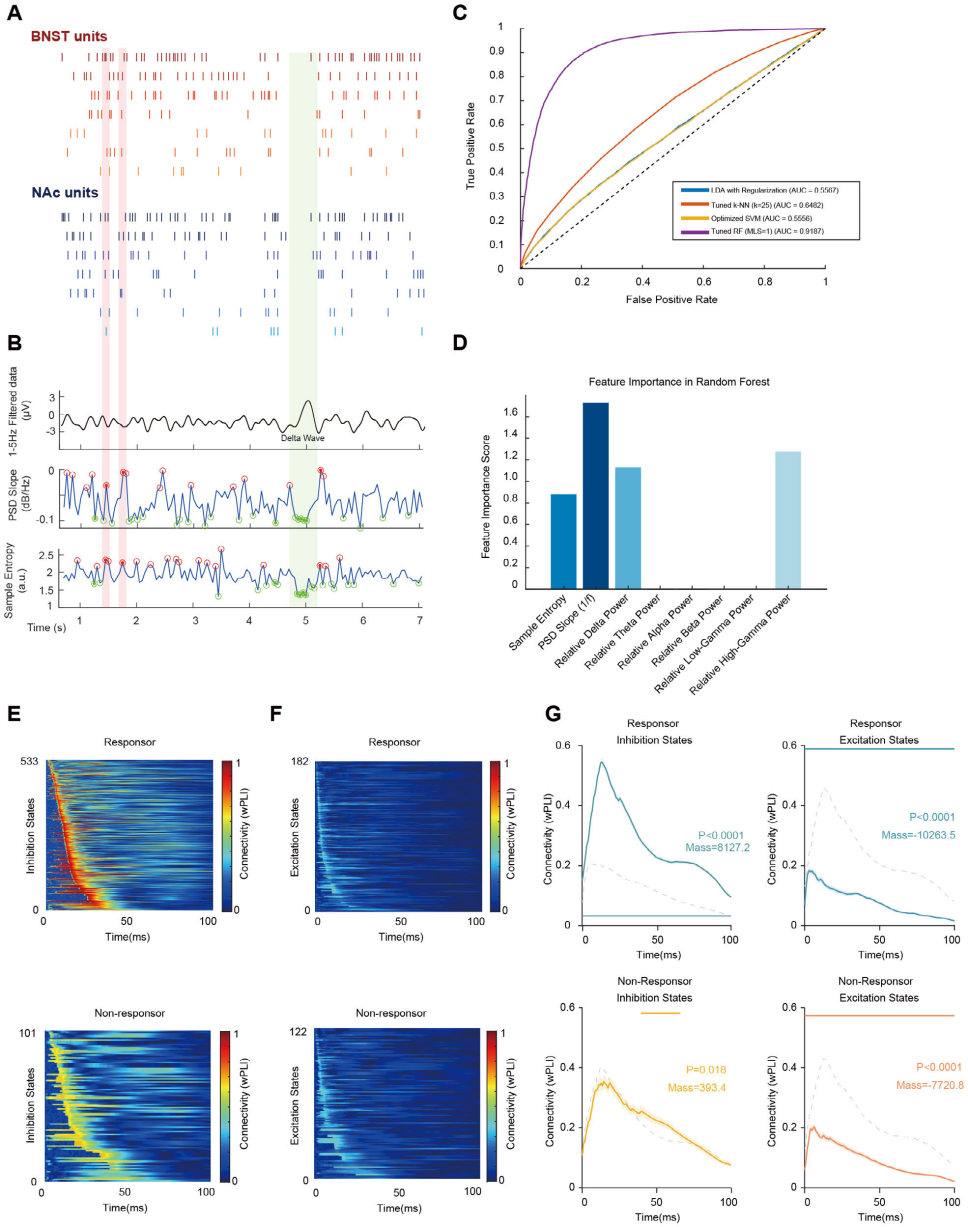
BNST‐NAc DBS treats TRD by restoring inhibitory period‐mediated cross‐regional signal transmission. (A) Cross‐species transfer of algorithm‐driven features from neuronal firing data. A hypothesis for an algorithm that reflects spike firing states using LFP features is shown, based on simultaneously recorded BNST‐NAc neuronal firing in an animal (7 BNST neurons, 7 NAc neurons). (B) Simultaneously recorded BNST LFP features corresponding to (A). (Top) Raw data band‐pass filtered for delta (1–5 Hz), with clear delta waves visible. (Middle) BNST LFP PSD slope calculated in 50 ms sliding windows, with maxima and minima marked by hollow circles. (Bottom) LFP sample entropy calculated in 50 ms sliding windows, with maxima and minima marked by hollow circles. Points that simultaneously satisfy the criteria of being covered by a delta wave, having a very low PSD slope, and having very low sample entropy are presumed to be inhibitory periods. Conversely, points that are >500 ms from a delta wave, have a very high PSD slope, and very high sample entropy are presumed to be excitatory periods. (C) Performance comparison of four tuned classification algorithms in the task of distinguishing neuronal E/I states. This figure shows the Receiver Operating Characteristic (ROC) curves on the test set for Regularized Linear Discriminant Analysis (LDA), Tuned k‐Nearest Neighbors (k‐NN), Optimized Support Vector Machine (SVM), and Tuned Random Forest (RF). The x‐axis is the False Positive Rate (FPR), and the y‐axis is the True Positive Rate (TPR). The Area Under the Curve (AUC) for each model is shown in the legend. A higher AUC indicates better classification performance. The dashed diagonal line (AUC = 0.5) represents random chance. The Tuned RF (MLS = 1) model showed the highest performance (AUC = 0.9187). (D) Feature importance from the Random Forest model: sample entropy (1.7283), PSD slope (0.8816), relative delta power (1.1291), relative gamma power (1.2763). (E) Heatmap showed population peri‐entropy time histograms sorted by wPLI peak time for inhibition states in both groups (top: TR, 533/715; bottom: TN, 101/223). (F) Heatmap showed population peri‐entropy time histograms sorted by wPLI peak time for excitation in both groups (top: TR, 182/715; bottom: TN, 122/223). (G) Population‐averaged peri‐entropy time histograms for differentiated complexity states. Waveforms are presented as group means (bold colored lines) ±Standard Error of the Mean (SEM, shaded areas). In each panel, the target group is compared against the aggregated mean of the remaining three groups (represented by gray lines). A non‐parametric cluster‐based permutation test (1‐vs.‐others) was performed to identify significant temporal clusters while correcting for multiple comparisons. Significant clusters (*p* < 0.05) are marked with horizontal bars at the base of each plot and annotated with corresponding cluster‐corrected p‐values.

To confirm the relationship between the DBS‐induced restoration of regulatory mechanisms and the excitatory‐inhibitory cycles of neuronal firing, further exploration was necessary. However, the excitatory‐inhibitory state inferred from conventional LFP parameters is indirect and lacks quantitative evidence. Based on previous research, it is presumed that during the excitatory period, high‐frequency activity (>1000 Hz) of neuronal spike potentials increases, and its main effects on the field potential include: a PSD slope approaching 0, increased sample entropy, and incompatibility with the DOWN‐STATE reflected by delta oscillations. Considering that the microscale neuronal composition of the BNST‐NAc in mice is similar to that in humans, we believed it was possible to define excitatory and inhibitory periods in human LFP using a cross‐species algorithm. Therefore, we first defined excitatory and inhibitory periods based on firing frequency from BNST‐NAc spike raster plots recorded in mice. We then assigned labels to synchronized sliding time windows of the LFP and extracted the PSD slope, sample entropy, and spectral power from the LFP data (Figure 6A,B).

We attempted to discriminate neuronal firing states from LFP features using several linear‐based machine learning algorithms, including Linear Discriminant Analysis (LDA), k‐Nearest Neighbors (k‐NN), Support Vector Machine (SVM), and Random Forest. Among the classifiers evaluated, the hyperparameter‐tuned Random Forest model (minimum leaf size = 1) demonstrated the highest performance. On an independent test set, the model achieved an overall classification accuracy of 84.01%. The model exhibited excellent discriminative ability, as indicated by an Area Under the Curve (AUC) of the Receiver Operating Characteristic (ROC) curve of 0.9187.

Regarding class‐specific performance for “UP states,” the model yielded a precision of 85.93%, a recall of 81.42%, and an F1‐Score of 0.8362. Furthermore, the specificity for correctly identifying “DOWN states” was 86.60%. These results collectively indicate that the LFP feature‐based Random Forest model can effectively and accurately classify distinct neuronal firing states (Figure 6C). To determine which LFP features contributed most to the classification, we conducted a feature importance analysis on the best‐performing Random Forest model (Figure 6D). The 1/f aperiodic slope and sample entropy (SampEn) of the LFP signal were the important features for distinguishing between UP and DOWN states. In spectral features, the relative power of the delta and high Gamma (60–100 Hz) bands also contributed to the classification. This indicates that the high‐frequency, high‐complexity activity associated with the UP state, along with changes in the overall morphology of the signal spectrum, are key bases for the classification decision (Figure 6D). A random forest classifier was chosen to account for the inherent complexity of LFP signals. Multiple features, including signal complexity, aperiodic activity, and spectral power, contribute non‐linearly and interactively to the classification—a complexity that cannot be captured by linear models.

Using this algorithm, which was trained on neuronal‐level data from a cross‐species model, we defined the inhibitory and excitatory periods in the TRD LFP cohort data. We then calculated the connectivity of the BNST‐NAc circuit (measured by wPLI) and generated a Peri‐Event Time Histogram (PETH). For the PETH, time windows were sorted according to the order in which their wPLI reached its peak (Figure 6E–H). The results showed that there were more inhibitory periods in the responsive group than in the non‐responsive group (533/715 = 74.54% vs. 101/223 = 45.29%). Furthermore, both groups exhibited similar connectivity results during the excitatory period, but the connectivity of the responsive group during the inhibitory period was significantly higher than that of the nonresponsive group (Figure 6G–I).

### 2.6 Two‐Year BNST‐NAc LFP Clinical Cohort Validates that DBS Restores Inhibitory Period‐Mediated Cross‐Regional Signal Transmission

**FIGURE 7:**
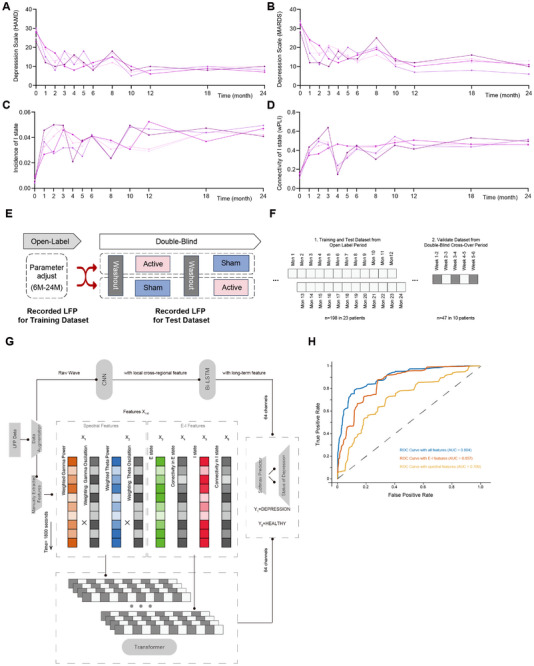
Validation of DBS restoration of inhibitory period‐mediated cross‐regional signal transmission in the BNST‐NAc LFP clinical cohort. (A) Changes in depression scores (HAMD) for 5 patients from the BNST‐NAc LFP clinical cohort over a 24‐month follow‐up period. (B) Changes in depression scores (MADRS) for 5 patients from the BNST‐NAc LFP clinical cohort over a 24‐month follow‐up period. (C) Changes in the incidence of intra‐circuit I states extracted from the 5 patient's LFP over the 24‐month follow‐up. (D) Changes in the connectivity of intra‐circuit I states extracted from the 5 patient's LFP over the 24‐month follow‐up. (E) Flowchart of the study design, moving from the open‐label phase to the randomized, double‐blind, crossover clinical trial. (F) Construction and partitioning of the dataset for the algorithm. 198 states from 23 patients in the open‐label follow‐up, including depression scores and BNST‐NAc LFP, were partitioned into training and test sets. 47 states from 10 patients in the crossover trial were used as the validation set. (G) Design flowchart of the Transformer algorithm. (H) Feature importance analysis using a CNN‐BiLSTM‐Transformer architecture was conducted to investigate the utility of E–I features for predicting depressive states. According to the comparison of depression state prediction performance across various algorithmic input signals, E–I features provided a more substantial contribution to overall prediction accuracy than spectral features.

To further validate the robustness of the relevant indicators in the pathological process of depressive disorder, we conducted follow‐ups monthly for the first 6 months after DBS treatment, every 2 months from 6 months to 1 year, and every 6 months from one to 2 years. 5 patients completed the entire 2‐year follow‐up protocol. The study tracked changes in clinical scales for depressive disorder, including HAMD and MADRS (Figure 7A,B), the power of key frequency bands in the BNST and NAc (Figure S15A,B), the incidence of algorithm‐defined excitatory and inhibitory periods in the BNST‐NAc circuit (Figure 7C and Figure S15C), and the functional connectivity strength (wPLI) of the BNST‐NAc circuit during these excitatory and inhibitory periods (Figure 7D and Figure S15D). A multiple linear regression analysis between the six aforementioned electrophysiological indicators and clinical outcomes revealed a strong consistency between the regulatory function of the BNST‐NAc circuit and clinical symptoms, as suggested by the cohort's follow‐up data (Figure S16A–G).

The latter part of this clinical study entered a double‐blind crossover RCT phase involving 10 patients (Figure 7E illustrated study design, including open‐label phase and blind phase). This phase was designed to provide clinical evidence for the efficacy and safety of DBS therapy for TRD and yielded statistically significant clinical results. Details regarding the design of the randomized double‐blind trial and its primary endpoint results can be found in Figure S17. Specifically, 18 patients completed the RCT phase. Active DBS resulted in a mean HAMD score of 11.1 ± 6.6, compared to 20.5 ± 6.4 during the sham phase (*p* < 0.001), confirming the robust antidepressant efficacy of the intervention. During the RCT, 10 patients had their clinical symptoms and LFP data recorded across 5 periods. We therefore used the 198 data segments (1800 s each) collected from the 23 patients during the initial open‐label phase as the training and test sets. The 47 data segments (1800 s each) collected during the RCT phase were used as a heterogeneous validation set (Figure 7F). A deep learning framework was implemented not primarily for clinical prediction, but as a robust platform to perform ablation analyses, thereby quantifying the relative importance of different electrophysiological features in capturing the neural signatures of depression.

In our preprocessing pipeline, we defined the following features from the LFP: excitatory periods, inhibitory periods, BNST‐NAc connectivity during excitatory periods, BNST‐NAc connectivity during inhibitory periods, as well as spectral and oscillatory features. These were combined with the raw waveform as input to the algorithm. To fuse these multi‐faceted data features, after a 10‐fold data augmentation, the raw waveform was processed using a CNN to extract local features from short time windows of the BNST‐NAc dual‐channel data, and a Bi‐LSTM was used to extract long‐term temporal features from individual brain regions. The hand‐crafted spectral and E‐I features were fed into a Transformer based on an attention mechanism for feature analysis. This information was then concatenated and fused with the output from the CNN‐Bi‐LSTM, and the final depressive state judgment was output through a softmax layer (Figure 7G and Figure S17A).

To systematically parse the contribution of specific biomarkers, we conducted an ablation study by systematically varying the input configurations of the deep learning module. This allowed us to isolate and compare the diagnostic value of conventional spectral features against our hypothesized E‐I cycle biomarkers. The classification performance was worst when only spectral features were input. Performance was already quite good when only E‐I features were input. While the model using only spectral features performed poorly, the integration of E‐I features led to a dramatic and significant increase in decoding accuracy on the validation set. This ablation‐based feature importance analysis underscores that E‐I cycle dynamics provide the decisive information for identifying the depressive state, validating E‐I balance as an important pathological process (Figure 7H).

## 3 Discussions

This study centered on the core pathophysiology of treatment‐resistant depression (TRD) and its deep‐brain stimulation (DBS) treatment mechanisms. Through a cross‐species, multi‐level electrophysiological investigation, we conducted an in‐depth exploration of the dysfunction within the Bed Nucleus of the Stria Terminalis (BNST)‐Nucleus Accumbens (NAc) neural circuit. Our core findings reveal for the first time that, in a depressive state, the pathological hyperactivity of BNST neurons impairs the capacity for high‐fidelity signal transmission to the NAc by disrupting the critical communication pattern of inhibitory period isolated spikes (IPIS). We further confirmed that the antidepressant efficacy of DBS does not originate from simple neural suppression. Instead, it is achieved by precisely restoring network inhibitory periods and re‐establishing efficient IPIS‐mediated signal transmission. These findings not only provide direct electrophysiological evidence for the pathophysiological hypothesis that BNST hyperactivity leads to impaired NAc function but also establish a solid biological foundation for understanding the mechanisms of DBS action and advancing toward precision neuromodulation therapy.

The BNST‐NAc neural circuit connects the brain's stress‐aversive and reward‐motivation systems, and its dysfunction is considered a key neural basis for the core symptoms of depression. This study first confirmed the existence of IPIS as a high signal‐to‐noise ratio (SNR) communication code within this circuit in healthy mouse models, particularly during the inhibitory periods of delta waves. The gamma power in the NAc significantly predicted the IPIS of partner BNST neurons, indicating that IPIS is a nonrandom, high‐fidelity communication pattern regulated by specific network states, which lays the groundwork for functional information transmission in this circuit [41]. Building on this, we used the learned helplessness model to directly verify how chronic stress disrupts this precise mechanism. The study found that stress‐susceptible mice exhibited persistent hyperactivity of BNST neurons, which is consistent with multiple previous findings in emotional circuits [9, 42, 43]. We discovered that this phenomenon also occurred during critical network inhibitory periods, leading directly to the breakdown of the IPIS pattern and a decrease in BNST‐NAc neuronal synchrony. In stark contrast, stress‐resilient mice maintained their IPIS‐mediated communication function despite undergoing the same stress protocol. This finding suggests that the robustness of IPIS communication in the BNST‐NAc circuit may be a key foundation for neural resilience against depression, while its disruption is a core pathophysiological element of the disorder and a direct manifestation of how overactive BNST signals pathologically inhibit NAc reward and motivation functions.

Findings in the BNST‐NAc circuit are parallel to isolated cortical computations described in HPC‐PFC interaction. This suggests a global computational principle for high‐fidelity communication in the brain [44, 45]. By using signal processing algorithms similar to those in cortical studies, we show that IPIS in the BNST‐NAc circuit are mathematically isomorphic to cortical delta spikes. We propose that coupling sparse, information‐bearing spikes with the global inhibitory phase of the network to generate transient high SNR windows is a conserved strategy. This strategy allows neural circuits to protect critical information flow from background noise. Depression leads to the failure of this mechanism. Stress‐induced hyperactivity functions as persistent noise that fills these silent windows and disrupts the ability of the circuit to distinguish signal from noise. DBS exerts its therapeutic effect by restoring this fundamental SNR mechanism.

Traditional views suggest that DBS acts through local inhibition or information masking, but its specific regulatory mechanisms at the circuit level remain unclear [46]. This study conducted an in‐depth investigation of the effects of DBS on the BNST‐NAc circuit based on the theory of its dual action on cell bodies and axons [47]. Our closed‐loop DBS experiment results provide more refined evidence for the antidepressant mechanisms of DBS. The research indicates that not all DBS paradigms are effective. Only continuous DBS and closed‐loop DBS precisely locked to the inhibitory state (I‐state DBS) produced significant antidepressant effects. The common feature of these two effective paradigms is that they both significantly suppressed the pathological over‐firing of BNST neurons and restored network inhibitory periods, which in turn enhanced the SNR of IPIS and the efficiency of cross‐regional signal transmission. Notably, I‐state DBS was most effective in restoring cross‐regional communication. It strongly corroborates our central hypothesis that the therapeutic effect of DBS hinges on the precise repair of network inhibitory periods that are compromised in a pathological state, rather than on a general suppression of neural activity [48].

In this study, the BNST‐NAc circuit is defined as a highly specialized GABAergic projection from somatostatin‐positive (SOM+) neurons in the anterodorsal BNST (adBNST) that precisely targets parvalbumin‐positive (PV+) interneurons in the Nucleus Accumbens Shell (sNAc) [49]. This pathway constitutes an inhibitory‐inhibitory series structure, where SOM+ neurons in the BNST exert an inhibitory effect on PV+ neurons in the NAc. Based on this, the therapeutic effect of DBS in this study does not arise from global excitation or inhibition of the NAc region but is achieved through a more subtle microcircuit regulation mechanism. DBS may enhance GABA release by activating the axon terminals of adBNST SOM+ neurons projecting to the sNAc, thereby restoring effective inhibitory control over hyperexcitable PV+ neurons [50].

This study proposes the postinhibitory rebound (PIR) mechanism as the cellular basis for the observed IPIS pattern. PIR is an active computational process driven by both HCN and T‐type calcium channels [51]. The process achieves sparse coding with high SNR and precise timing through a temporal logic where firing follows inhibition within a background of global network inhibition [52]. PIR utilizes membrane potential reset to eliminate background interference and ensures signal saliency during complex computations [53]. Given that BNST projection neurons exhibit significant rebound firing characteristics, the IPIS pattern constitutes the primary architecture for transmitting information to the NAc [54]. However, in a depressive state, pathological hyperactivity in the BNST interrupts the inhibitory phase and leads to the failure of circuit communication. After defining PIR as an active computational process at the cellular level, we proposed its temporal logic at the microcircuit level. Perisomatic inhibition of downstream principal neurons by PV+ interneurons provides the required hyperpolarization reset signal for rebound firing.

Inhibitory period isolated spikes (IPIS) are a specific neuronal firing pattern characterized by the appearance of a single action potential within a significant inhibitory period. In neural circuits, PV+ interneurons, particularly basket cells, exert powerful perisomatic inhibition on nearby neurons [55]. We interpret the DBS‐induced IPIS patterns observed in our study as direct electrophysiological evidence that PV+ neuron activity is being effectively modulated. The proposed causal chain is as follows. DBS enhances the inhibition from BNST‐SOM+ neurons onto NAc‐PV+ neurons, leading to a brief but potent suppression of these hyperexcitable PV+ neurons. When the perisomatic inhibition provided by the PV+ neurons is temporarily lifted, their downstream NAc principal neurons are able to fire within this precise and brief time window, thus forming the IPIS pattern.

The oscillatory activity is one of the foundational findings of this study. Neural oscillations are primarily generated by inhibitory interneurons [56]. In particular, fast‐spiking PV‐positive interneurons are considered key to generating gamma band (30‐80 Hz) oscillations through their extensive mutual inhibitory connections (I‐I networks) [57]. The fast‐acting GABAA‐mediated inhibition released by PV cells provides precise temporal windows for network activity, causing the firing of principal cell populations to exhibit a gamma‐frequency rhythm [58]. The pathological oscillation model provides a powerful explanatory framework for stress‐induced affective disorders, positing that disease symptoms arise from abnormal synchronous activity in specific neural circuits [59]. In depression, growing evidence indicates that connectivity and oscillation patterns in cortico‐limbic circuits, including the anterior cingulate cortex, amygdala, and prefrontal cortex, are altered, showing changes in theta and gamma oscillatory activity. This is supported by early animal studies [9, 60]. Gamma oscillation in emotional circuits has been identified as a potential real‐time biomarker for depression and obsessive‐compulsive disorder (OCD), which could be used to guide closed‐loop DBS therapy [61]. Regarding the clinical significance of BNST theta activity, it is important to clarify that it could be viewed as a general electrophysiological response of specific neural circuits to DBS stimulation instead of a pathological marker of depression. This nonspecific characteristic has been validated in research on other psychiatric disorders. In DBS treatments for OCD and PTSD, theta oscillation enhancement related to clinical efficacy has been observed. Therefore, theta activity may reflect a shared response mechanism of local neurons to electrical stimulation and represent the fundamental physical properties of DBS in modulating circuit functions [62, 63].

In Figure 1L, we observed the gamma power in the NAc decreased closing to the delta wave troughs. This rapid decline reflects the intrinsic physiological process of the circuit entering a hyperpolarized inhibitory period. Gamma oscillations depend on local network firing coordinated by PV+ interneurons. During delta waves, the excitatory inputs that drive E‐I circuits are reduced, which leads to a temporary decrease in the oscillation mechanism. Valero et al. found that interneurons active during the inhibitory period produce tonic inhibition, which likely contributes to the gamma reduction [64]. Research by Zucca et al. showed that the thalamus during the inhibitory period deepens cortical hyperpolarization via feedforward inhibition [65]. This process suppresses the gamma oscillations generated by PV+ neurons. The gamma decrease in the BNST‐NAc circuit characterizes a silent period mediated by delta waves. This mechanism ensures that specific cortical computations are protected from external interference and allows for cross‐regional information transfer in a clean electrophysiological environment.

The spike‐based SNR defined in this study focuses on measuring signal gain and identifiability, which directly determines the physical transmission efficiency in the BNST‐NAc circuit. According to Shannon's information theory, channel capacity is determined by both bandwidth and SNR. From an information‐theoretic perspective, an increase in SNR is consistent with an enhanced capacity for information transmission, suggesting that IPIS may optimize the efficiency of inter‐regional communication. In the BNST‐NAc circuit, firing from pair‐recorded neurons carrying emotional regulation information constitutes the core signal, while random background firing or pathological hyperactivity is considered noise. Silent windows created by delta wave troughs significantly reduce the baseline entropy of the system by selectively inhibiting nonspecific firing. This mechanism physically enhances the detection sensitivity of downstream NAc neurons acting as receivers, which allows each BNST pulse to transmit information with higher certainty. Analysis from the perspective of signal detection theory explains that hyperactivity of the BNST in a depressive state leads to communication failure. Consequently, SNR is a core biological indicator describing the high‐fidelity information exchange capability of the BNST‐NAc circuit.

The application of the FOOOF algorithm to decouple aperiodic and periodic components of the power spectrum is of central value in analyzing DBS modulation mechanisms [66]. Traditional frequency band power analysis is often limited by the superposition effect of signals. This limitation prevents researchers from distinguishing whether power changes originate from a shift in the overall E/I balance or the restoration of specific circuit synchrony [67]. By separating the aperiodic background, this study found that the steepening of the PSD slope induced by DBS is a universal response among subjects [68]. However, the specific oscillation recovery unique to responders only became apparent after the interference of the aperiodic slope was eliminated. This analysis strategy effectively avoids assessment bias caused by changes in the background slope. It is essential to define the direct electrophysiological effects and the indirect circuit repair roles of DBS.

In the LFP‐based cross‐species machine learning algorithm, we primarily used the LFP PSD slope, sample entropy, and delta activity to determine excitatory and inhibitory periods. Theoretical models and experimental evidence show that the slope is flatter when background neuronal firing noise increases and steeper when it decreases [33, 69]. This is accompanied by an increase in complexity resulting from irregular firing [70, 71, 72]. Concurrently, the neuronal DOWN‐state is considered to occur in synchrony with delta oscillations [73, 74]. Therefore, the algorithm, designed based on foundational neuroscience principles, effectively distinguished between excitatory and inhibitory cycles. By combining the periodic components of the LFP, namely neural oscillations, with its aperiodic components, namely features extracted from the LFP, the algorithm collectively reflects the state of the underlying inhibitory network. It may become a more robust and parsimonious biomarker of therapeutic state than any specific oscillation band.

Applying findings from basic neuroscience to clinical psychiatric disorders is a significant challenge. This study leverages the high degree of conservation in the electrophysiological mechanisms and synaptic structures of mammalian neural circuits to translate mechanistic discoveries from mouse models to human TRD patients. We found that in patients for whom DBS treatment was effective, the LFP in their BNST and NAc exhibited characteristic changes corresponding to functional recovery in the animal model, such as a steeper PSD slope and enhanced theta power in the BNST, and enhanced gamma oscillation in the NAc. More importantly, the information flow from the BNST to the NAc was significantly enhanced in the treatment‐effective group. This indicates that the recovery of IPIS‐mediated communication we observed in the animal model has corresponding and measurable homologous biomarkers at the human LFP level. To more directly verify the restoration of excitatory‐inhibitory (E/I) cycles in the human brain, we developed a cross‐species machine learning model. The model successfully inferred functional inhibitory and excitatory periods from macroscopic human LFP signals using patterns learned from mouse single‐unit firing data. Applying this model, we confirmed that the proportion of inhibitory periods was significantly higher in the treatment‐effective group, and the functional connectivity between the BNST and NAc was also stronger during these periods. This provides direct quantitative evidence for the theory that DBS therapy restores the E/I balance in the human brain. Finally, through a clinical follow‐up of up to 2 years and a double‐blind, crossover randomized controlled trial (RCT), we validated the clinical utility and robustness of these novel biomarkers.

Ablation studies of the deep learning model provide supporting evidence for the core conclusion that DBS exerts antidepressant effects by restoring signal transmission mediated by the inhibitory phase [75]. Feature importance analysis shows that relying solely on traditional LFP spectral features cannot effectively distinguish between remission and depressive states in an independent RCT validation set. However, when E/I cycle features and related circuit connectivity are integrated into the model, classification accuracy improves significantly and allows for the precise identification of pathological states. Key features in the classification process are likely central to the therapeutic mechanism. This improvement in classification performance indicates a strong statistical dependency between clinical symptom improvement and the restoration of specific communication patterns. These findings demonstrate that the antidepressant mechanism of DBS is not simple neural inhibition. Instead, DBS restores the dynamic balance of the entire circuit by precisely reconstructing the network inhibitory phase and the high‐fidelity signal transmission pathways it mediates.

Distinguishing between physical target deviation and systemic mechanistic differences is essential when investigating the causes of non‐responsiveness [76]. Spatial reconstruction confirmed the accuracy of electrode implantation [77]. FOOOF analysis provided supporting evidence. During DBS activation, the aperiodic PSD slope in nonresponders showed a significant steepening trend comparable to that in responders, which reflects the effective induction of local inhibitory tone. This indicates that the BNST in nonresponders was successfully inhibited by electrical stimulation. The lack of clinical efficacy did not arise from a failure to target the nucleus but from functional rigidity within the neural circuit. Specifically, the circuit could not generate the compensatory neural activity required for functional repair. Furthermore, this study found that HAMD scores did not immediately return to baseline during the washout period after DBS was deactivated. This persistent effect contrasts with the rapid symptom recurrence observed in Parkinson's disease after deactivation and is more consistent with the delayed and lasting biological effects of antidepressant drugs [78]. These results suggest that the antidepressant mechanism of DBS involves both real‐time electrical modulation and the induction of deep neuroplastic changes, which potentially involve synaptic remodeling triggered by chronic stimulation [79]. Therefore, limited efficacy in nonresponders may be related to intrinsic plasticity deficits that prevent the reconstruction of a healthy neural network homeostasis following physical inhibition.

This not only provides the highest level of clinical evidence for the efficacy of DBS in treating TRD but also marks a complete loop from mechanistic understanding to clinical application, paving the way for the precision DBS therapy based on objective biomarkers envisioned in the introduction.

## 4 Limitations

Despite the findings of this research, several limitations deserve consideration. This study utilizes the SNR of partner neuron firing in the BNST‐NAc circuit to infer its influence on broader dialogues. At a neuron level, the pattern in which IPIS facilitates high‐fidelity communication by maximizing SNR is considered a conserved feature. Regarding connectivity, BNST partner neurons exhibit a specific temporal relationship with NAc activity. Consequently, it is inferred that the SNR of BNST partner spikes serves as an indicator of signal integration within the emotional network. However, utilizing SNR to describe the intensity of cross‐regional brain activity is supported primarily by information theory, while direct neuroscientific observational evidence remains limited. The mechanisms of this phenomenon in broader contexts still require precise recording and clinical validation.

This research faces significant cross‐scale translational challenges when moving from microscopic spike analysis in animal models to macroscopic human LFP analysis. In the mouse model, single‐unit recordings allow for the resolution of precise neural spike patterns at the millisecond level. Conversely, human clinical data are constrained by ethical and recording technicalities, relying primarily on macroscopic LFP signals. To address this scale discrepancy, the study utilizes cross‐species algorithms to infer E/I periods from features such as the PSD slope and sample entropy of human LFP based on functional neurophysiological homology. NAc gamma activity is utilized as a shared circuit functional indicator across species. This approach manifests as IPIS analysis in animals and as neural oscillation analysis in humans. This logic is built on the hypothesis that mammalian neural circuit structures are highly conserved. While supported by clinical cohort data, this remains an indirect inference based on machine learning rather than direct observation of human single‐unit firing. Future research must integrate higher‐resolution human recording technologies to mitigate scale limitations and validate circuit communication mechanisms.

Furthermore, the results of the deep learning ablation study demonstrate the critical role of E/I‐related features in distinguishing pathological states. However, the generalization performance of these results remains uncertain when applied to larger, multi‐center, or more diverse populations. Therefore, it should be considered a robust proof‐of‐concept, and its clinical generalizability requires further investigation.

Finally, although the human LFP cohort size is substantial for a DBS study, the discovered biomarkers and validated models must be verified in broader populations. This is necessary to ensure their robustness and universality.

## Author Contributions

B.S. and S.Z. conceived, designed, and supervised the study. X.L. performed most of the experiments and data collection, with assistance from Y.W., Y.W., Y.Z., and K.Y. in behavioral testing and electrophysiological recordings, and from Y.C., Q.S., L.B., and H.P. in animal model preparation and histological analysis. X.L. and Y.W. performed the statistical analyses, which were overseen and verified by S.Z. At least two authors, S.Z. and B.S., had unrestricted access to all data. X.L. prepared the first draft of the manuscript. S.Z., B.S., and V.V. reviewed and edited the manuscript, with all authors contributing to the review process. All authors agreed to submit the manuscript, read and approved the final draft, and take full responsibility for its content, including the accuracy of the data and the integrity of the work.

## Conflicts of Interest

The authors declare no conflicts of interest.

## Supporting information




**Supporting File 1**: advs74542‐sup‐0001‐SuppMat.pdf.

**Supporting File 2**: advs74542‐sup‐0002‐SuppMat.docx.

## Data Availability

The data that support the findings of this study are available on request from the corresponding author. The data are not publicly available due to privacy or ethical restrictions.
