# Supplementary material for: Deep Brain Stimulation Induces Antidepressant Effects by Restoring High‐Fidelity Communication in the BNST‐NAc Circuit
Source: Adv Sci (Weinh). 2026 Mar 9;13(25):e21943. doi: 10.1002/advs.202521943 (PMC13137839; doi:10.1002/advs.202521943)
Supplement: Supplementary file 1 — Supporting File 1: advs74542‐sup‐0001‐SuppMat.pdf. [file ADVS-13-e21943-s002.pdf]

Supplementary Figure 1

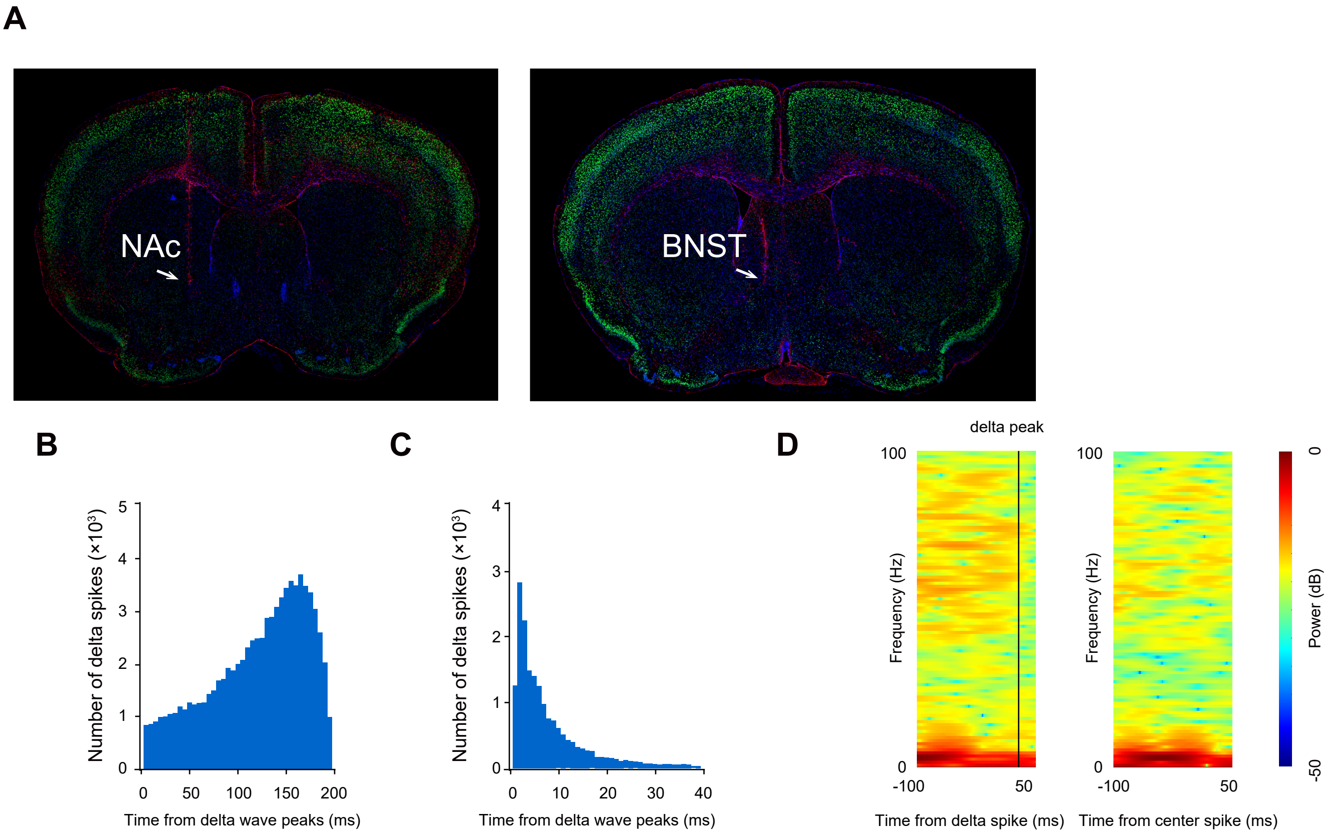

Supplementary Figure 1. Characteristics of inhibitory period isolated spikes from partner cells in the BNST-NAc circuit. (A) Electrode Tip Localization Diagram.Multi-immunofluorescence staining illustrating electrode implantation sites in the BNST (right) and NAc (left). The electrode tract appears as a void in DAPI (blue) signal and a strong positive band of GFAP (red) signal. (B) Time distribution of spikes discharged by BNST delta-silent units. (C) Time distribution of spikes discharged by BNST non-delta-silent units. (D) Spectrogram schematics illustrate NAc power during spikes around the peri-delta period (left) versus center spikes during the peri-excitatory period (right) within the BNST-NAc circuit.

Supplementary Figure 2

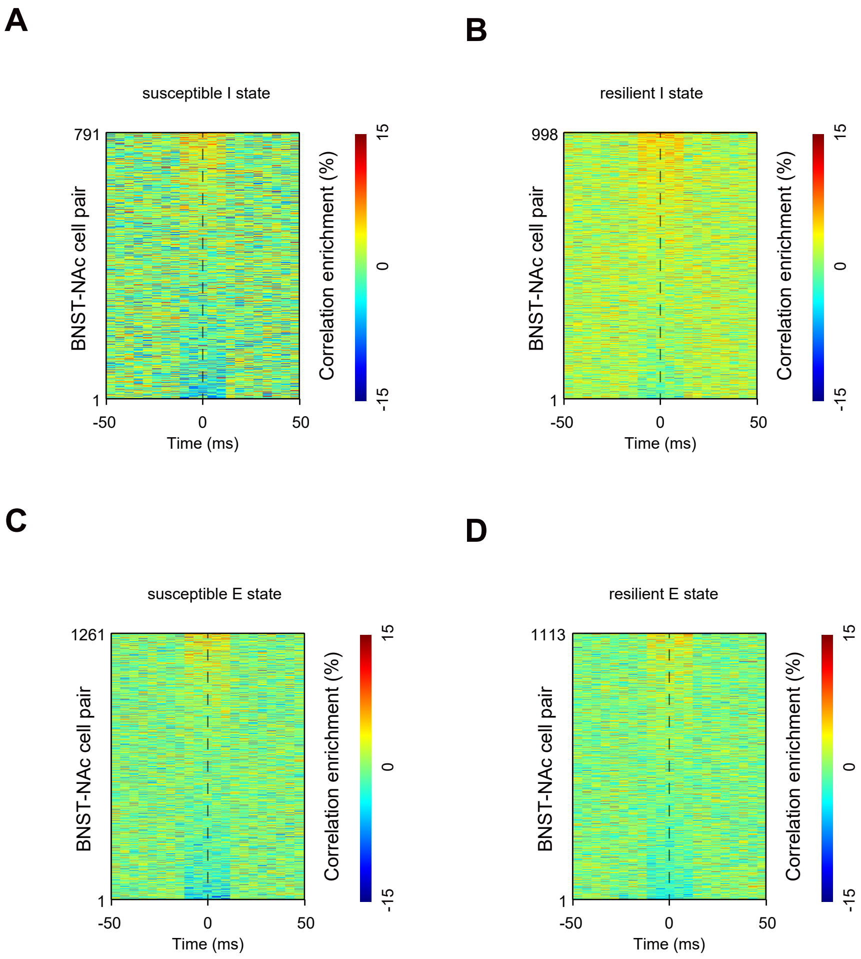

Supplementary Figure 2. Changes in BNST-NAc circuit neuronal correlation after learned helplessness modeling. (A) Enrichment in positive correlations for the BNST-NAc spikes shown in Figure 2N. (B) Enrichment in positive correlations for the BNST-NAc spikes shown in Figure 2O. (C) Enrichment in positive correlations for the BNST-NAc spikes shown in Figure 2P. (D) Enrichment in positive correlations for the BNST-NAc spikes shown in Figure 2Q.

Supplementary Figure 3

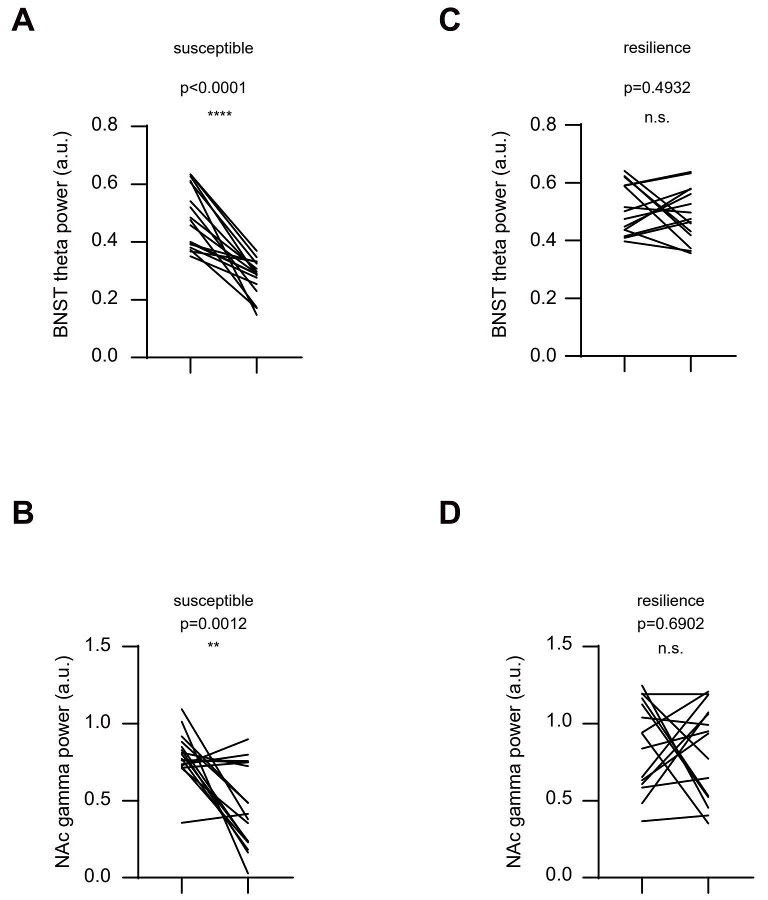

Supplementary Figure 3. LFP connectivity-related features in the BNST-NAc circuit after learned helplessness modeling. (A) Changes in wPLI in the stress-susceptible group (pre- vs. post-modeling, paired t-test, n=17, p=0.0039). (B) Changes in BNST LFP PSD slope in the stress-susceptible group (pre- vs. post-modeling, paired t-test, n=17, p<0.0001). (C) Changes in BNST LFP entropy in the stress-susceptible group (pre- vs. post-modeling, paired t-test, n=17, p=0.2945). (D) Changes in wPLI in the stress-resilient group (pre- vs. post-modeling, paired t-test, n=15, p<0.0001). (E) Changes in BNST

Supplementary Figure 4

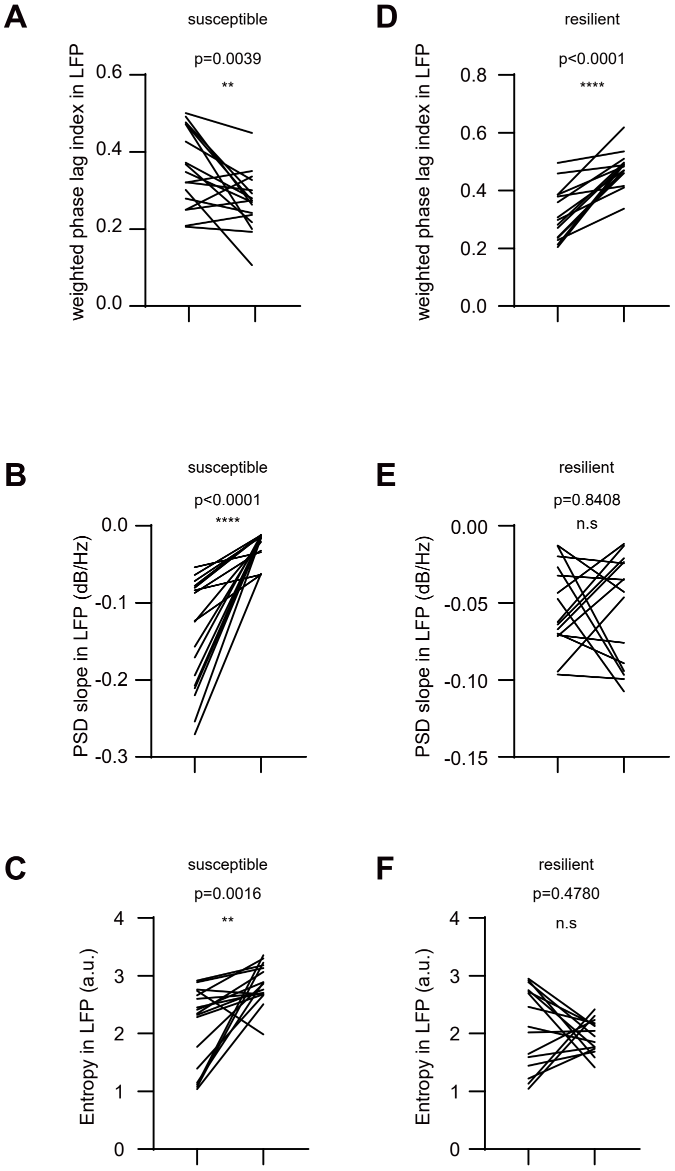

Supplementary Figure 4. LFP spectral features in the BNST-NAc circuit after learned helplessness modeling. (A) Changes in BNST theta power in the stress-susceptible group (pre- vs. post-modeling, paired t-test, n=17, p<0.0001). (B) Changes in NAc gamma power in the stress-susceptible group (pre- vs. post-modeling, paired t-test, n=17, p=0.0012). (C) Changes in BNST theta power in the stress-resilient group (pre- vs. post-modeling, paired t-test, n=17, p=0.4932). (D) Changes in NAc gamma power in the stress-resilient group (pre- vs. post-modeling, paired t-test, n=17, p=0.6902).

Supplementary Figure 5

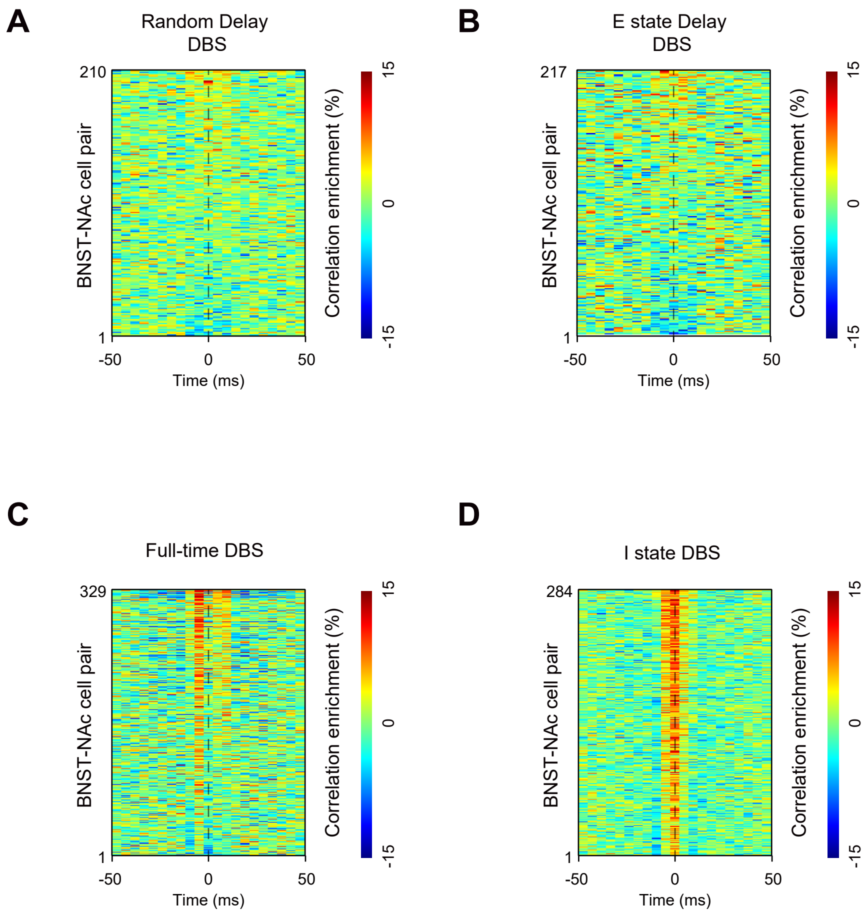

Supplementary Figure 5. Effect of different DBS paradigms on BNST-NAc neuronal firing cross-correlation enrichment. (A) Enrichment in positive correlations for the BNST-NAc spikes shown in Figure 3R. (B) Enrichment in positive correlations for the BNST-NAc spikes shown in Figure 3S. (C) Enrichment in positive correlations for the BNST-NAc spikes shown in Figure 3T. (D) Enrichment in positive correlations for the BNST-NAc spikes shown in Figure 3U.

Supplementary Figure 6

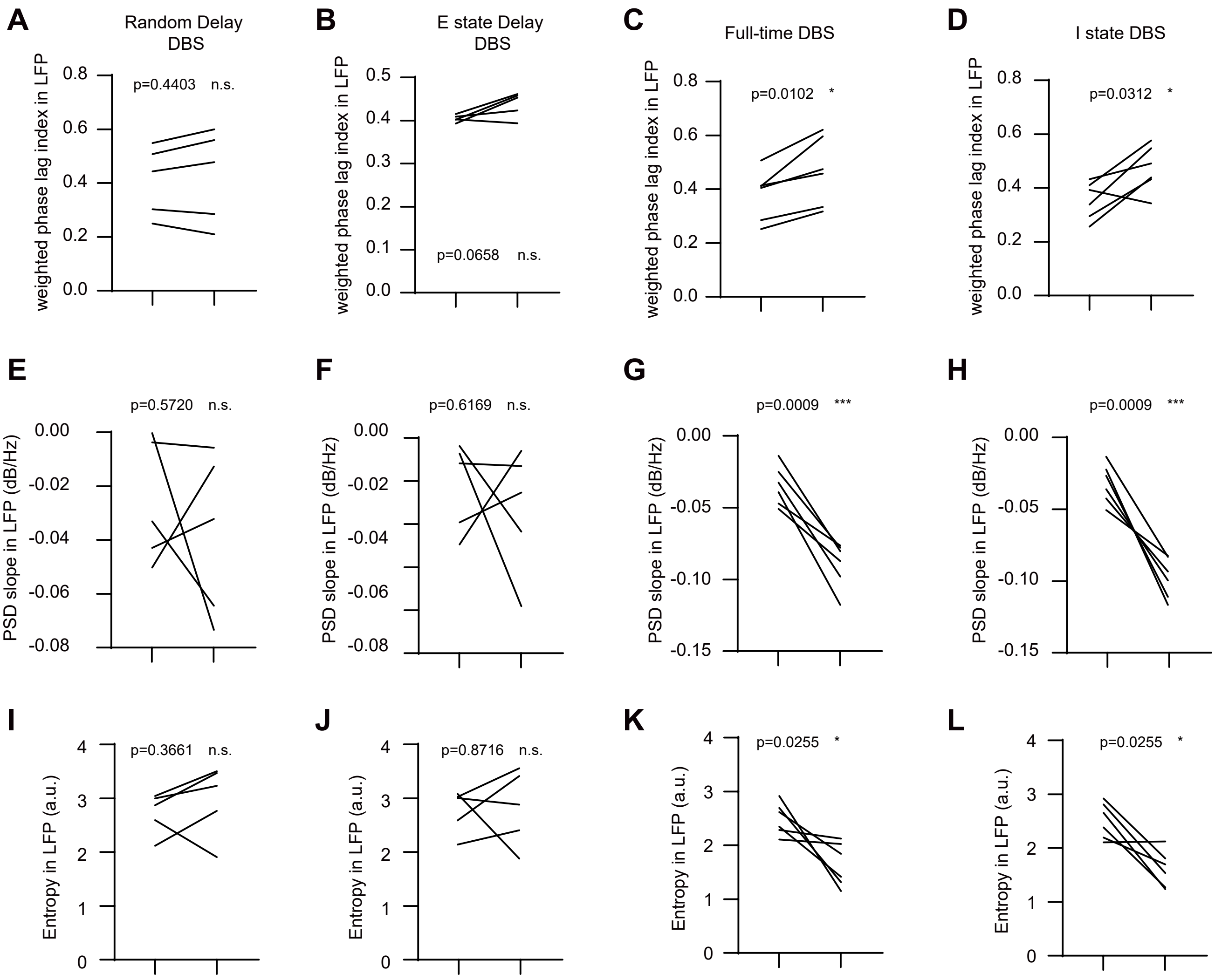

Supplementary Figure 6. Effect of different DBS paradigms on BNST-NAc circuit LFP connectivity-related features. (A) Changes in wPLI with Random delay DBS (pre- vs. post-DBS, paired t-test, n=5, p=0.4403). (B) Changes in wPLI with E state DBS (pre- vs. post-DBS, paired t-test, n=5, p=0.0658). (C) Changes in wPLI with Full time DBS (pre- vs. post-DBS, paired t-test, n=6, p=0.0102). (D) Changes in wPLI with I state DBS (pre- vs. post-DBS, paired t-test, n=6, p=0.0312). (E) Changes in BNST PSD slope with Random delay DBS (pre- vs. post-DBS, paired t-test, n=5, p=0.5720). (F) Changes in BNST PSD slope with E state DBS (pre- vs. post-DBS, paired t-test, n=5, p=0.6169). (G) Changes in BNST PSD slope with Full time DBS (pre- vs. post-DBS, paired t-test, n=6, p=0.0009). (H) Changes in BNST PSD slope with I state DBS (pre- vs. post-DBS, paired t-test, n=6, p=0.0009).LFP PSD slope in the stress-resilient group (pre- vs. post-modeling, paired t-test, n=15, p=0.8408). (F) Changes in BNST LFP entropy in the stress-resilient group (pre- vs. post-modeling, paired t-test, n=15, p=0.2945).

Supplementary Figure 7

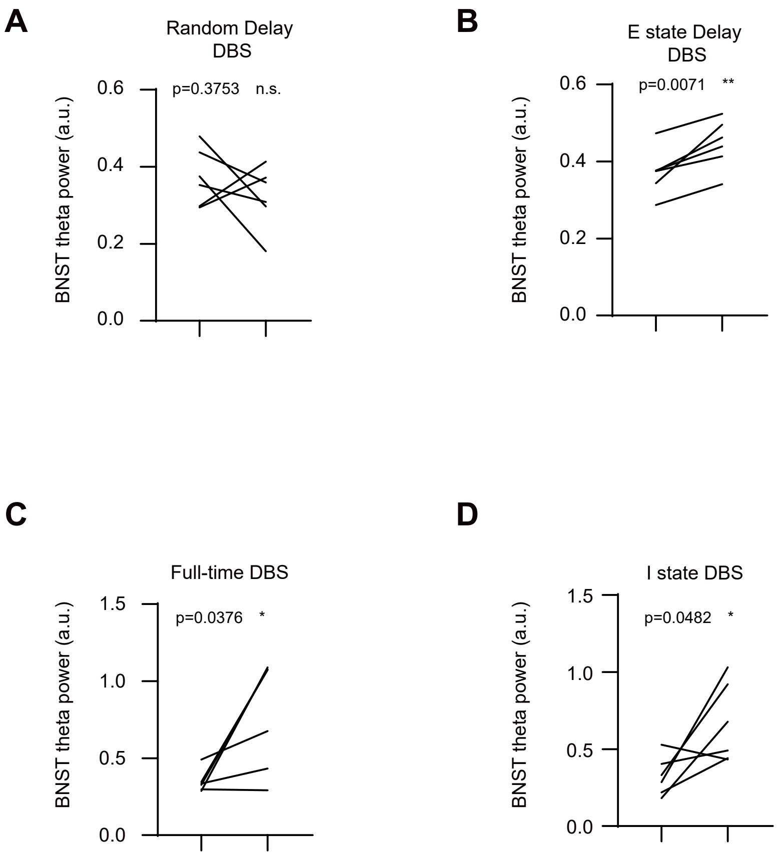

Supplementary Figure 7. Effect of different DBS paradigms on BNST LFP spectral features. (A) Changes in BNST theta power with Random delay DBS (pre- vs. post-DBS, paired t-test, n=5, p=0.3753). (B) Changes in BNST theta power with E state DBS (pre- vs. post-DBS, paired t-test, n=5, p=0.0071). (C) Changes in BNST theta power with Full time DBS (pre- vs. post-DBS, paired t-test, n=6, p=0.0376). (D) Changes in BNST theta power with I state DBS (pre- vs. post-DBS, paired t-test, n=6, p=0.0482).

Supplementary Figure 8

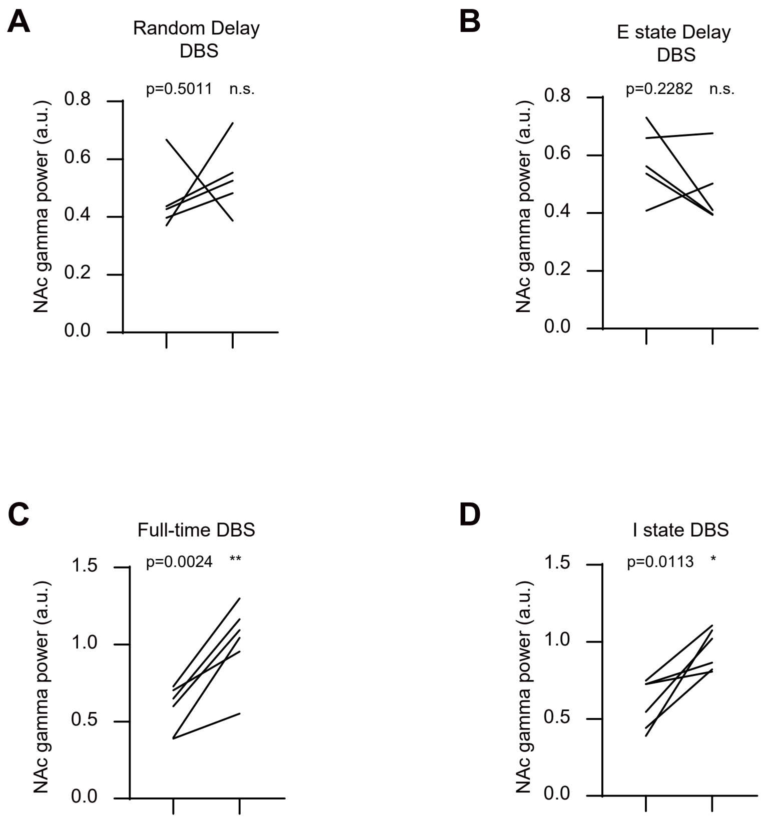

Supplementary Figure 8. Effect of different DBS paradigms on NAc LFP spectral features. (A) Changes in NAc gamma power with Random delay DBS (pre- vs. post-DBS, paired t-test, n=5, p=). (B) Changes in NAc gamma power with E state DBS (pre- vs. post-DBS, paired t-test, n=5, p=). (C) Changes in NAc gamma power with Full time DBS (pre- vs. post-DBS, paired t-test, n=6, p=). (D) Changes in NAc gamma power with I state DBS (pre- vs. post-DBS, paired t-test, n=6, p=).

Supplementary Figure 9

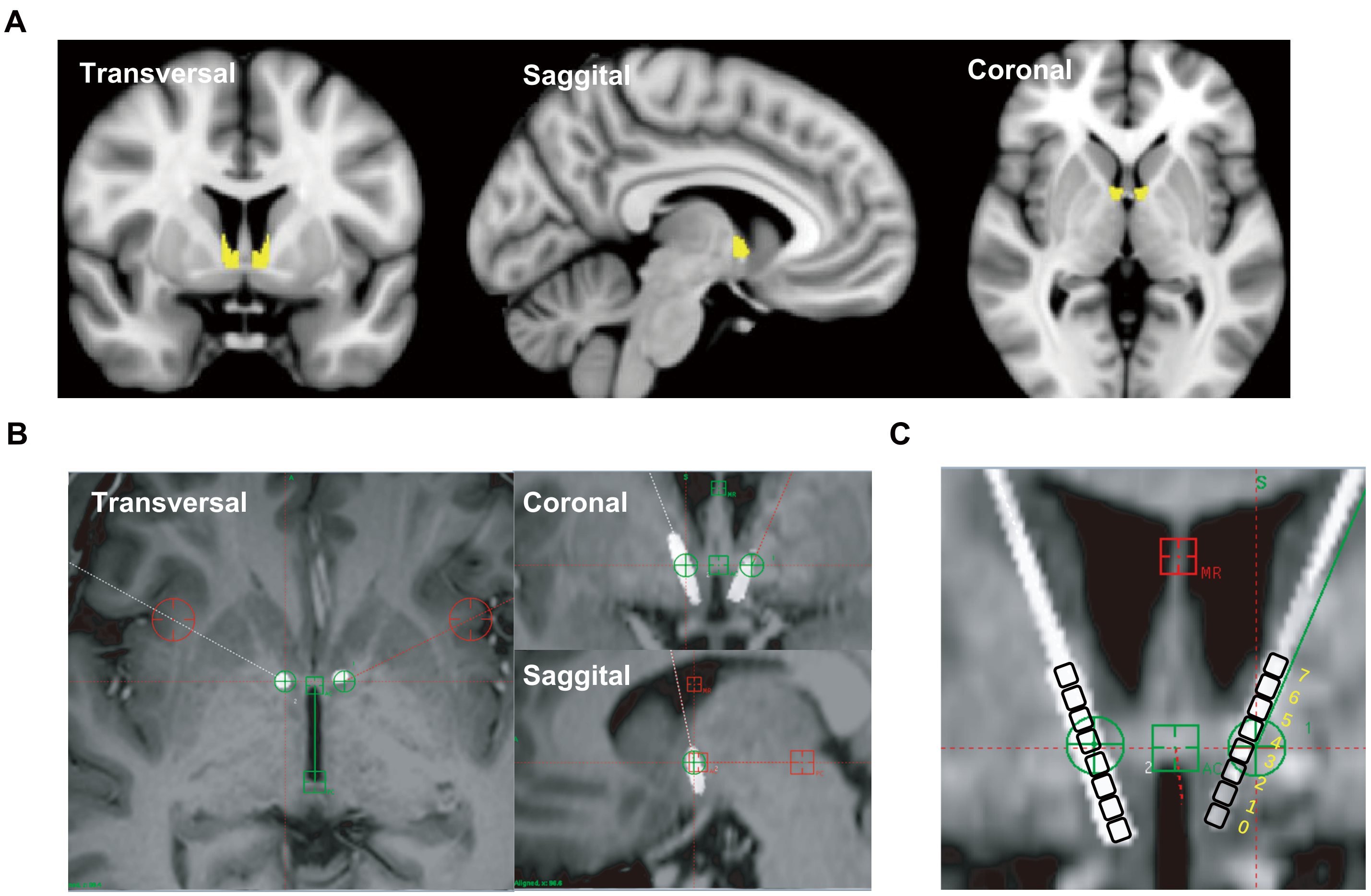

Supplementary Figure 9. Imaging localization of surgically implanted DBS electrodes. (A) Schematic of structural MRI scan of the human BNST. The anatomical location of the human BNST (T2W MRI scan). (B) Preoperative MRI and postoperative CT fusion images showing electrode placement (highlighted by white area). (C) Enlarged image from coronal plane CT for the microstructure of bilaterally implanted electrodes. Contacts were highlighted by black rectangles.

Supplementary Figure 10

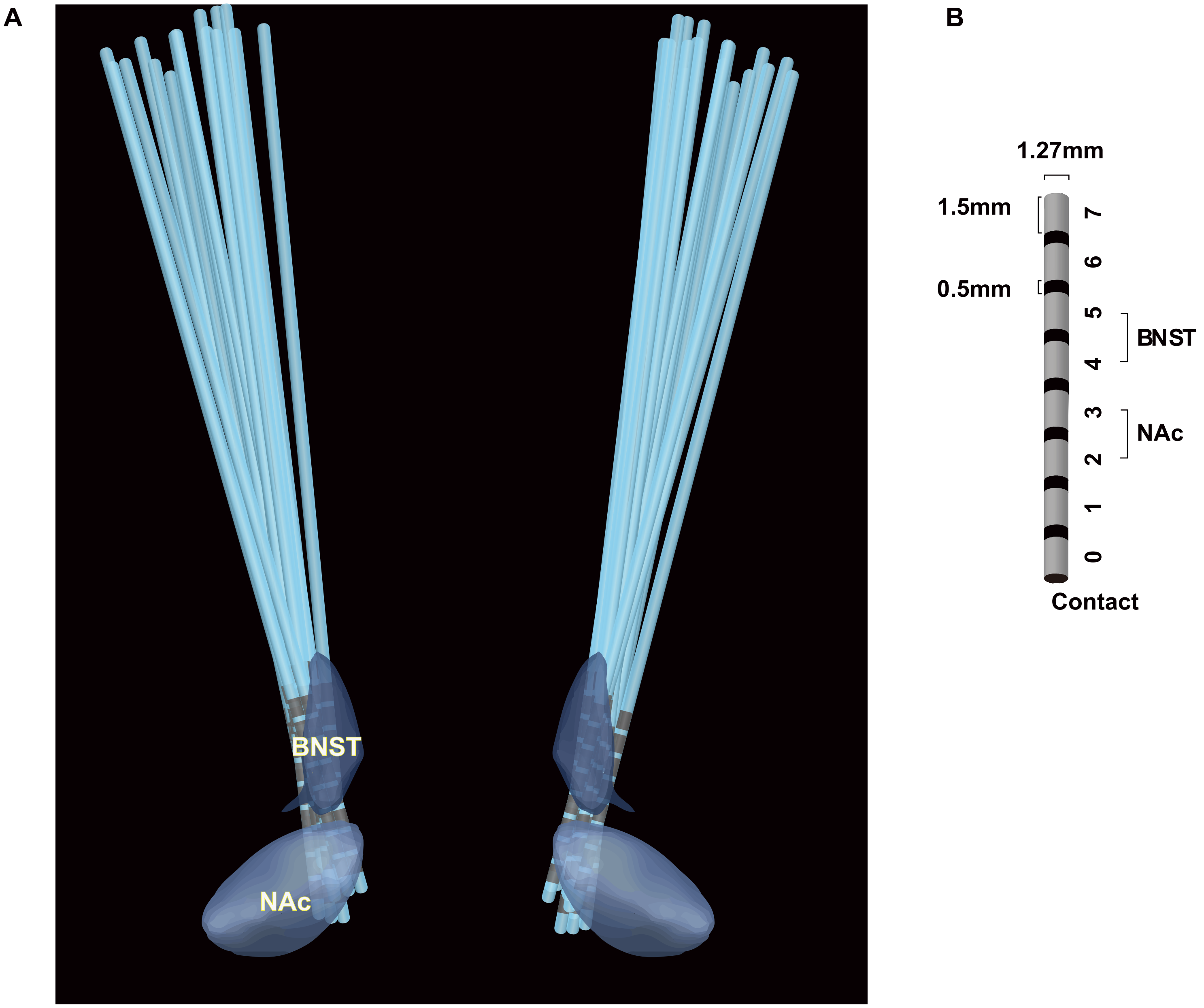

Supplementary Figure 10. Lead-DBS localization of surgically implanted DBS electrodes. (A) Lead-DBS electrode reconstruction on both sides of the cerebral hemispheres of all recruited patients. Subcortical structures are based on a CIT168 Reinf Learn atlas [dark blue: bed nucleus of the stria terminalis (BNST), light blue: nucleus accumbens (NAc)] laid over a 7T MRI ex vivo 100-μm thick human brain background template. Arrows show electrode contacts used for stimulation. Although electrodes are implanted bilaterally, only one side was reconstructed for stimulation and recording purposes here. (B) Schematic representation of electrode contacts. The specific contacts included in the analysis were determined by individualized lead-DBS reconstruction.

Supplementary Figure 11

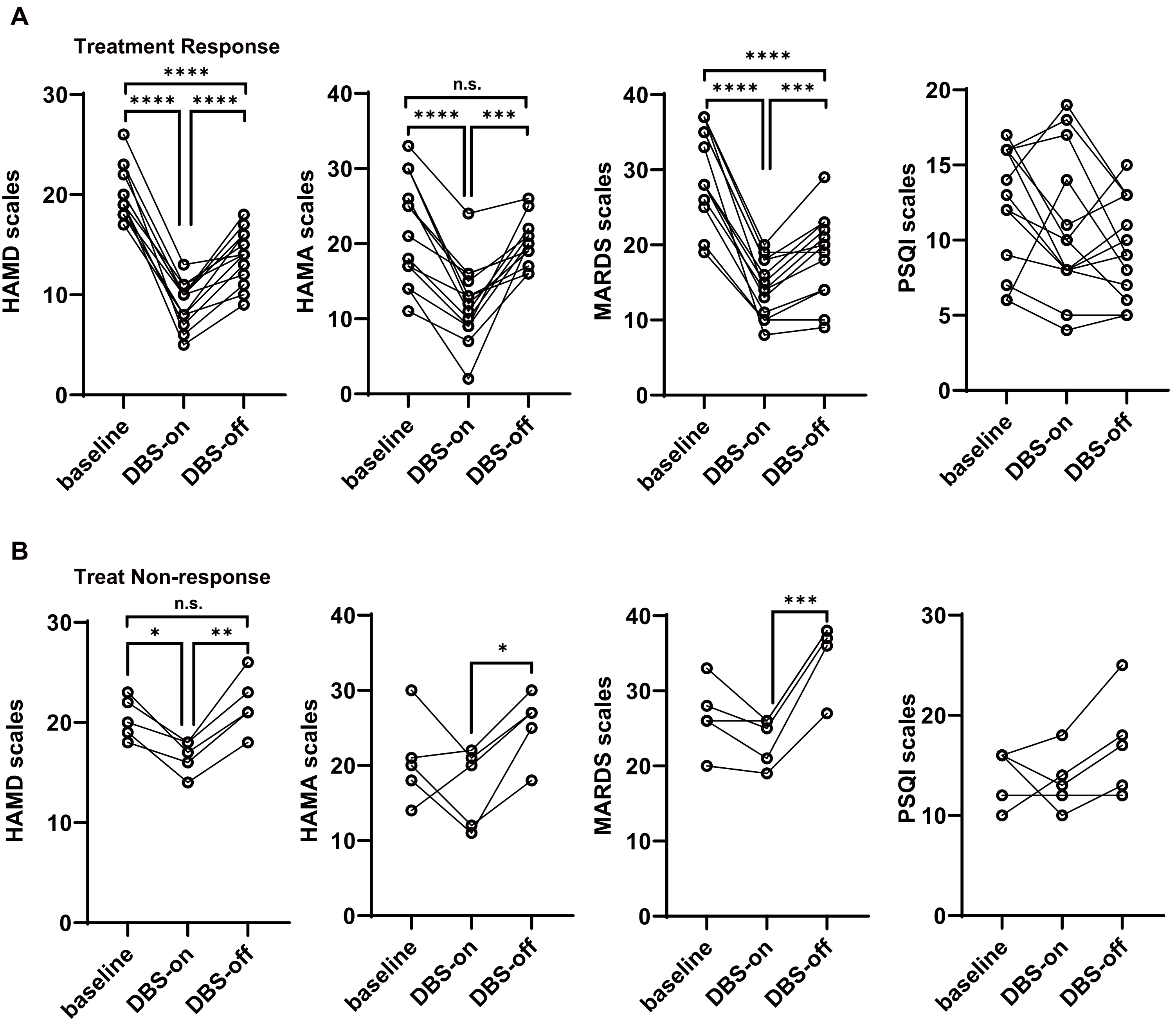

Supplementary Figure 11. Therapeutic DBS significantly improved clinical scales of the majority of TRD patients. (A) Clinical scales at baseline and after the 6-month treatment of DBS in the treatment response (TR) group. For the HAMD-17 scale, the average improvement ratio was 56.80% (repeated measures ANOVA with Bonferroni's multiple comparisons correction: adjusted $p < 0.0001$). For the HAMA-14, the average improvement ratio was 44.17% ($p < 0.0001$). For the MADRS, the average improvement ratio was 51.40% ($p < 0.0001$). For the PSQI, no significant improvement was observed ($p = 0.4739$). (B) Clinical scales at baseline and after the 6-month treatment of DBS in the treatment non-response (TN) group. For the HAMD-17, the average improvement ratio was 18.34% ($p = 0.0195$). For the HAMA-14, no significant improvement was observed ($p = 0.5352$). For the MADRS, no significant improvement was observed ($p = 0.1362$). For the PSQI, no significant improvement was observed ($p = 0.9221$).

Supplementary Figure 12

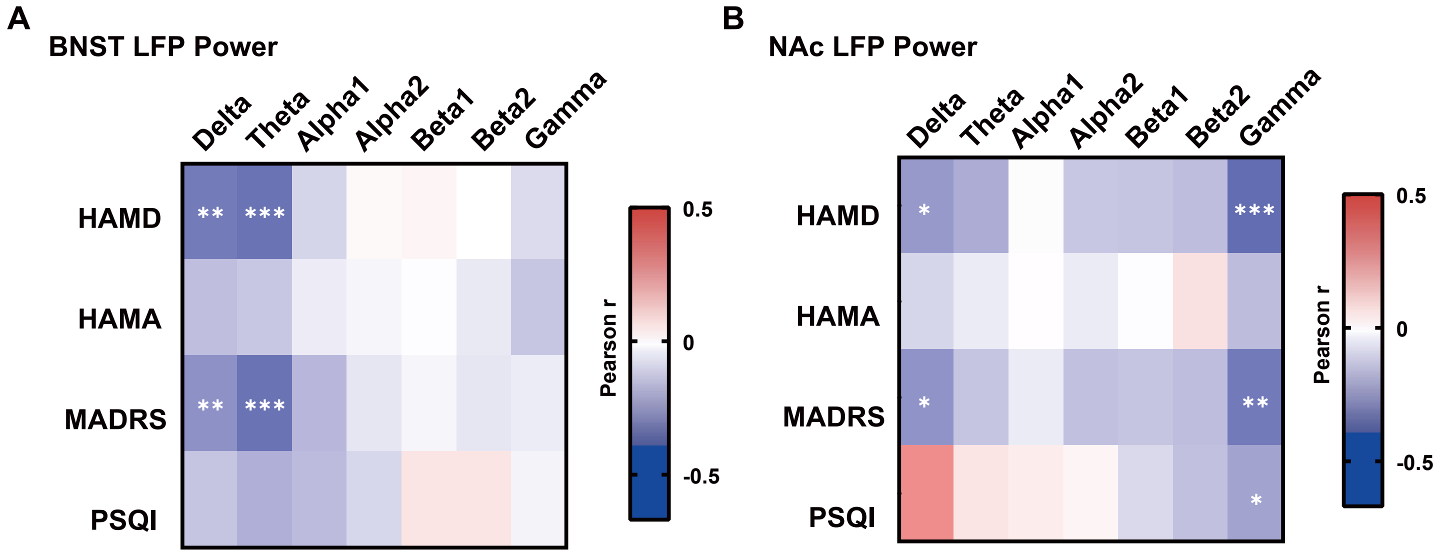

Supplementary Figure 12. Most activated rhythms in the BNST-NAc circuit correlated with clinical symptoms of TRD patients. (A) Heat map of a matrix of Pearson's correlation coefficients between clinical scales (HAM-D, HAM-A, MADRS, and PSQI) and six normalized power bands (delta, theta, alpha1, alpha2, beta1, beta2) recorded from the BNST in the TR group. (B) Heat map of a matrix of Pearson's correlation coefficients between clinical scales and six normalized power bands recorded from the NAc in the TN group.

Supplementary Figure 13

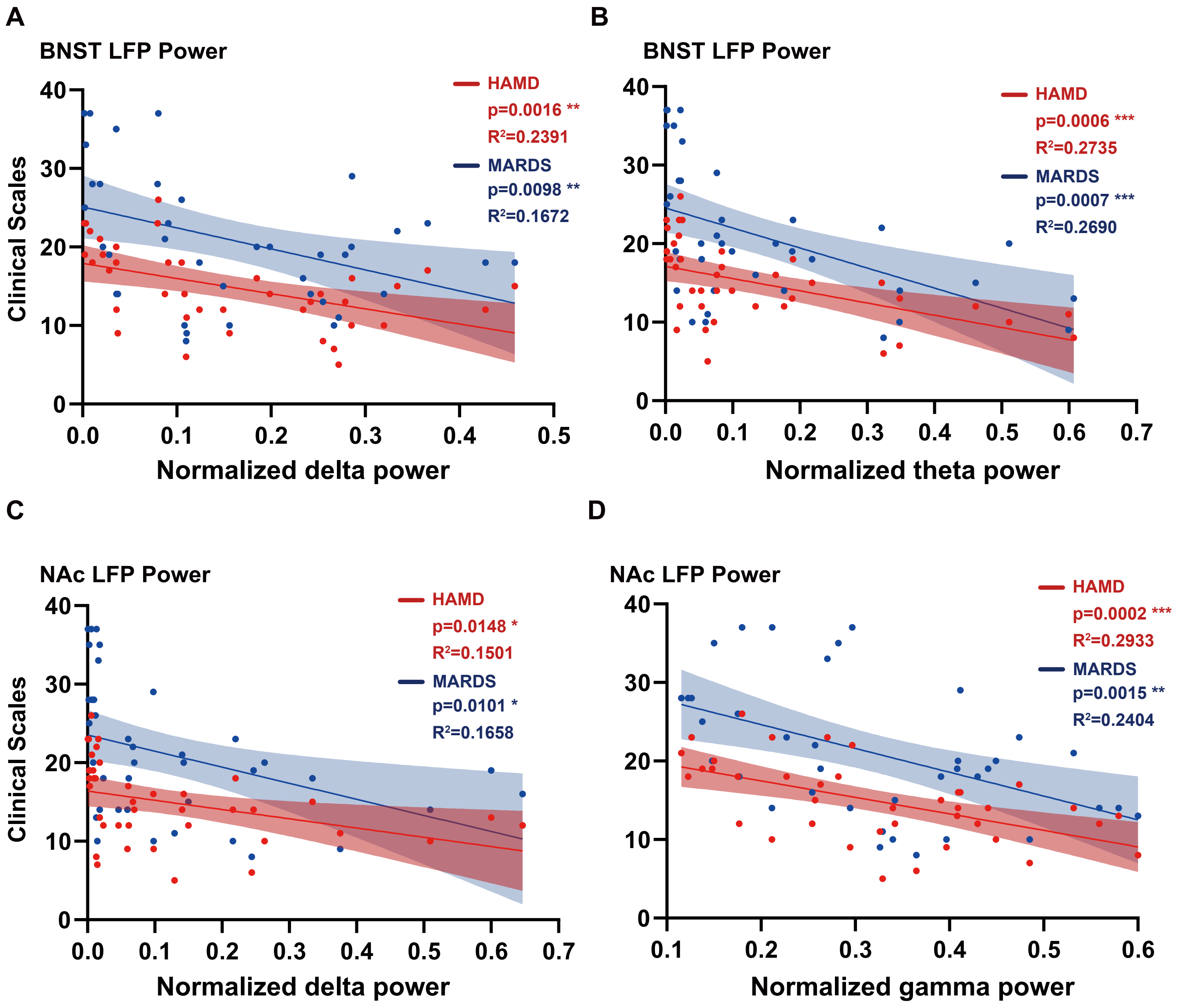

Supplementary Figure 13. Correlation between LFP power and clinical symptoms. (A) Regression analysis showing the significant relationship between BNST-delta power and depressive symptoms. The line indicates the linear regression equation, and the shadowed area indicates a 95% confidence interval. (B) Regression analysis showing the significant relationship between BNST-gamma power and depressive symptoms. (C) Regression analysis showing the significant relationship between NAc-delta power and depressive symptoms. (D) Regression analysis showing the significant relationship between BNST-theta power and depressive symptoms.

Supplementary Figure 14

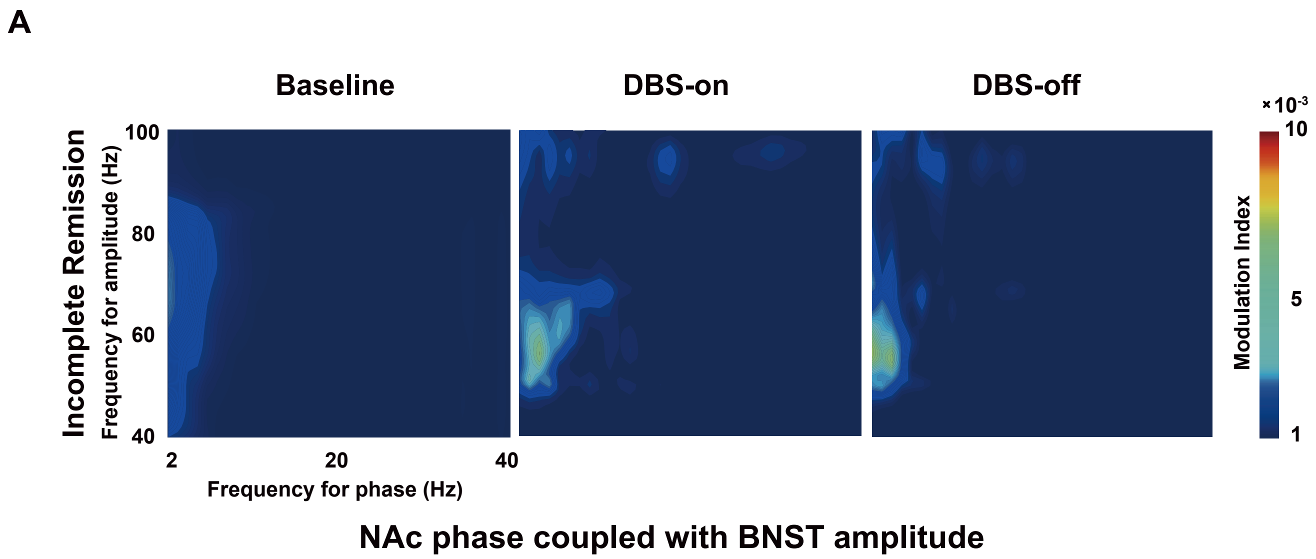

Supplementary Figure 14. Phase-amplitude coupling in non-responders. (A) Representative PAC heatmap observed at three states (left: baseline, middle: DBS-on, right: DBS-off) in the TN group.

Supplementary Figure 15

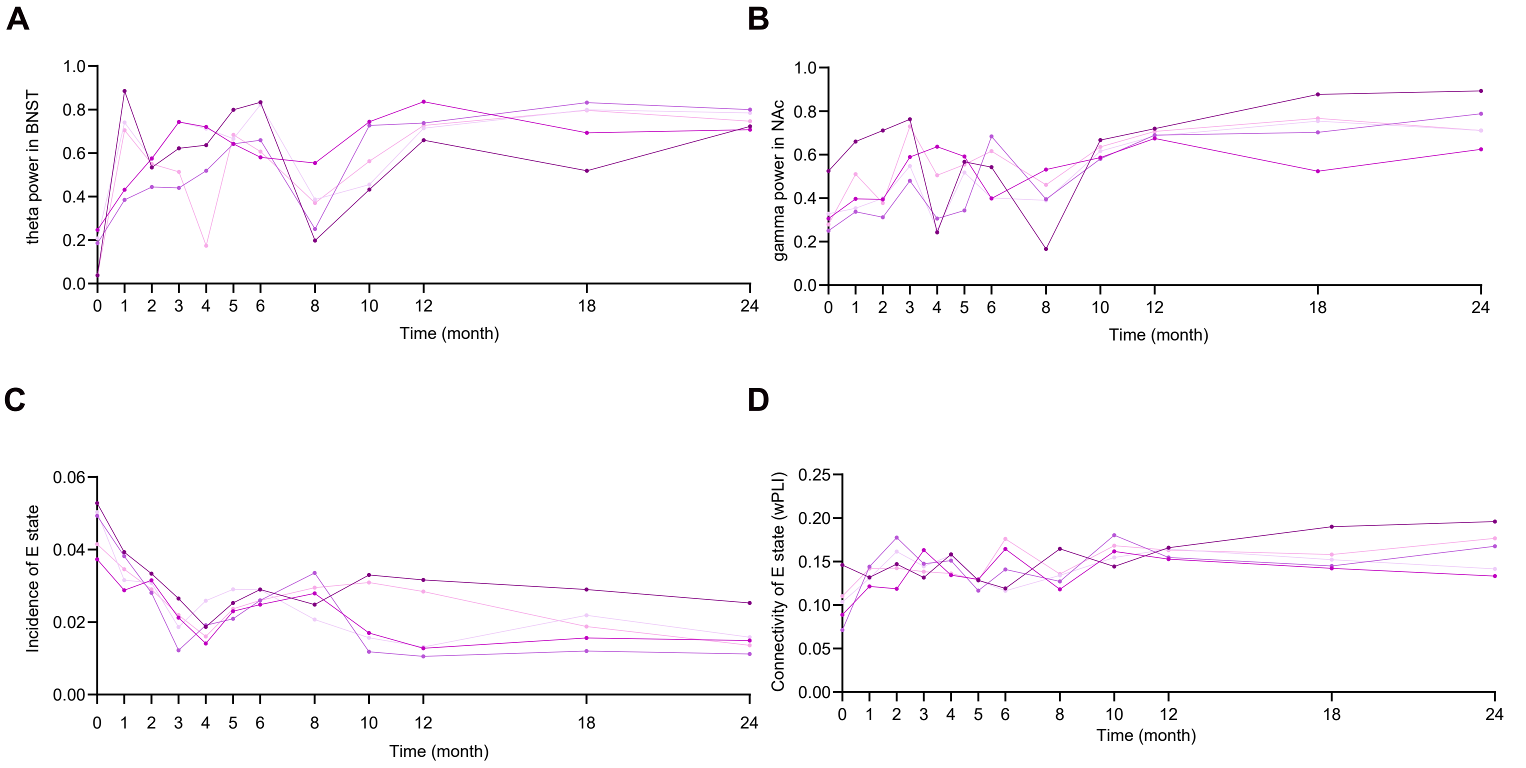

Supplementary Figure 15. Longitudinal follow-up. (A) Changes in a specific power feature (BNST theta) from patients' LFP over the 24-month follow-up. (B) Changes in a specific power feature (NAc gamma) from patients' LFP over the 24-month follow-up. (C) Changes in the incidence of intra-circuit excitatory states (E states) from patients' LFP over the 24-month follow-up. (D) Changes in cross-regional signal transmission strength within E states over the 24-month follow-up.

Supplementary Figure 16

A

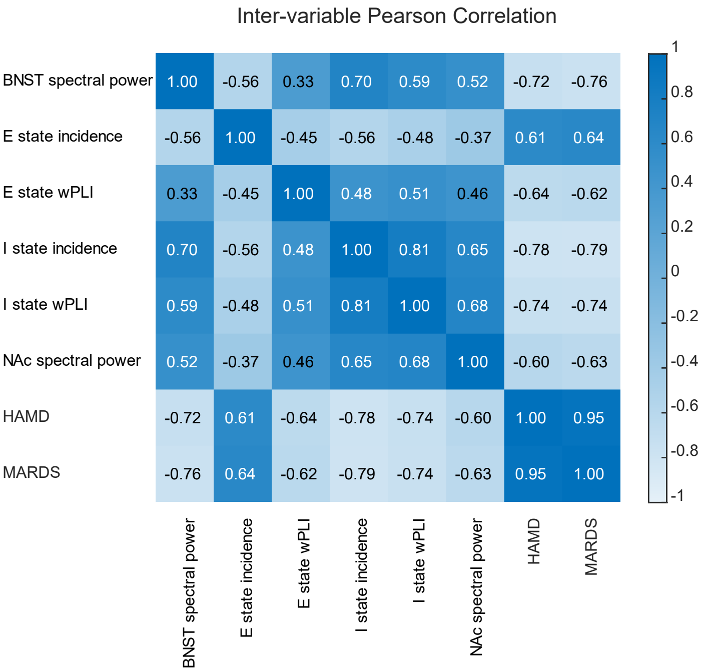

B

Predictor	HAMD (Fixed Effects)		
	Estimate	Std. Error	P-value
(Intercept)	33.887	3.332	<0.001
Time	-0.078	0.073	0.293
BNST spectral power	-7.857	2.431	0.002
E state incidence	31.122	50.235	0.538
E state wPLI	-68.333	18.557	<0.001
I state incidence	-127.92	58.786	0.034
I state wPLI	-8.571	5.401	0.119
NAc spectral power	1.893	3.266	0.565
---	---	---	---
Marginal R2 (Rm2)		0.7808	
Conditional R2 (Rc2)		0.7808	
Predictor	MADRS (Fixed Effects)		
	Estimate	Std. Error	P-value
(Intercept)	37.111	3.386	<0.001
Time	-0.07	0.076	0.363
BNST spectral power	-9.934	2.473	<0.001
E state incidence	66.496	51.02	0.198
E state wPLI	-61.872	18.779	0.002
I state incidence	-113.46	59.688	0.063
I state wPLI	-7.548	5.483	0.174
NAc spectral power	-0.731	3.335	0.827
---	---	---	---
Marginal R2 (Rm2)		0.8075	
Conditional R2 (Rc2)		0.8119	

C

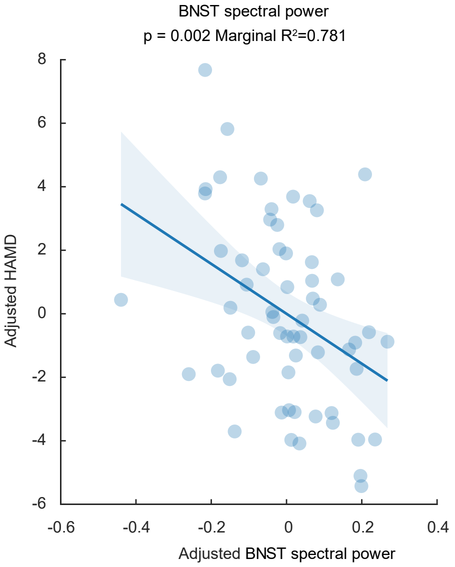

D

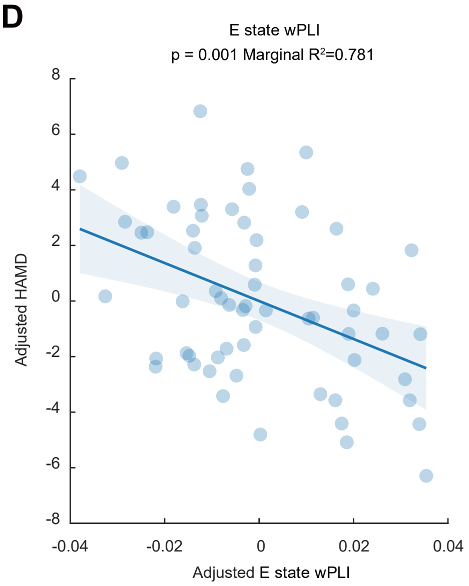

E

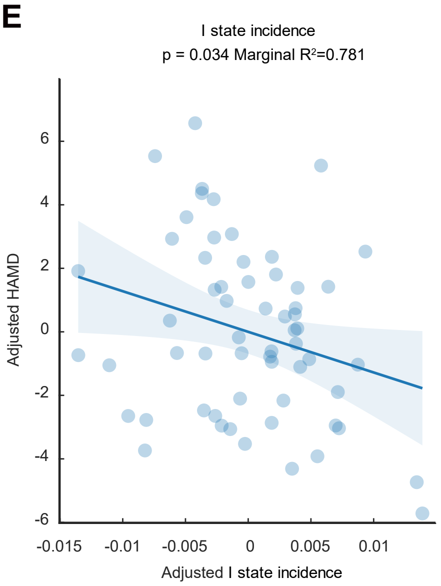

F

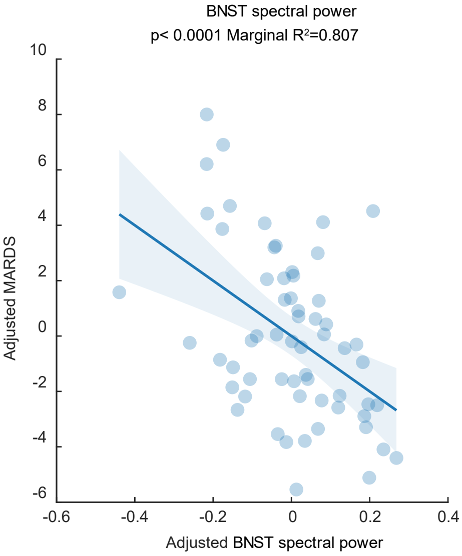

G

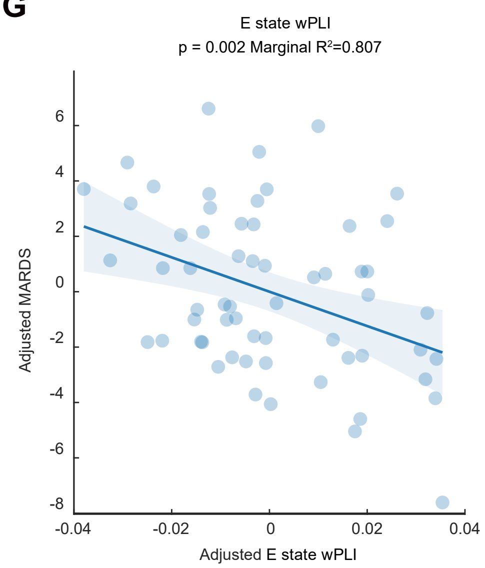

Supplementary Figure 16. Multiple Linear Regression Model Statistics

(A) Global Pearson correlation heatmap between neuro-electrophysiological features and clinical scores. Heatmap aims to assess the raw pairwise associations between neuro-feature parameters and clinical depression scales (HAMD and MADRS) across all subjects throughout the entire follow-up period (24 weeks, 60 observation points in total). Calculations are based on bivariate Pearson correlation analysis, utilized to preliminarily identify indices with potential predictive value prior to constructing complex multi-variate models. The heatmap indicates that BNST power and functional connectivity indices exhibit a clear negative correlation trend with HAMD and MADRS scores at the raw observation level.

(B) Summary of linear mixed-effects model (LME) statistical results for the contribution of neural predictors to clinical symptom improvement. Table summarizes the two core statistical models constructed to identify independent biomarkers. To correct for individual heterogeneity in longitudinal repeated measures data and interference from follow-up time, we employed linear mixed-effects models (LME), which include Fixed Effects (6 neural parameters and Time) and Random Effects (random intercepts for each subject and random slopes for Time). This design allows for the extraction of the net contribution of neural features to the clinical condition after controlling for "natural recovery over time" and "individual baseline differences."

(C) Partial regression plot of the independent predictive effect of BNST spectral power on HAMD scores

(D) Partial regression plot of the independent predictive effect of E state wPLI on HAMD scores

(E) Partial regression plot of the independent predictive effect of I state incidence on HAMD scores

(F) Partial regression plot of the independent predictive effect of BNST spectral poweron MADRS scores

(G) Partial regression plot of the independent predictive effect of E state wPLI on MADRS scores

Supplementary Figure 17

A

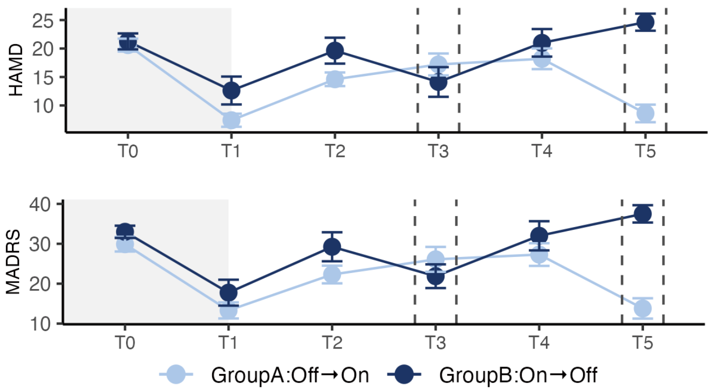

B

Mean scores during the crossover trial (n = 18)

Variable	T0	T1	T2	T3	T4	T5	Sham	Active	Changes between Sham and Active	95% CI	Treatment Effect	Crossover Effect	Period Effect
HAMD	20.9 (3.6)	9.7 (5.8)	16.8 (5.6)	15.8 (6.8)	19.4 (6.2)	15.7 (9.3)	20.5 (6.4)	11.1 (6.6)	9.4 (7.1)	5.9~13.0	32.2 (<.001)	3.9 (0.066)	3.8 (0.070)
MADRS	31.3 (5.3)	15.3 (7.8)	25.4 (9.1)	24.2 (9.2)	29.4 (9.6)	24.3 (14.0)	31.2 (10.0)	17.4 (9.0)	13.8 (8.4)	9.6~18.0	28.4 (<.001)	6.0 (0.027)	2.3 (0.146)

Data were expressed in mean (SD) for rating scales and F value (P value) for effects.

Supplementary Figure 17. Statistical Results for the RCT

(A) Primary endpoint results (HAMD, MARDS reduction) of the randomized, double-blind, crossover clinical trial.This study employed a randomized, double-blind, crossover trial design. Randomization was conducted using an envelope procedure, where an independent researcher sequentially opened sealed, opaque envelopes to determine the allocation sequence, thereby ensuring allocation concealment. Patients, the two assessing psychiatrists, and other relevant researchers remained blinded throughout the crossover phase. A total of 18 patients entered this stage, with 10 randomized to receive sham stimulation for two weeks followed by active stimulation (the sham-active group), and 8 randomized to the reverse sequence (the active-sham group). A 2-day washout period was implemented between each phase to eliminate carryover effects.Disease fluctuations were monitored at six key time points: T0 represented the baseline at study entry; T1 was the pre-randomization phase following an open-label parameter optimization period of at least six months; T2 and T4 served as assessment points for the two washout phases; and T3 and T5 were the end-point assessments following each of the two crossover intervention phases.

The primary outcome of the trial was the difference in Hamilton Depression Scale (HAMD) scores between the active and sham stimulation phases. The results demonstrated that HAMD scores were significantly lower during active DBS than during sham DBS, with a mean difference of 9.4 points. Statistical analysis yielded a p-value of < 0.001, confirming the significance of the therapeutic effect. Furthermore, 50% of patients achieved the treatment response criteria (a reduction in score of at least 50% from baseline) by the end of the open-label phase, with 35% achieving clinical remission.

(B) Specific statistical information for each stage in the RCT.

Supplementary Figure 18

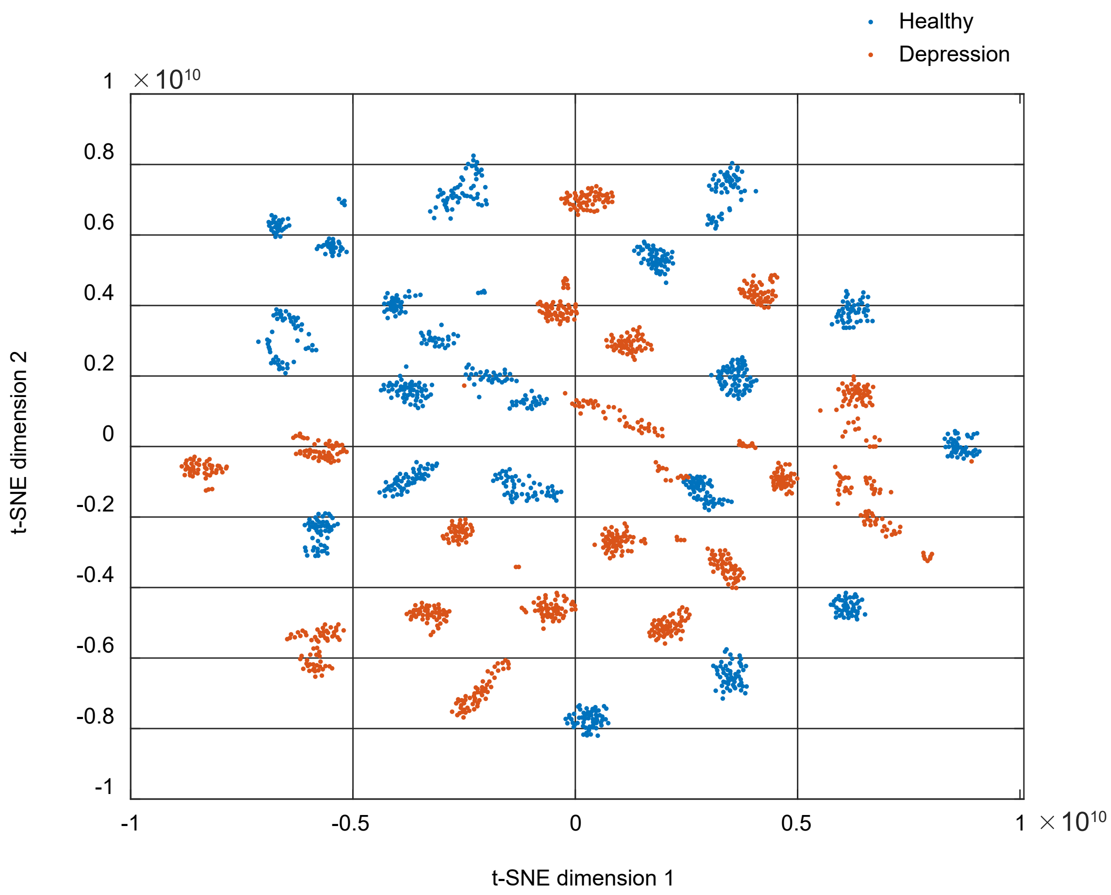

Supplementary Figure 18. Visualization of the fused feature distribution extracted by the deep learning model in two-dimensional t-SNE space. Each point in the figure represents a single LFP sample (epoch) from the independent validation set. For each sample, a 128-dimensional feature vector was obtained by fusing the outputs from the dual-branch (waveform and statistical feature branches) network prior to the classification layer. This vector was then reduced to two dimensions for visualization using the t-SNE (t-distributed Stochastic Neighbor Embedding) algorithm. The points are colored according to their ground-truth neural state labels (Blue: Healthy state; Red: Depression state). The plot clearly shows that samples from the two classes form two spatially distinct and highly separable clusters, with only a few samples appearing in the boundary regions of the opposing cluster. This result provides intuitive evidence that our constructed multi-modal deep learning model successfully learned an internal feature representation capable of effectively discriminating between 'Healthy' and 'Depression' neural states. The significant class separability in the model's high-dimensional feature space provides a solid foundation for the downstream classifier to achieve strong performance.
