# Supplementary material for: Deep Brain Stimulation Induces Antidepressant Effects by Restoring High‐Fidelity Communication in the BNST‐NAc Circuit
Source: Adv Sci (Weinh). 2026 Mar 9;13(25):e21943. doi: 10.1002/advs.202521943 (PMC13137839; doi:10.1002/advs.202521943)
Supplement: Supplementary file 2 — Supporting File 2: advs74542‐sup‐0002‐SuppMat.docx. [file ADVS-13-e21943-s001.docx]

**Methods**

**Clinical study participants**

We recruited 23 patients who met the diagnostic criteria of the ICD-10 (International Classification of Diseases) for recurrent depressive disorder and the following criteria: age between 18 and 65 years old; current duration of depressive episode longer than two years or repeated episodes with more than four episodes (current episode duration ≥ 1 year); treatment-resistant depression, defined as poor response to at least three full-dose and full-course (more than 8 weeks) treatments, including at least two antidepressant drugs with different mechanisms of action and intolerance or poor response to non-convulsive electrotherapy; symptom severity, as measured by a score of at least 17 on the Hamilton Depression Scale-17 (HAMD-17). All participants self-identified as Asian, and their gender identities were consistent with their biological sex. Finally, 18 patients underwent long-term follow-up with LFP data recording, and 3 patients completed follow-up for more than 2 years.

The study protocol was approved by the Ruijin Hospital Ethics Committee of the Shanghai Jiaotong University School of Medicine (Approval Number: 2021-52) and registered at clinicaltrials.gov (NCT04530942). All enrolled patients and their legal representatives were required to sign a formal informed consent before enrollment.

**Clinical outcome assessment**

Prior to DBS surgery, clinical assessments were conducted using well-established scales, including the Hamilton Depression Scale (HAMD-17), Montgomery–Asberg Depression Rating Scale (MADRS), Hamilton Anxiety Scale (HAMA-14), and Pittsburgh Sleep Quality Index (PSQI)**^[1-3]^**. Stimulation was not turned on for the first 4 weeks after surgery. The patients’ depressive symptoms were reassessed 4 weeks after the surgery, which served as the baseline. Following 6 months of DBS treatment, the patients’ symptoms were re-evaluated in both DBS-on and DBS-off states, with the DBS-off state defined as 120 hours after the cessation of stimulation. The duration of the washout period is set at 120 hours because during this parameter titration period, over 90% of patients experience a return of symptoms to baseline levels within 120 hours after shutdown**^[4]^**. Similarly, the reason for defining a 6-month interval for the second assessment and signal collection is that patients had stable symptoms for two consecutive months (score fluctuations less than 10%). Throughout each assessment, two experienced psychiatrists independently evaluated the clinical depression scales, and the final result was determined based on their collective assessments. Depending on their clinical responses, the patients were categorized into the following two groups: 1) the treatment response group, comprising those who exhibited at least a 50% improvement of HAMD scores compared to baseline, and 2) the treatment non-response group, consisting of patients whose HAMD scores improved by less than 50%.

**Surgical procedure**

The pre-surgical plan involved the insertion of electrodes through multiple targets. CT-stereotactic guidance and pre-operative MRI were utilized to implant the DBS leads while patients were under general anesthesia (Figure S1A–C). Model SR1202-S electrodes were employed in this study, with a diameter of 1.27 mm (SceneRay) and consisting of eight cylindrical contacts, each measuring 1.5 mm in length. The adjacent contacts were separated by a distance of 0.5 mm, as previously described (Supplementary Figure10) ^[5,6]^. We used Lead-DBS software to locate the electrodes^[7]^. The location of electrodes and nuclei are presented in Supplementary Figure 10A. The electrode was placed at the NAc and passed through the BNST. To complete the implantation, the implantable pulse generator (IPG; model: SR1103, SceneRay) was subcutaneously placed under the right clavicle.

**Therapeutic deep brain stimulation parameters**

The programming and parameter optimization of the DBS were performed at least 4 weeks after the implantation of the IPG. The optimal stimulation parameters were determined through an individualized titration process. The amplitude was gradually increased at a 0.5 V stepwise from 2 V with fixed frequency (160Hz) and pulse width (90 μs). With the stimulator as anode (+), each electrode, beginning with the most distal one, was stimulated as monopolar cathode (−) with an increasing amplitude to a maximum of 6 V for 30–60 s, provided that no acute adverse effects were elicited. If little clinical improvement was achieved following standardized optimization, we tested voltages greater than 6.0 V and adjustments in pulse width and frequency^[6,8,9]^.

**Clinical participants**

We did this single center, double-blind, randomized controlled and crossover trial at Ruijin Hospital (Shanghai, China). The study was approved by the Ruijin Hospital Ethics Committee, Shanghai Jiao Tong University School of Medicine (protocol available in Supplement 1). The research use of the implanted components was authorized by the Shanghai Testing & Inspection Institute for Medical Devices and approved by China Food and Drug Administration. All patients included provided written informed consent.

Inclusion criteria were as previously described^[6,10,11]^: 1) patient aged 18-65 years; 2) meeting the international Classification of diseases (ICD)-10 definition of non-psychotic major depressive disorder (MDD); 3) current episode ≥ 2 years duration and/or more than 4 repeated episodes with current episode ≥ 1 year duration and a minimum of 5 years since the onset of the first depressive episode; 4) lack of antidepressant response to a minimum of three antidepressant treatments of adequate dose and duration, including at least two medications from two different classes, failure of adequate psychotherapy and electroconvulsive therapy (ECT) (either poor response, intolerance or rejection); 5) remaining stable with the current anti-depressive medicine for the last month and 6) Hamilton Depression Scale-17 (HAMD) score ≥ 17. Exclusion criteria were: 1) schizophrenia or psychosis unrelated to MDD; 2) severe personality disorder, neurological disorders or medical disorders; 3) history of brain surgery; 4) contraindications for anesthesia or stereotactic surgery.

**Clinical study design**

The study started with a longitudinal, open-label trial followed by a randomized, double-blind crossover design. Following electrode implantation, patients underwent an initial open-label stage with parameter optimization for at least six months, during which psychometric assessments will be conducted and documented at each programming session and at three fixed time points: 3 months, 6 months and 12 months post-surgery. Patients should undergo observation for at least two weeks after programming to evaluate the therapeutic efficacy of the selected stimulation parameters. Following the open-label phase, patients were randomly assigned to either receive active DBS for two weeks followed by sham DBS for two weeks (the active-sham group), or vice versa (the sham-active group). Prior to each active and sham phase, a 2-day washout period was implemented. A phase would be terminated prematurely if specific, pre-defined criteria were met. These included: (1) a clinical worsening indicated by an increase of more than 10 points on the HAMD-17 scale compared to the start of that phase, or (2) the patient reporting active suicidal ideation with a plan. Upon meeting either criterion, the current phase was immediately terminated, and after the standard washout period, the patient progressed to the next phase of the crossover trial. Throughout the crossover phase, both medication and DBS parameters were kept constant, and patients were evaluated at five key time points: the pre-randomization phase (T1), two washout phases (T2 and T4), and after each active or sham DBS phase (T3 and T5). We used an envelope procedure for randomization, wherein each randomly assigned group was enclosed within a sealed, opaque envelope. An independent researcher sequentially opened these envelopes and conducted the randomization by switching DBS on or off accordingly. Throughout the trial, patients, two assessors, and other relevant researchers were kept blinded to the stimulation condition.

**Procedures and programming**

All patients received bilateral implants of 8 contact electrodes (SR1202-S; SceneRay). The target was set at the NAc, with the distal 1 or 2 contacts below the NAc and the proximal 1 or 2 contacts close to the dorsal BNST.^[6,10,11]^ The length of each contact is 1.5 mm and the spacing between contacts is 0.5 mm. Target coordinates for the electrode tip were approximately 4-8 mm lateral to the midline, 1-3 mm anterior to the anterior border of the anterior commissure and 5-8 mm inferior to the anterior commissure. Postoperative CT was obtained to assess for intracranial hemorrhage and lead localization. One participant had an additional surgery before randomization and 5 days after first implantation but before randomization to reposition a lead due to the lead not being in the ideal target region. Stimulation was initiated one-week post-surgery and parameters were systematically adjusted approximately every two weeks.^[6]^ Monopolar stimulation was predominantly utilized across the patient cohort. In practice, we increased the amplitude upon observing diminished benefits or limited clinical improvement, potentially reaching 6V. Frequency and pulse width were ubsequently tailored if needed. If these adjustments were ineffective, we introduced the second and, if required, the third preselected contact for monopolar stimulation.

**Wireless LFP recording**

The reference for the LFP signal was initially set as the difference between adjacent electrode contacts. In re-referencing, the reference electrode is set to the common average reference. The selection of recording electrode contact was based on individualized lead-DBS reconstruction results, as shown in Figure 1B. The sampling rate was 1000 Hz. Symptom assessment and LFP recording during the baseline period were conducted subsequent to the patient achieving a state of adequate rest and postoperative recovery. Additionally, the stimulation was deferred until after the accumulation of baseline data. 6 months later, the "DBS-on" status of the LFP is assessed at 10 a.m. As the electrodes are bilaterally implanted, one side is dedicated to delivering DBS pulses while the other is utilized for recording LFP. Then, we kept the DBS off for 120 hour, and we recorded LFP as the “DBS-off” status. For each LFP recording, the duration is 10 minutes. During the LFP recording, the patient sat alone in a magnetically shielded room with their eyes open.

**Preprocessing and power spectrum analysis**

For the analysis, the sampling rate was reset at 600 Hz, and recorded LFP was band-pass filtered at 1–120 Hz (IIR Butterworth filter) with 1 dB of bandpass attenuation to minimize contamination by movement artifact. Power spectral density (PSD) analysis was mainly performed in MATLAB, which was extracted by the method of Welch periodogram (in detail, MATLAB function pwelch). We used a fast Fourier transform (FFT) of 4,096 points with 50% overlap and multi-taper methods (time-bandwidth product = 3, number of tapers = 5) to decrease edge effects. Before further statistical analysis, power from the PSD was normalized. All of the analyses in this period were calculated in MATLAB software (MATLAB 2022a; The MathWorks, Inc., Natick, MA).

**Multivariate Granger causality**

Frequency domain multivariate Granger causality was used to measure directional functional connectivity from BNST to NAc signals, and all the analyses were performed by the multivariate Granger causality (MVGC) toolbox in MATLAB, reported by Barnett and Seth^[15]^ (http://users.sussex.ac.uk/~lionelb/MVGC/html/mvgchelp.html). We down-sampled raw signals to 600 Hz to find the optimized model order for the vector autoregressive (VAR) model. As Granger causality calculation requires stationarity condition of signals, LFP signals were divided into 1-second time-windows with 50% overlap. Bayesian information criterion was performed to calculate the optimized model order for the Granger causality algorithm. The VAR model was built by a manually selected model order, and the auto-covariance sequence was calculated from the above-mentioned VAR model parameters. Then, frequency domain pairwise conditional Granger causality was calculated from the auto-covariance sequence. The frequency resolution of the granger prediction is determined by the model order. We selected model order after a trade-off to provide enough frequency resolution to capture at least one full cycle of oscillations (4 Hz for the lowest frequency). The same model order was used across the cohorts and conditions to avoid bias in statistical comparisons.

**Cross-frequency coupling**

Phase-amplitude coupling modulation index (PAC-MI) was calculated to quantify PAC strength as reported by Tort et al^[16,17]^. First, the recorded LFPs were bandpass filtered at a lower frequency range for phase (1–30 Hz, step width of 1 Hz) and a high-gamma frequency range for amplitude (55–100 Hz, step width of 1 Hz) using an IIR Butterworth filter. Then, the instantaneous phase and amplitude were calculated by the Hilbert transform. Extracted phase series were then divided into 36 bins (each included 20°, from 0° to 720°), and the mean instantaneous amplitude over each phase bin was calculated.

$P\left( i \right)=\frac{\left\langle A_{f_{A}} \right\rangle_{\phi_{f_{p}}}\left( i \right)}{\sum_{k=1}^{N} \left\langle A_{fA} \right\rangle_{\phi_{f_{p}}}\left( k \right)},$ (1)

where N is the count of phase bins, $\phi_{f_{p}}(t)$ is a time series of phases and $A_{f_{A}}(t)$ is amplitude extracted by Hilbert. The mean amplitude $A_{f_{A}}(t)$ at the phase bin *I* was denoted by $\left\langle A_{fA} \right\rangle_{\phi_{f_{p}}}\left( i \right)$.

Based on this calculation, PAC-MI was then defined as the overall entropy of this phase-amplitude distribution, and normalized distribution was obtained using the maximum entropy value. Band-passed signal coupling was computed for phase and amplitude represented on a modulation index plot (shown in Figure 5B).

Shannon entropy based on the P(*i*) above was given by:

$H\left( P \right)=-\sum_{i=1}^{N} P\left( i \right)\cdot\log\left[ P\left( i \right) \right],$ (2)

and the normalization of Shannon entropy was calculated as follows:

$MI=\frac{\log\left( N \right)-H(P)}{log(N)},$ (3)

where $\log\left( N \right)$ is the maximal value of Shannon entropy. This method was chosen for its tolerance to noise, independence of the original signal, and greater sensitivity to the strength of cross-frequency coupling without the need for an obvious peak in power spectra.

**Sample entropy and weighted phase lag index**

Sample entropy was used to measure stimulus-induced electrophysiological signal complexity perturbations. Among the various methods of calculating entropy, the sample entropy method was chosen because of its statistical accuracy. Due to the narrow time windows in the analysis (1000 sampling points, shown in Figure 7C and 7D), the sample entropy method is not dependent on data length and has better consistency^[18]^.

$Sample Entropy=\lim_{N\to\infty} (-log\frac{\frac{1}{N-m}\sum_{i=1}^{N-m} P_{i}^{m}\left( X_{i} \right)}{\frac{1}{N-m-1}\sum_{i=1}^{N-m-1} P_{i}^{m}\left( X_{j} \right)}),$ (4)

where *N* is the length of the LFP data, *P* is probability distribution, *m* is the length of $X_{i}$ and $X_{j}$.

Weighted phase lag index (wPLI) coherence was used to explore transient synchrony in the BNST-NAc circuit and was calculated for the filtered frequencies (0.1–100 Hz). As reported by Vinck et al., the wPLI is a metric utilized to assess the degree of non-equiprobability of phase relationships between two signals. It has been demonstrated that the wPLI is immune to the impact of volume conduction that may arise from independent sources or active reference^[19]^.

$wPLI=\frac{\left| \sum_{t=1}^{n} |imag(S_{xy,t})|sgn(imag(S_{xy,t})) \right|}{\sum_{t=1}^{n} |imag(S_{xy,t})|},$ (5)

where *x*(*t*) and *y*(*t*) were two time series, and *t* was time points with a rate of 1,000 samples per second.

**Parameterizing Neural Power Spectra analysis**

To disentangle neural oscillations (periodic components) from the background 1/f-like aperiodic activity, we employed the Parameterizing Neural Power Spectra algorithm (specparam, formerly known as FOOOF) ^[20]^. This algorithm models the neural power spectrum, *P(f)*, as a linear combination of an aperiodic component, L(f), and N Gaussian functions, $G_{n}(f)$*,* which represent periodic oscillations:

*P(f)=L(f)+*$\sum_{n=0}^{N} G_{n}(f)$, (6)

The aperiodic component, L(f), is modeled as an exponential function in semi-log space (log-power, linear-frequency):

*L(f)=b−log(k+*$f^{\chi}$*)*, (7)

This function is defined by three parameters: the broadband power offset (b), the knee (k), and the aperiodic exponent (χ). Each periodic component (i.e., an oscillation) is characterized by a Gaussian function, G(f), defined by its center frequency (CF), power (Pw​), and bandwidth (BW). Using the specparam Python library (version 1.1.0), we fitted the power spectral density (PSD) curve from each electrode across a frequency range of 1–100 Hz. The primary settings for the specparam model were configured as follows: peak_width_limits: (1.5, 12.0). This constrains the bandwidth of oscillatory peaks to between 1.5 Hz and 12.0 Hz to capture physiologically plausible neural oscillations. max_n_peaks: 6. This limits the maximum number of oscillations detected within the specified frequency range to six. min_peak_height: 0.1. A peak was only considered a genuine oscillation if its power was at least 0.1 log10 units above the aperiodic component. aperiodic_mode: 'knee'. This setting utilizes the standard knee-less model to fit the aperiodic component.The goodness of fit for each model was evaluated using the coefficient of determination (R^2^) and the root mean square error (RMSE). Only fits with an R^2^>0.95 were included in subsequent analyses.Following successful model fitting, we extracted the parameters of interest from the periodic components of the model's output. For each electrode in the BNST, we searched the model results for an oscillatory peak with a center frequency in the theta band (4–12 Hz). If multiple peaks were detected within this band, the one with the highest power was selected. Similarly, for each electrode in the NAc, we searched for a peak with a center frequency in the low-gamma band (30–55 Hz). Concurrently, we also extracted the aperiodic exponent (χ) from each spectrum to assess dynamic changes in the background neural activity.

**Deep brain stimulation for mice**

This study employed DBS to modulate the BNST. Electrical stimulation was delivered via a programmable, constant-current isolated stimulator connected to the implanted electrodes. After all experiments were concluded, animals were perfused, and brain tissues were sectioned to histologically verify the precise location of the electrode tips under a microscope, confirming their placement within the target nucleus. Only data from animals with accurately verified electrode positions were included in the final analysis. The stimulation paradigm was high-frequency stimulation. The stimulation waveform consisted of charge-balanced, symmetric biphasic square-wave pulses to minimize the net accumulation of charge at the tissue-electrode interface, thereby reducing the risk of tissue damage. The stimulation frequency was constant at 130 Hz, and the pulse width was fixed at 90 µs. Prior to the formal experiment, a current intensity titration procedure was performed for each animal to determine its individualized optimal therapeutic current. Titration began at a low current of 10 µA and was gradually increased in 10 µA increments. At each current level, it was observed that the intensity did not cause any observable side effects such as muscle twitching, freezing, or signs of distress. The final therapeutic current for all animals was set to 100 µA.

This study designed and implemented a closed-loop DBS control algorithm based on real-time neuronal firing rates to trigger therapeutic stimulation of a fixed duration on an as-needed basis. The algorithm's workflow primarily consists of three stages: signal preprocessing, biomarker extraction, and state-dependent stimulation control.

First, in the signal preprocessing stage, the raw neural signal x_raw_​(t) acquired from the microelectrode array was filtered using a fourth-order digital Butterworth band-pass filter with a passband of 300-3000 Hz. This effectively isolated spike activity while suppressing low-frequency LFP and high-frequency noise. The filtered discrete-time signal is denoted as x(n).Second, in the biomarker extraction stage, we calculated the instantaneous firing rate (FR)^[21]^. To enhance the SNR of the spikes, we first applied the Teager-Kaiser Energy Operator (TKEO) to the signal x(n), generating an instantaneous energy sequence *Ψ(n)*. The calculation is as follows:

*Ψ[x(n)] = x(n) ^2^ - x(n+1) ·x(n-1)* , (8)

Subsequently, spike events were detected from the energy sequence *Ψ(n)* using an adaptive threshold $\theta$. This threshold is based on a robust estimation of the signal noise, determined by calculating the median absolute deviation (MAD) of the *Ψ(n)* sequence to estimate the standard deviation of the noise σ*_Ψ_*. This approach prevents the spikes themselves, as outliers, from interfering with the noise estimation. The threshold *θ* was calculated as:

σ*_Ψ_≈*$\frac{median(\left| \Psi\left( n \right)-median(\Psi) \right|)}{0.6745}$*, θ=k·*σ*_Ψ_*, (9)

Here, k is an empirical sensitivity coefficient (set to 5 in this study). A spike event was detected when the value of Ψ(n) exceeded the threshold θ. Finally, the system calculated the real-time FR by counting the number of detected spikes (N_spikes_​) within a 50ms sliding time window of length W:

FR= ​​$\frac{\mathrm{Nspikes}}{W}$, (10)

Finally, in the state-dependent stimulation control stage, the algorithm operated as a three-state (monitoring, stimulating, lockout) finite-state machine. In the default 'monitoring' state, the system continuously calculated the FR and compared it to a preset pathological trigger threshold, FR_trigger_​. When FR>FR_trigger_​, the system immediately switched to the 'stimulating' state, initiating a DBS pulse train with a fixed duration T_burst​_ of 200 ms. During this period, signal acquisition and analysis were paused to avoid stimulation artifacts. After the stimulation ended, the system automatically entered a lockout state for a fixed duration T_lockout​_ of 500 ms. During this refractory period, the system performed no analysis or stimulation to prevent false triggers on post-stimulation artifacts and to allow the neural circuit time to stabilize. After the lockout period, the system returned to the 'monitoring' state, beginning a new cycle of closed-loop monitoring and control. For inhibitory period closed-loop DBS, we first excluded neurons exhibiting hyperactivity due to stress (defined as a firing rate exceeding the mean plus two standard deviations of the naïve animal BNST firing rate). The remaining neurons were monitored. If the firing rate was determined to be 0 within a continuous 200 ms time window, the system identified this as an inhibitory period and immediately triggered DBS^[22]^. Random delay DBS was defined as initiating DBS with a random delay of 500-1000 ms after an inhibitory period was detected, serving as a study control. E-state delay DBS was defined as initiating DBS after an inhibitory period was detected, but delayed until the firing rate exceeded 4 Hz within the next 200 ms time window. Full-time DBS was defined as continuous, uninterrupted DBS. All animals received a total of 60 minutes of monitoring and stimulation per day for five consecutive days.

**Machine learning for UP and DOWN states classification**

This part aimed to classify neuronal states, defined as UP, DOWN, or Intermediate based on single-neuron spiking activity, by applying machine learning models to synchronously recorded LFP signals. All signal processing and machine learning analyses were conducted in MATLAB (R2024b) utilizing the Statistics and Machine Learning Toolbox™. Initially, raw LFP signals sampled at 2000 Hz were segmented using a 500 ms sliding window with 75% overlap. Discrete neuronal states were then defined based on the spike count within each window: windows with three or more spikes were labeled as UP states, those with exactly two spikes were labeled as Intermediate states, and those with one or fewer spikes were labeled as DOWN states. For each labeled window, a set of features was extracted from the corresponding LFP segment, including: sample entropy to quantify signal complexity, calculated with an embedding dimension of m=2 and a tolerance radius of r=0.2×σ (where σ is the signal's standard deviation); the aperiodic (1/f) slope, derived by calculating the power spectral density via Welch's method and then performing a linear fit on the log-log plot of power versus frequency in the 2-100 Hz range; and the relative power in six standard frequency bands (delta: 1-4 Hz, theta: 4-8 Hz, alpha: 8-12 Hz, beta: 12-30 Hz, low gamma: 30-60 Hz, and high gamma: 60-100 Hz). To build the classification models, all feature vectors were aggregated. Subsequently, the dataset was partitioned into training and testing sets, and a Z-score standardization was applied to the training set, with the same transformation parameters used for the test set to prevent data leakage. Four supervised classifiers were trained, with hyperparameters optimized via 10-fold cross-validation: a pseudo-linear Discriminant Analysis (LDA) with regularization; a k-Nearest Neighbors (k-NN) classifier optimized via grid search for the number of neighbors; a Support Vector Machine (SVM) with a Gaussian RBF kernel, whose box constraint and kernel scale were tuned using Bayesian optimization; and a Random Forest model composed of 50 trees, with its minimum leaf size optimized through cross-validation. Finally, model performance was evaluated on the independent test set using metrics derived from the confusion matrix, including accuracy, precision, recall, F1-score, and specificity. The overall discriminative ability of the classifiers was compared using Receiver Operating Characteristic (ROC) curves and the Area Under the Curve (AUC). To interpret the models, the contribution of each LFP feature was quantified using the Random Forest's permuted predictor importance algorithm on out-of-bag (OOB) data.

**Transformer-based algorithm for depressive-state classification**

We design and validate a multi-modal deep learning model developed to fuse LFP time-series data with synchronously extracted tabular features for the high-accuracy classification of 'healthy' versus 'depressive' neurophysiological states. To prevent data leakage and ensure rigorous validation, the dataset was strictly partitioned by study phase. The Training Set consisted of LFP recordings from the open-label optimization phase (n=18 patients). The Validation Set consisted exclusively of data from the blinded RCT phase (n=10 patients with complete artifact-free recordings). No RCT data was used to train the model or select feature thresholds. The model was developed using a dataset of 198 trials (1800 seconds per trial) from 18 subjects and evaluated under a stringent 18-fold leave-one-subject-out cross-validation (LOSO-CV) scheme; furthermore, an independent validation set, consisting of 480 trials (240 healthy states, 240 depressive states, 1800 seconds per trial) from 10 subjects, was utilized to assess generalization. The dataset's LFP signals were dual-channel, recorded from the BNST and NAc at a sampling frequency of 1000 Hz. The signals were segmented into 4-second epochs, for which a corresponding set of tabular features was computed, including weighted gamma power and oscillation in the NAc, weighted theta power and oscillation in the BNST, E state and its connectivity, and I state and its connectivity. The model employs an end-to-end architecture composed of three synergistic sub-networks: a waveform network processes raw LFP data using two convolutional layers followed by a bidirectional long short-term memory (Bi-LSTM) network with 128 hidden units, projecting the time-series into a 64-dimensional feature vector; concurrently, a tabular network, based on an FT-Transformer architecture with two modules and eight attention heads each, encodes the tabular features into a separate 64-dimensional vector. Finally, a classifier network concatenates these vectors into a 128-dimensional feature space, which is processed by a fully-connected layer with a dropout rate of 0.5 before a softmax layer outputs the final class probabilities. The model was trained for 2000 epochs per fold using the Adam optimizer with a learning rate of 0.01, a gradient decay factor of 0.9, a squared gradient decay factor of 0.999, and a mini-batch size of 16. To enhance model generalization, minor additive Gaussian noise and multiplicative scaling were applied exclusively to the LFP data within the training set of each fold. Following training in each fold, the model's performance was dually assessed on both the held-out subject (the LOSO test set) and the independent validation set. Overall performance was evaluated by aggregating the results from all 18 folds, utilizing metrics such as overall accuracy, a confusion matrix, and the receiver operating characteristic (ROC) curve with its area under the curve (AUC). Additionally, the t-Distributed Stochastic Neighbor Embedding (t-SNE) algorithm was used to visualize the 128-dimensional fused features to qualitatively assess class separability.

**Animals and surgery**

Adult male C56BL/6J mice, aged 8-10 weeks and weighing 18-22g, were used in this study. 9 mice were used for baseline electrophysiological characterization. 17 mice were used for the learned helplessness modeling phase. Because the behavior phenotype of the learned helplessness model disappear over time, 22 mice were screened during the DBS stimulation phase. The mice were bred and housed in a specific-pathogen-free (SPF) grade animal facility. All mice were maintained in an environment at 22°C ± 2°C with a 12-hour light-dark cycle and had ad libitum access to food and water. All procedures were conducted in strict accordance with the ethical guidelines of the Institutional Animal Care and Use Committee (IACUC). Experimental animals were anesthetized via inhalation of 1.5-2.0% isoflurane and then secured in a stereotaxic apparatus for precise stereotaxic fixation. A 128-channel flexible electrode array (Model: NXDB-2×16-6mm-25-1616, NeuroXess) was used for simultaneous implantation into the Bed Nucleus of the Stria Terminalis (BNST) (coordinates: AP +0.26 mm, ML ±0.65 mm, DV -3.70 mm from the dura mater) and the Nucleus Accumbens (NAc) (coordinates: AP +1.10 mm, ML ±1.60 mm, DV -4.0 mm from the dura mater). The electrode assembly and a skull fixation system were secured using medical-grade dental cement. Post-surgery, animals were housed individually, administered antibiotics to prevent infection, and monitored for 7-10 days to ensure full recovery. After the animals' behavior returned to a baseline level, a high-throughput neural signal acquisition system (Model: NSPS4, NeuroXess) was used to record neural electrical signals from freely-behaving mice at a sampling rate of 30 kHz.

**Data acquisition and processing**

The raw data underwent spike sorting using the valley-seeking algorithm. This algorithm employs common average referencing (CAR) for preprocessing and utilizes its built-in drift correction function and template-matching strategy to accurately isolate the action potential trains of single neurons. The sorted results were manually curated. Only single-unit clusters that met strict quality criteria, such as an inter-spike interval (ISI) violation rate of less than 0.5% within a 2 ms refractory period, were included in subsequent analyses. We classified the validated neurons based on average waveform features. Units with a trough-to-peak time greater than 0.45 ms were classified as putative GABAergic neurons, while those with a time less than or equal to 0.45 ms were classified as putative interneurons. After all experiments were completed, the accuracy of the electrode recording sites was confirmed through histological verification using electrolytic lesion marking (parameters: current 2mA, pulse width 3.8 ms, frequency 130 Hz, bidirectional square wave), followed by perfusion and cryosectioning. LFPs were derived from wideband signals by downsampling all channels to 2000 Hz.

**Electrode Tip Localization**

To precisely confirm the electrode implantation site, mice were transcardially perfused with 4% PFA post-experiment, and brains were cryoprotected in sucrose before being cryosectioned at 40 µm. Sections were processed for multiplex immunofluorescence using primary antibodies against NeuN (rabbit-anti-NeuN) and GFAP (mouse-anti-GFAP), followed by corresponding secondary antibodies: Goat anti-Rabbit Alexa Fluor 488 (Green, marking neurons) and Goat anti-Mouse Alexa Fluor 594 (Red, marking astrocytes). Nuclei were counterstained with DAPI (Blue). Finally, images were acquired via confocal microscopy; the electrode tract was identified as a channel void of DAPI signal and bordered by strong GFAP (Red) signal, while the tip location was confirmed by identifying the tract's end, which was characterized by significant neuronal loss (absence of NeuN, Green) and prominent reactive gliosis (strong upregulation of GFAP, Red), and correlated with a mouse brain atlas for final localization.

**Depression-like phenotype training**

All mice were single housed for one week of habituation before the experiment. The experimental apparatus was a two-chamber shuttle box connected to a programmable conditioned stimulus controller (Model: XR-XTS101, Shanghai Xinruan). Before the formal induction, mice were placed in the shuttle box with the connecting door open and allowed to explore freely for 15 minutes to acclimate to the equipment. The learned helplessness induction procedure was conducted for five consecutive days. Each day, mice were placed in one side of the shuttle box with the connecting door closed and 360 inescapable foot shocks were administered (0.3 mA intensity, 1-3 seconds random duration, 1-15 seconds random interval). A behavioral test was conducted 24 hours after the third induction session. For the test, mice first explored the shuttle box freely for 5 minutes with the connecting door open. This was followed by 30 escapable foot shock trials. Each trial consisted of a 5-second light cue. If the mouse did not respond, a foot shock of up to 10 seconds (0.3 mA) was immediately administered. The interval between trials was 30 seconds. The stimulus was terminated as soon as the mouse moved to the opposite chamber during either the cue or the shock. The active avoidance, escape latency, and number of escape failures were automatically recorded throughout the experiment. Individuals with more than 15 escape failures out of the 30 trials were defined as the learned helplessness susceptible phenotype, while the remainder were defined as the resilient phenotype.

**Delta spikes recognition**

Delta waves were detected using the following method. The recorded BNST LFP was first filtered for the corresponding frequency band to obtain the raw 0.1-5 Hz signal, which was then standardized. For a waveform to be classified as a delta wave, its duration had to be between 150 and 500 ms, and its signal peak had to be greater than two standard deviations, or its signal peak had to be greater than one standard deviation while its signal trough was less than negative two standard deviations.

To analyze the relationship between neuronal firing and delta waves, we first distinguished between delta waves associated with spikes and silent delta waves, using a previously reported method. We used a conservative 30 ms threshold to define delta-related firing, which included any spike occurring within 30 ms of a delta wave peak detected in the same brain region. Delta waves without any associated delta-related firing were classified as silent delta waves. It was observed that most recent spikes occurred 100–200 ms from the delta wave peak, a period considered not to be in the DOWN-state, although a small fraction of spikes occurred within 15 ms of the peak. The time delay was measured between the peak of each delta wave and the nearest spike from any neuron recorded in the same brain region. The firing distributions of the two neuron types during delta waves were entirely different (see Supplementary Figure 1A-B).

We adapted a method reported in previous literature to assess the predictive power of NAc gamma rhythmic activity for BNST neuronal firing^[23]^. This method aims to quantify how accurately the LFP gamma power in the NAc can predict the firing activity of a single BNST neuron during a BNST delta wave. For each BNST neuron, we first recorded its firing activity during each delta wave event as a binary vector (1=spike, 0=no spike). The mean gamma power from the synchronously recorded NAc LFP during the corresponding period was used as the predictor variable. Assuming the neuron fired during a total of k delta waves, we divided all delta wave events into k non-overlapping sets, ensuring that each set contained exactly one firing event from that neuron.

Next, we employed a k-fold cross-validation scheme consistent with previous report^[24]^. We trained a GLM on a training set composed of k−1 sets, using the NAc gamma power to predict the binary firing vector of the BNST neuron. Subsequently, model performance was evaluated on the remaining unused set (the test set). This process was repeated k times, ensuring each set was used as the test set exactly once. We assessed the prediction quality by comparing the median of the model prediction error, e, with the median error from a dataset shuffled 1,000 times, $e_{\mathrm{shuffled}}$. The shuffled error was obtained by randomly matching the model's predictions with the actual observed data. Finally, the prediction gain, g, was defined as:

g=$\frac{e_{\mathrm{shuffled}}}{e}$ ,(11)

A value of g significantly greater than 1 indicates that NAc gamma power contains predictive information about the delta-wave-related firing of BNST neurons beyond the chance level.

For the comparative analysis of features across inhibitory and excitatory periods (Figure 1M–O), a paired under sampling strategy was adopted to account for the sparse distribution of inhibitory periods relative to excitatory ones and to prevent biases in statistical power. First, inhibitory periods were identified based on the troughs of delta waves in the BNST LFP. For each identified inhibitory period, a control excitatory period of identical duration was selected at a minimum 180° phase offset. This approach ensured that the two functional states were equivalent in both event count and total observation duration, thereby providing a balanced statistical basis for subsequent analyses of circuit connectivity (wPLI), signal complexity (sample entropy), and power spectral density (PSD) slope.

**Cross-correlations**

The cross-correlation between the firing activity of neurons in the BNST and the NAc was calculated as follows. First, we identified delta waves in the BNST LFP signal and used their peak times (t_delta_BNST_​) as reference events. For each BNST neuron i, we created a spike count vector, S_BNST,i​_, by counting its spikes within a narrow window (30 ms) centered on each t_delta_BNST_​. Similarly, for each NAc neuron j, we generated a spike count vector, S_NAc,j​_(k), by counting its spikes within a wider window (200 ms) centered at different time lags (t_delta_BNST​_+k). The time lag k ranged from -500 to 500 ms. Subsequently, we calculated the spearman rank-order correlation coefficient, ρ_ij_​(k), between the spike count vectors S_BNST,i​_ and S_NAc,j_​(k) for each cross-regional neuron pair (i, j). The results were then averaged across all cell pairs to obtain the mean cross-correlation, ρ(k), as a function of the time lag k.

To assess the statistical significance of this cross-correlation, we created a control (shuffled) cross-correlation, ρ_ij_′​(k), by applying a single-event shift to the time series of one of the neurons. Specifically, we correlated the firing activity of a BNST neuron during the n-th delta wave with the activity of an NAc neuron during the (n+1)-th delta wave. This single-event shift method selectively disrupts correlations on a fine temporal scale while preserving slower trends that may exist in the data, such as changes in the animal's behavioral state. This approach effectively prevents the emergence of false positives. Finally, we defined the enrichment of the correlation as the difference between the observed and the shuffled correlations, namely ρ(k)−ρ′(k). This value served as the final measure of cross-correlation strength.

**Predicted spikes and signal-to-noise ratio**

This analysis begins by identifying BNST-NAc neuron pairs that exhibit a significant coupling relationship. For each BNST neuron i and NAc neuron j, we calculated their spike counts within event windows centered on BNST delta waves. Following the logic of the original method, we defined a BNST cell i and an NAc cell j as 'partner cells' if a significant spearman rank-order correlation existed between their spike count vector S_BNST,i_​(k) and the time-lagged spike count vector of the NAc cell, S_NAc,j​_(k−200 ms). Here, k represents each individual delta wave event. This -200 ms time lag signifies that we were searching for cell pairs where the activity of the NAc neuron could predict the subsequent activity of the BNST neuron. Next, for each BNST neuron identified as having a partner, we classified its individual spikes. A BNST spike was classified as a 'partner spike' if any of that neuron's NAc partner cells fired within the 200 ms window preceding the spike. All other BNST spikes were considered 'non-partner spikes' or background activity. The signal-to-noise ratio was then calculated as the ratio of the number of partner spikes to the number of all other spikes within a 20 ms time window centered on the delta wave peak. For the human LFP data, the signal-to-noise ratio was defined as the ratio of the power in the 1-100 Hz frequency band to the power in the 1-200 Hz frequency band within a specific time window^[23]^.

**Statistics**

Statistical analyses were performed using GraphPad Prism 8.0, MATLAB (R2024b) with custom scripts and the FieldTrip toolbox, and Python 3.8. Unless otherwise specified, an experimenter blinded to the experimental conditions conducted the data analysis. Key statistical features, including the definitions and exact values of n, specific p-values, and the type of statistical tests, can be found in the corresponding Figure legends or Supplementary Materials. In all box plots, the center line represents the median, the box boundaries represent the 25th and 75th percentiles, and the whiskers extend to the lowest and highest data points in the set. The horizontal line within the plot represents the mean.

For direct comparisons between two related groups, we conducted paired t-tests. For comparisons among three or more experimental groups, we conducted repeated measures one-way ANOVAs followed by Bonferroni's post-hoc test for multiple comparisons. All statistical tests were two-tailed, and the confidence interval was set at 95%. Statistical significance was defined as $p < 0.05$. In all box plots, the center line represents the median, the box boundaries represent the approximate quartiles, and the whiskers extend to the lowest and highest data points. The horizontal line within the plot represented the mean.

For analysis of Local Field Potential (LFP) power spectra, we used the specparam toolbox in Python to parameterize the spectra into their periodic (oscillatory peaks) and aperiodic (1/f-like slope) components. This method involves fitting a model to the power spectrum to extract features such as the aperiodic offset and exponent, as well as the center frequency, power, and bandwidth of oscillatory peaks. Statistical comparisons of these derived parameters between conditions were conducted using the appropriate tests mentioned above.

For continuous waveform data, we employed a cluster-based Monte Carlo permutation test to correct for multiple comparisons across time points and avoid false positives. This non-parametric method first identifies clusters of contiguous time points where the signals from two experimental conditions differ significantly (point-wise p < 0.05). To determine the significance of these observed clusters, a null distribution was generated by randomly permuting the condition labels across trials 1000 times and extracting the maximum cluster-level statistic from each permutation. An observed cluster in the real data was considered statistically significant if its statistic exceeded the 95th percentile of the permuted null distribution (p < 0.05). In our analyses, only significant clusters where the signal difference persisted for a continuous duration of 50 ms or longer were marked and considered for final interpretation.

# References

[1] Hamilton M. A rating scale for depression[J]. J Neurol Neurosurg Psychiatry, 1960, 23(1): 56-62.

[2] Zimmerman M, Chelminski I, Posternak M. A review of studies of the Montgomery-Asberg Depression Rating Scale in controls: implications for the definition of remission in treatment studies of depression[J]. Int Clin Psychopharmacol, 2004, 19(1): 1-7.

[3] Buysse D J, Reynolds C F, 3rd, Monk T H, et al. The Pittsburgh Sleep Quality Index: a new instrument for psychiatric practice and research[J]. Psychiatry Res, 1989, 28(2): 193-213.

[4] Bergfeld I O, Mantione M, Hoogendoorn M L, et al. Deep Brain Stimulation of the Ventral Anterior Limb of the Internal Capsule for Treatment-Resistant Depression: A Randomized Clinical Trial[J]. JAMA Psychiatry, 2016, 73(5): 456-64.

[5] Xiong B, Wen R, Gao Y, et al. Longitudinal Changes of Local Field Potential Oscillations in Nucleus Accumbens and Anterior Limb of the Internal Capsule in Obsessive-Compulsive Disorder[J]. Biol Psychiatry, 2021.

[6] Lai Y, Dai L, Wang T, et al. Structural and functional correlates of the response to deep brain stimulation at ventral capsule/ventral striatum region for treatment-resistant depression[J]. J Neurol Neurosurg Psychiatry, 2023, 94(5): 379-388.

[7] Horn A, Kühn A A. Lead-DBS: a toolbox for deep brain stimulation electrode localizations and visualizations[J]. Neuroimage, 2015, 107: 127-135.

[8] Torres C V, Ezquiaga E, Navas M, et al. Deep brain stimulation of the subcallosal cingulate for medication-resistant type I bipolar depression: case report[J]. Bipolar Disord, 2013, 15(6): 719-21.

[9] Edemann-Callesen H, Voget M, Empl L, et al. Medial Forebrain Bundle Deep Brain Stimulation has Symptom-specific Anti-depressant Effects in Rats and as Opposed to Ventromedial Prefrontal Cortex Stimulation Interacts With the Reward System[J]. Brain Stimul, 2015, 8(4): 714-23.

[10] Wang T, Dai L, Lai Y, et al. Parameter-based analysis of clinical efficacy of combined bed nucleus of the stria terminalis-nucleus accumbens deep brain stimulation for treatment-resistant depression[J]. J Neurosurg, 2024: 1-11.

[11] Wang F, Dai L, Wang T, et al. Presurgical structural imaging and clinical outcome in combined bed nucleus of the stria terminalis-nucleus accumbens deep brain stimulation for treatment-resistant depression[J]. Gen Psychiatr, 2024, 37(3): e101210.

[12] Montgomery S A, Asberg M. A new depression scale designed to be sensitive to change[J]. Br J Psychiatry, 1979, 134: 382-9.

[13] Hamilton M. The assessment of anxiety states by rating[J]. Br J Med Psychol, 1959, 32(1): 50-5.

[14] Rizvi S J, Quilty L C, Sproule B A, et al. Development and validation of the Dimensional Anhedonia Rating Scale (DARS) in a community sample and individuals with major depression[J]. Psychiatry Res, 2015, 229(1-2): 109-19.

[15] Barnett L, Seth A K. The MVGC multivariate Granger causality toolbox: a new approach to Granger-causal inference[J]. J Neurosci Methods, 2014, 223: 50-68.

[16] Tort A B, Komorowski R, Eichenbaum H, et al. Measuring phase-amplitude coupling between neuronal oscillations of different frequencies[J]. J Neurophysiol, 2010, 104(2): 1195-210.

[17] Tort A B, Kramer M A, Thorn C, et al. Dynamic cross-frequency couplings of local field potential oscillations in rat striatum and hippocampus during performance of a T-maze task[J]. Proc Natl Acad Sci U S A, 2008, 105(51): 20517-22.

[18] Richman J S, Moorman J R. Physiological time-series analysis using approximate entropy and sample entropy[J]. Am J Physiol Heart Circ Physiol, 2000, 278(6): H2039-49.

[19] Vinck M, Oostenveld R, Van Wingerden M, et al. An improved index of phase-synchronization for electrophysiological data in the presence of volume-conduction, noise and sample-size bias[J]. Neuroimage, 2011, 55(4): 1548-65.

[20] Donoghue T, Haller M, Peterson E J, et al. Parameterizing neural power spectra into periodic and aperiodic components[J]. Nat Neurosci, 2020, 23(12): 1655-1665.

[21] Krabben T, Prange G B, Kobus H J, et al. Application of the Teager-Kaiser Energy Operator in an autonomous burst detector to create onset and offset profiles of forearm muscles during reach-to-grasp movements[J]. Acta Bioeng Biomech, 2016, 18(4): 135-144.

[22] Johnson L A, Euston D R, Tatsuno M, et al. Stored-trace reactivation in rat prefrontal cortex is correlated with down-to-up state fluctuation density[J]. J Neurosci, 2010, 30(7): 2650-61.

[23] Todorova R, Zugaro M. Isolated cortical computations during delta waves support memory consolidation[J]. Science, 2019, 366(6463): 377-381.

[24] Harris K D, Csicsvari J, Hirase H, et al. Organization of cell assemblies in the hippocampus[J]. Nature, 2003, 424(6948): 552-6.
